# A Quantitative Proteomics View on the Function of *Qfhb1*, a Major QTL for Fusarium Head Blight Resistance in Wheat

**DOI:** 10.3390/pathogens7030058

**Published:** 2018-06-22

**Authors:** Moustafa Eldakak, Aayudh Das, Yongbin Zhuang, Jai S. Rohila, Karl Glover, Yang Yen

**Affiliations:** 1 Department of Biology and Microbiology, South Dakota State University, Brookings, SD 57006, USA; moustafa.eldakak@gmail.com (M.E.); Aayudh.Das@uvm.edu (A.D.); zyb1986@sdau.edu.cn (Y.Z.); 2 Genetics Department, College of Agriculture, Alexandria University, Alexandria 21526, Egypt; 3 Department of Plant Biology, University of Vermont, Burlington, VT 05405, USA; 4 College of Agronomy, Shandong Agricultural University, Taian 271018, China; 5 Department of Agronomy, Horticulture, and Plant Science, South Dakota State University, Brookings, SD 57006, USA; Karl.Glover@sdstate.edu; 6 Dale Bumpers National Rice Research Center, Stuttgart, AR 72160, USA

**Keywords:** FHB, fhb1, FHB pathogeneses, FHB resistance, fusarium, hypersensitive response, proteomics, scab, wheat

## Abstract

Fusarium head blight (FHB) is a highly detrimental disease of wheat. A quantitative trait locus for FHB resistance, *Qfhb1*, is the most utilized source of resistance in wheat-breeding programs, but very little is known about its resistance mechanism. In this study, we elucidated a prospective FHB resistance mechanism by investigating the proteomic signatures of *Qfhb1* in a pair of contrasting wheat near-isogenic lines (NIL) after 24 h of inoculation of wheat florets by *Fusarium graminearum*. Statistical comparisons of the abundances of protein spots on the 2D-DIGE gels of contrasting NILs (fhb1+ NIL = *Qfhb1* present; fhb1- NIL = *Qfhb1* absent) enabled us to select 80 high-ranking differentially accumulated protein (DAP) spots. An additional evaluation confirmed that the DAP spots were specific to the spikelet from fhb1- NIL (50 spots), and fhb1+ NIL (seven spots). The proteomic data also suggest that the absence of *Qfhb1* makes the fhb1- NIL vulnerable to *Fusarium* attack by constitutively impairing several mechanisms including sucrose homeostasis by enhancing starch synthesis from sucrose. In the absence of *Qfhb1*, *Fusarium* inoculations severely damaged photosynthetic machinery; altered the metabolism of carbohydrates, nitrogen and phenylpropanoids; disrupted the balance of proton gradients across relevant membranes; disturbed the homeostasis of many important signaling molecules induced the mobility of cellular repair; and reduced translational activities. These changes in the fhb1- NIL led to strong defense responses centered on the hypersensitive response (HSR), resulting in infected cells suicide and the consequent initiation of FHB development. Therefore, the results of this study suggest that *Qfhb1* largely functions to either alleviate HSR or to manipulate the host cells to not respond to *Fusarium* infection.

## 1. Introduction

Fusarium head blight (FHB; caused by ascomycetous fungi *Fusarium* species) is a common fungal disease of wheat (*Triticum aestivum* L.) and many other cereal crops in both semitropical and temperate regions worldwide. It can cause devastating reductions in both grain yield and quality. The pathogen deposits mycotoxins, primarily deoxynivalenol (DON), which make the infected grains unsuitable for human and animal consumption [1,2]. While many *Fusarium* fungi can cause FHB, *F. graminearum* is the predominant FHB pathogen in North America and many other places across the world [3]. The two types of FHB resistance generally accepted in FHB research community are: against the initial infection of the host (Type I) or against pathogen’s spread within the host (Type II), as proposed by Schroeder and Christensen [4]. Type I resistance is most likely due to spike’s morphology and possibly the activation of systemic innate immune responses [5]. Type II resistance is controlled by resistance quantitative trait loci (QTL)/genes and is therefore more widely utilized and studied. Hundreds of Type II resistance QTLs have been reported across the wheat genome. Of these QTLs, a major QTL *Qfhb1* (syn. Qfhs.ndsu.3BS, *fhb1*), which is on chromosome arm 3BS, has been proven to be the strongest and most reliable, and is responsible for up to 60% of the variations in FHB resistance under various genetic backgrounds and environments [6,7,8,9,10,11,12]. Earlier reports have indicated that breeding FHB-resistant cultivars is challenging due to the limited understanding of the wheat resistance mechanisms in response to *F. graminearum* infection [9,13,14]. Therefore, this QTL has been the focus of most FHB-molecular research with a special focus on determining its mechanism and role in Type II FHB resistance.

*Qfhb1* was first identified in Sumai 3-derived mapping populations [7,9,10]. Fine mapping and sequencing efforts have localized it to an ~1 Mb interval between molecular markers sts3B-32 and sts3B-206 [12,15,16,17], where up to 28 genes have been reported by different research groups [17,18,19,20,21,22]. Several potential resistance mechanisms have also been proposed, but none has been validated without contradictions. For example, Lemmens et al. reported that *Qfhb1* was genetically associated with a strong ability to transfer DON into nontoxic DON-3-*O*-gulucoside [23], but the metabolic and proteomic studies of *Qfhb1* by Gunnaiah et al. did not find evidence to support this claim [24]. Gunnaiah et al. instead proposed that *Qfhb1* could mainly function in increasing the activities of the phenylpropanoid pathway in response to pathogen infections which lead to secondary cell wall thickening in rachises [24]. Hofstad et al. agreed that the rachis node is a key site of Type II resistance but believed that DON detoxification in infected rachis nodes could be the main resistance factor [18]. A gene of unknown biological function from a Chinese wheat cultivar Sumai 3, WFhb1_c1 (Wheat Fhb1 candidate 1), was shown to be associated with *Qfhb1* by eQTL and physical mapping [22]. The EST that led to the discovery of WFhb1_c1 is weakly similar to an Arabidopsis pectin methyl esterase inhibitor gene by sequence similarity and is down-regulated by pathogen infection in susceptible wheat genotypes [22,25]. The research group functionally suggested the jasmonate and ethylene signaling role of WFhb1_c1 in FHB resistance [26], but later the cloned gene sequence was found to be located outside of the *Qfhb1* marker interval [17]. Li and Yen [26] and Xiao et al. [21] suggested that the FHB resistance provided by *Qfhb1* is probably mediated by the jasmonic acid signaling pathway. However, none of the genes responsible for the above-mentioned activities have been found among the genes reported to be hosted in the QTL [16,17,20]. Recently, Rawat et al. claimed that a pore-forming toxin-like (PFT) gene is the genic component of *Qfhb1* [20]. However, Schweiger et al. did not observe any anti-fungal activity of this *PFT* in their yeast-based assay or the pathogen-dependent expression of this gene in their transcriptome analysis [17]. Hence, they proposed that a GDSL lipase gene (*GDSL*) is the most likely candidate gene of *Qfhb1*, since it was the one of many genes in this QTL that showed a significant increase in expression after pathogen infection in fhb1+ NIL. However, the authors could not rule out the candidacy of many other genes. GDSL lipase 1 has been reported to modulate system immunity via the ethylene (ET) signaling pathway in Arabidopsis [27]. Li and Yen also suggested the ET signaling pathway is involved in mediating FHB resistance in wheat [26]. However, the *GDSL* that was reported by Schweiger et al. [17] in the Sumai 3-derived FHB-resistant cultivar CM-82036 was not among the 13 genes identified in the *Qfhb1* interval of Sumai 3 by Pumphrey [19] and Rawat et al. [20]. Furthermore, this *GDSL* does not share sequence similarity with the Arabidopsis lipase-I gene. The unique existence of *GDSL* and *PFT* in *Qfhb1*-carrying wheat genotypes was one of the main reasons they were identified as genic components of *Qfhb1* [17,20]. Both Schweiger et al. [17] and Rawat et al. [20] admitted that at least one other gene within or nearby the sequenced QTL interval might also contribute to the observed FHB resistance presented by this QTL. Recombination within this QTL interval is also rare [16,17], which allows the co-segregation of multiple functional genic components of this dominant QTL.

Proteomics provides a unique gateway to the global examination of differentially accumulated proteins (DAPs) in plant during its interactions with pathogen and could thus enrich our understanding of the complex interactions that occur in plant cells [28,29,30,31,32,33,34]. Proteomics also helps to address the bias caused by possible post-transcriptional events during transcriptomic analyses. The biochemical changes revealed by proteomic analysis are of great interest because they can provide insights into critical “switch points” of defense-related pathways that can be manipulated to engineer crop plants with improved resistance or immunity to pathogens [28]. Proteomics has been utilized to understand the resistance mechanism of *Fusarium* infections provided by *Qfhb1* [24,35,36,37] but a major shortcoming with using the 2D-electrophoresis (2DE) strategy is gel-to-gel variations; therefore, most often, the revealed changes in protein accumulation could not be validated by independent technologies such as real-time PCR or Western blotting [38]. Moreover, in proteomics reports, sometimes the discoveries are based upon a relatively small number of proteins, which bias the assessment of vital biological questions regarding FHB resistance. However, most issues related to the simple 2DE can be avoided by using two-dimensional difference in gel electrophoresis (2D-DIGE) [39,40]. 2D-DIGE is a protein separation technique with a low gel-to-gel variation. This is because the two contrasting samples are simultaneously run in a single gel. 2D-DIGE can also be coupled with high-throughput mass spectrometric tools, such as MALDI-TOF/TOF, for protein identification [41,42]. 2D-DIGE has helped to achieve major success in understanding the phytopathogenic interactions that contribute to pathogenesis-induced changes in *Medicago truncatula* and the disease resistance mechanisms of various fungi and oomycetes [28,41,43]. Moreover, a compatible host–pathogen interaction in downy mildew between the host *Pisum sativum* and the pathogen *Peronospora vicia* was elucidated with the application of 2D-DIGE combined with ESI-MS/MS. In addition, 2D-DIGE was used to investigate xylanase inhibitor isoforms in response to infection of wheat with the *F. graminearum* ΔTri5 mutant [44].

By utilizing 2D-DIGE and MALDI-MS/MS technology, we studied proteomic changes during early FHB pathogenesis in the spikelet of a pair of near-isogenic lines (NIL), one that carries *Qfhb1* and one that does not carry *Qfhb1*. Here, we report the proteomic changes that were observed between the two contrasting NILs before and after they were challenged with *F. graminearum* and between the *F. graminearum*-challenged and the mock-inoculation samples of each NIL. We also discuss the implications of the observed proteomic changes to the molecular mechanisms of FHB susceptibility and the FHB resistance presented by *Qfhb1*.

## 2. Results and Discussion

The 2D-DIGE technology used in this study enabled us to simultaneously run two, contrasting protein samples on a single gel, eliminating the major random errors usually encountered in an ordinary 2DE assay, for which each sample is run on a separate gel. The NIL pair we used in this study was developed from a single F7 plant heterozygous for *Qfhb1*, as defined by the diagnostic markers gwm533, gwm133 and gwm493 and by contrasting disease phenotypes of resistance or susceptibility to FHB [45]. This NIL pair theoretically shares 99.2% of their genome and only differs due to genes carried on the gwm533–gwm493 interval of chromosome arm 3BS. Therefore, any differentially accumulated proteins between the NIL pair or the treatments within a NIL should be either encoded or controlled by genes located within this molecular marker interval and thus provide key information about the molecular mechanism of the FHB resistance presented by *Qfhb1*. In addition, the same NIL pair has been used in previous studies of *Qfhb1* that utilized transcriptomic analyses [18,22,25] and map-based cloning [12,16,19,20]; all these data should be comparable among the studies, but only to a certain extent, because the sample timings differ among studies. The combined consideration of the previous DNA and RNA analyses of these NILs and the current proteomics study enabled us to judiciously dissect the probable functional mechanism of *Qfhb1*.

### 2.1. Identification of DAPs in the NILs

**Figure 1:**
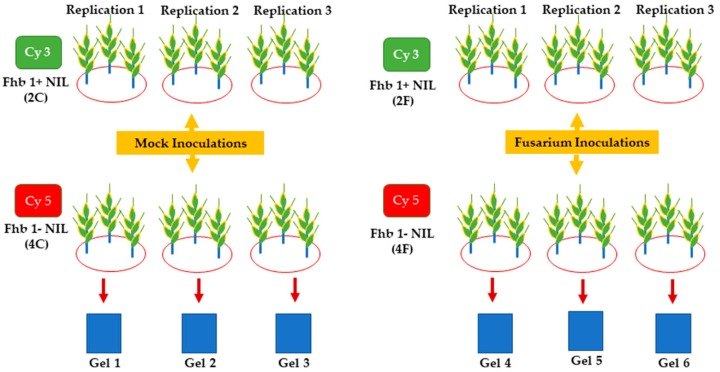
2D DIGE workflow. The two wheat NILs were grown in 12 pots (six pots with the fhb1+ NIL and six pots with the fhb1- NIL). Mock inoculated samples: The fhb1+ NIL (2C) was labeled with Cy3 dye and the fhb1- NIL (4C) was labeled with Cy5 dye. *Fusarium* inoculated samples: The fhb1+ NIL (2F) was labeled with Cy3 dye and the Fhb1- NIL (4F) was labeled with Cy5 dye.

To discover DAPs, the total protein from the *Fusarium*-inoculated and mock-inoculated samples was subjected to 2D-DIGE (Figure 1).

**Figure 2:**
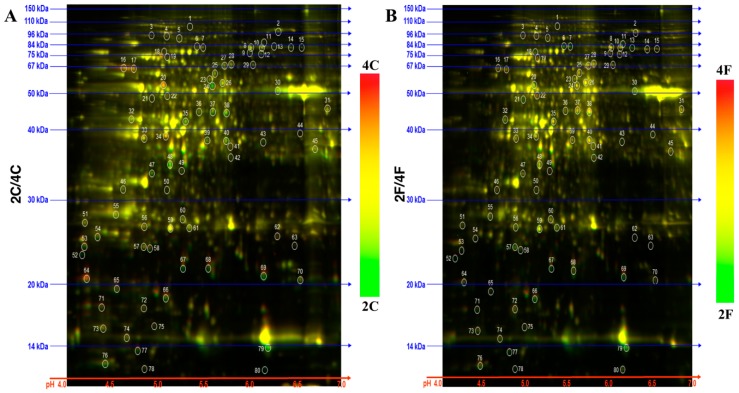
Representative 2D-DIGE gel image of the 2C/4C and 2F/4F samples. (**A**) Protein samples from the fhb1+ NIL (2C) and the fhb1- NIL (4C) mock-inoculation spikelet, which were minimally labeled with Cy3 (green) and Cy5 (red), respectively, and mixed in equal ratios; the total proteins were focused on a 13 cm IPG strip, pH 4.0-7.0, and then resolved on a 12% SDS polyacrylamide gel. The isoelectric points (pI) and molecular mass (kDa) values are noted. (**B**) The same protocol as above was followed with the *Fusarium*-inoculated spikelet of the fhb1+ NIL (2F) and the fhb1- NIL (4F). The yellow color of the spots indicates that the abundance of the protein was statistically similar between the two samples.

**Figure 3:**
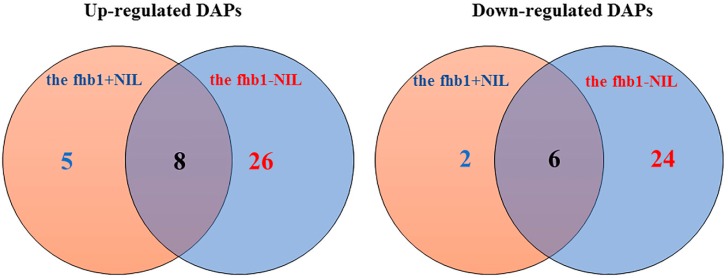
VENN diagram shows the distribution of significant DAPs (fold change ≥ 1.5, *p* ≤ 0.05) revealed by 2D-DIGE after 24 h of *Fusarium*-inoculation in the fhb1+ NIL and the fhb1- NIL.

The in-gel comparisons revealed DAPs for each NIL (Figure 2) at 24 h after inoculation (hai). We studied the 80 top-ranking DAPs and found that 50 were specific to the fhb1- NIL, seven were specific to the fhb1+ NIL, 14 were shared by both NILs, and the rest were not significant in either NIL (Table S2). Figure 3 shows the distribution of the significant DAPs in VENN diagram.

The gel image of each sample was then used to make comparisons between the two NILs (Figure S1A,B). These comparisons revealed constitutive DAPs in the absence of *Qfhb1* and more FHB-induced DAPs between the NILs, providing us the opportunity to look deeper inside the functional mechanism of *Qfhb1*. The four comparisons revealed an additional 12 down-regulated and six up-regulated DAPs in the fhb1- NIL (Table S2). Spots of these 80 DAPs were extracted from the gels (Figure 2 and Figures S2–S4), identified with MALDI-MS/MS and annotated for their potential functions (Table S3). Of them, 72 passed the confidence test for annotation with high confidence (protein score ≥ 72), two were of low confidence (protein score ≥ 31 but ≤71 and with a satisfied total ion score) and six were of no confidence (protein score ≤ 30) (Table S3). Sixty-nine DAPs were annotated with a known or predicted function, whereas the remaining 11 had either an unknown function or a known function of less or no confidence. Table S3 lists the Top 10 annotations for each DAP together with their protein score, total ion score and peptide counts for each annotation.

We then randomly selected seven proteins to determine their transcriptional-level expression changes in each NIL before and after *Fusarium* inoculation by RT-qPCR (Table S1). For the seven DAPs we tested, the fold changes in expression were either similar or non-significant (fold change ≤ 2) at the two molecular levels (Figure S5). The results at the transcript and protein levels agreed with each other or were at least not in disagreement. Therefore, our 2D-DIGE results were validated by an independent technique.

### 2.2. DAPs discovered in the fhb1+ NIL and the fhb1- NIL 

Table 1 shows the significant DAPs discovered from the comparisons we made within and between the NILs. The two in-gel comparisons revealed DAPs induced by the pathogen inoculation in each NIL, the comparison between the two mock-inoculated samples revealed constitutive DAPs between the NILs in the absence of pathogen infection, and the comparison between the two *Fusarium*-inoculated samples revealed important DAPs between the NILs challenged by the pathogen inoculations. Figure S6 illustrates the classification of the 71 DAPs based on their Gene Ontology annotations. In the sections below, we focus our discussion on these DAPs since they provide the important information about the proteins that contribute to FHB resistance or susceptibility.

### 2.3. Constitutive DAPs in the Absence of Qfhb1

Fourteen DAPs were found to be constitutive (Proteins #: 1, 12, 16, 17, 20, 24, 47, 52, 62, 64, 67, 69, 79, and 80). Of these, five were up-regulated (Proteins #: 16, 17, 20, 62, and 64) and nine were down-regulated (Proteins #: 1, 12, 24, 47, 52, 67, 69, 79, and 80) in the fhb1- NIL compared to those in the fhb1+ NIL. The pathogen inoculation up-regulated six of the nine down-regulated DAPs and suppressed all five up-regulated DAPs and three down-regulated DAPs (Table 1, Categories 1 and 2 and Figure 3). Proteins #11, #14, #19, #34, #35, #37 and #38 could also be included in this group since they changed by 1.43~1.49-fold, which is not too distant from the 1.5-fold threshold (Table 1). Because this observation was prior to pathogen inoculations, these DAPs resulted from the presence/absence of *Qfhb1*. In other words, in a healthy plant, *Qfhb1* promotes the expression of the nine down-regulated DAPs but suppresses the expression of the five up-regulated DAPs in this group. These DAPs most likely represent the basal difference between the NILs upon which the FHB resistance presented by *Qfhb1* is built, since all but Protein #80 were insignificant in the *Fusarium*-inoculated fhb1+ NIL. Of these 14 (or, 17 considering the lower threshold) DAPs, only three (or five) remained significant after pathogen inoculation; this indicates they are important for FHB resistance presented by *Qfhb1*.

Protein #80 is very interesting. First, its expression in the fhb1- NIL was constitutively lower by approximately seven-fold relative to its expression in the fhb1+ NIL, but it was remarkably up-regulated by approximately 20-fold in response to *Fusarium* inoculation. Second, it was the only protein of the 14 DAPs that had an approximately 3.5-fold increase in abundance in the fhb1+ NIL after pathogen inoculation. However, it demonstrated no significant change between the two *Fusarium*-inoculated samples. This indicates that the observed spike in this protein’s abundance after pathogen inoculation could be due to genes homoeologous to the gene controlled by the studied QTL. Therefore, Protein #80 is not important to the FHB resistance conferred by *Qfhb1*. Currently, we do not know the exact biological function of Protein #80 because it is poorly annotated due to its poor ionization during mass spectrometry. However, the approximately 20-fold increase in abundance of this protein in the fhb1- NIL after pathogen inoculation suggests that this protein may play a very important role in FHB susceptibility and thus warrants further study.

Protein #1 is very weakly similar to glycosyltransferase (protein score = 32), leucyl-tRNA synthetase (protein score = 31) and Pm3 (protein score = 23) (Table S3). Genes encoding the first two proteins were also found in the *Qfhb1* marker interval by DNA and mRNA sequencing [17]. Our data show that Protein #1 significantly decreased in abundance in the absence of *Qfhb1*. We also found that the pathogen inoculation seemed to induce the expression of homoeologous genes to that of Protein #1 to compensate for its decreased expression in the fhb1- NIL due to the absence of *Qfhb1*. Therefore, Protein #1 may not participate in the FHB resistance conferred by *Qfhb1*.

Protein #52 is a vacuolar invertase, and vacuolar invertases hydrolyze sucrose into glucose and fructose. Fructose has been proposed to be involved in the abscisic acid (ABA)/ethylene (ET) signaling pathway in resistance to biotrophic pathogens [46,47]. Our data show that the expression of Protein #52 in fhb1- NIL significantly increased in response to *F. graminearum* inoculations, which, in turn, could significantly reduce the availability of sucrose. It has been reported that reductions in sucrose availability enhance susceptibility to the hemibiotrophic pathogen *Colletotrichum higginsianum* in Arabidopsis [48]. *F. graminearum* is also considered as a hemibiotrophic pathogen [49]. Therefore, the enhanced susceptibility to FHB in the fhb1- NIL could be due to the enhanced involvement of fructose in the resistance mediated by ABA/ET signaling. However, no significant difference was observed in the abundance of Protein #52 between the NILs after pathogen inoculation, which indicates its insignificance in the function of *Qfhb1*.

Proteins #67, #69 and #79 were the only three proteins in this group whose expression patterns were not changed by pathogen inoculation, and we can add Proteins #11 and #19 if the lower threshold is adopted (Table 1 and Table S1). In fact, these proteins were down-regulated in the fhb1- NIL before and after pathogen inoculations, whereas their expression changes after inoculation were not significant in the NILs, except for that of Protein #11, which was up-regulated by approximately two-fold in both NILs. Protein #69 is a chloroplastic cytochrome b6-f complex iron-sulfur subunit and Protein #79 is a ribulose bisphosphate carboxylase small subunit. They both are members of the photosynthetic pathway. Protein #67 is a eukaryotic translation initiation factor 5A1. Protein #11 is probably a 5-methyltetrahydropteroyl triglutamatehomocysteine methyltransferase-like protein, which is involved in amino acid biosynthesis. Protein #19 could be a starch synthase. Therefore, it seems that the absence of *Qfhb1* in the fhb1- NIL significantly weakens its photosynthesis capability and to synthesize new proteins; but could result in producing more starch, making the NIL vulnerable to attacks by *F. graminearum*.

Five DAPs (Proteins #16, #17, #20, #62 and #64) were constitutively up-regulated in the fhb1- NIL, suggesting that they might have suppressed by the presence of *Qfhb1* in the fhb1+ NIL. However, these DAPs were also suppressed by pathogen inoculation. Protein #16 functions in the formation and breakage of disulfide bonds between cysteine residues within proteins as they fold; therefore, it acts to catalyze protein folding. Protein #62 is a glutathione transferase F5, a member of the glutathione S-transferase (GST) superfamily [50]. A common GST ability is to catalyze the conjugation of the reduced form of glutathione (GSH) to xenobiotic substrates for the purpose of detoxification [51]. Pathogen infection usually rapidly induces oxidative burst and the accumulation of GSH, triggering calcium release [52]. GSH then interacts with H_2_O_2_ and calcium to transmit stress signals down the signaling pathways [53]. Increasing GSH activities after pathogen infection is a known hypersensitive response (HSR) resistance mechanism of by pathogen-infected plants [54]. Therefore, the lower abundance of glutathione transferase F5 in the fhb1- NIL than the fhb1+ NIL after pathogen inoculation resulted in a higher GSH accumulation and, consequently, a stronger HSR response to *F. graminearum* inoculation by the NIL. In other words, the observed higher abundance of Protein #62 in the fhb1+ NIL could indicate that the FHB resistance conferred by *Qfhb1* is due at least in part to the ability of Protein #62 to increase the detoxification of GSH and thus reduce the HSR.

Protein #17 was up-regulated in the absence of *Qfhb1*, but down regulated by pathogen inoculation (Table 1). Protein #17 is a protein phosphate 2A (PP2A) structural subunit. PP2A is a major serine/threonine protein phosphatase that plays key roles in regulating cellular signaling within the plant immunity signaling network underlying resistance against biotrophic pathogens [55]. PP2A controls ABA, ET and SA signaling and many metabolic pathways in cell. For example, PP2A decreases the production of radical oxygen species (ROS) in the cell and thus reduces salicylic acid-dependent resistance responses to pathogen infection, which triggers HSR [55]. The observed 1.47-fold down-regulation of PP2A by pathogen inoculation in the fhb1- NIL indicates a possible higher ROS and hence stronger HSR in the absence of *Qfhb1*.

Protein #20 is a phosphoethanolamine methyltransferase (PMT). PMT catalyzes the three-step methylation of phosphoethanolamine to form phosphocholine, a critical step in the synthesis of phosphatidylcholine, a major phospholipid constituent of cell membranes. Like other pathogens, *F. graminearum* infection damages the host cell membranes during its infection. Repairing the damaged membrane is a known resistance response by plant cells. Interestingly, pathogen infection significantly suppressed PMT expression only in the fhb1- NIL. Higher PMT abundance in the fhb1+ NIL means stronger membrane repairing capability. Therefore, part of the biological function of *Qfhb1* is probably strengthening cell membranes during FHB pathogenesis. Interestingly, a gene encoding a methyltransferase domain-containing protein was found in the *Qfhb1* marker interval and considered as a candidate for the genic components of *Qfhb1* by Schweiger et al. [17]. They also observed constitutive expression of this gene and predicted its role in resistance to trichothecene toxins. If this protein indeed could detoxify trichothecenes, the approximately two-fold decrease in the accumulation of the protein product of this gene in the fhb1- NIL after pathogen inoculation could suggest a possible pathogen suppression role of the host anti-trichothecene activities.

Protein #64 is a salt tolerance (STO) protein. STOs belong to B-box type Zn-finger proteins that bind to the myeloblastosis (MYB) transcription factor and regulate MYB functions [56]. Interestingly, Protein #65, an MYB transcription factor, was also down-regulated in the fhb1- NIL. One of the known biological functions of STOs is to sense and negatively regulate oxidative stress [57] and a wide range of stress-related downstream genes in the cell [58]. Therefore, a lower STO abundance in the fhb1- NIL means a weaker capability to detect and reduce oxidative and other stresses in the NIL during the pathogenesis, and, again an inability to reduce the HSR caused by such stresses.

### 2.4. Fusarium-Induced DAPs Common in Both NILs

Thirteen DAPs (seven up- and six down-regulated) were found to behave similarly in both NILs in response to pathogen inoculation (Table 1, Categories 5 and 6). Another three up-regulated DAPs, Proteins #3, #13 and #42, could also be included in this group since their abundances exhibited a 1.48–1.49-fold change. Therefore, these DAPs either contribute to or result from general resistance responses in the plant, which are probably not under the control of *Qfhb1*. Protein #11, which could also be a constitutive DAP, was the only one in this group that was significantly less abundant in the fhb1- NIL than in the fhb1+ NIL after pathogen inoculation. This difference is probably due to its constitutive down regulation in the absence of *Qfhb1*. As we discussed before, this protein is involved in amino acid biosynthesis. Less abundance of Protein #11 in the fhb1- NIL after pathogen inoculation means less protein production.

Four DAPs in this group are proteins that were involved in photosynthesis. Proteins #14 and #15 are the large subunit of ribulose-1,5-bisphosphate carboxylase/oxygenase (RuBisCO), which catalyzes the first step of carbon fixation during photosynthesis. Proteins #55 and #58 are chlorophyll a/b binding protein (LHCB), which serves as antenna during the light harvesting process. The observed down-regulation of LHCB in both NILs represents the damage to photosynthetic pathway by the pathogen inoculation, and the increase in RuBisCO expression seemed to be a general defense reaction to compensate for damage. Another two DAPs, Proteins #51 and #57, that were specific to the fhb1- NIL are also LHCBs. LHCBs are also known to modulate ROS homeostasis in the chloroplast and participate in ABA signaling in guard cells [59]. Therefore, the observed down-regulation of these LHCBs could mean less ROS homeostasis in the chloroplasts after pathogen inoculation, particularly in the fhb1- NIL, leading to a higher ROS concentration and chloroplast damage. Since *F. graminearum* is known to infect plants through stomata, reducing the abundance of LHCBs in the fhb1- NIL could also reduce the responsiveness of stomatal movement to ABA, which keeps stomata open, facilitating pathogen infection. The observed approximately one-fold up-regulation of Protein #57, although not significant, could mean that *Qfhb1* helps reduce the negative impact caused by the pathogen-induced down-regulation of LHCBs.

The abundance of Protein #72, a pathogenesis-related protein 10 (PR-10), significantly increased by approximately two-fold in both NILs after the pathogen inoculation (Table 1). PR-10 is known to be part of systemic acquired resistance, activating other stress-related proteins in cells challenged by pathogen infection leading to HSR [60,61,62]. Using a computational approach, we identified one of the closest homologs of wheat PR-10 in *Zea mays* (UniProt ID: B6SQM6), which showed 33% identity and a query cover of 95%. We searched for a variety of functional protein–protein interactions using the STRING database with *Zea mays* PR-10 fixed as a central regulatory component of the network to obtain an improved understanding of the wide range of defense-related regulatory interactions (Figure S7). From this network, we identified the following key proteins associated with PR-10: (1) terpene synthase 6 (UniProt ID: K7TGS7), which is involved in the biosynthesis of the bicyclic sesquiterpene; (2) zeamatin precursor (UniProt ID: B6TL22), which possesses antifungal activity; (3) pathogenesis-related protein-4 (UniProt ID: B4FVP5), which is a chitinase; (4) a putative uncharacterized protein (UniProt ID: B6SY34); (5) a putative serine-type endopeptidase inhibitor (UniProt ID: A3FMA3); (6) glucan endo-1,3-beta-glucosidase (UniProt ID: B8A260); (7) cytochrome b5 (UniProt ID: B6SLW8); and (8) a polygalacturonase inhibitor (UniProt ID: B6SN21). This network of nine proteins is involved in 34 protein–protein interactions (Figure S7). Apparently, this network contributes to HSR. Two proteins in this network have attracted our attention: the terpene synthase and polygalacturonase inhibitor. Genes encoding terpene synthase and polygalacturonase have been found in the *Qfhb1* marker interval by Schweiger et al. [17] and Pumphrey [19]. However, Pumphrey excluded the candidacy of these genes as genic components of *Qfhb1* because the over-expression of these genes did not increase FHB resistance [19], and our observation of an approximately two-fold induction of Protein #72 by pathogen inoculation in both the NILs supports this conclusion.

Protein #53 is a highly conserved, translationally-controlled tumor protein (TCTP) in many eukaryotes. TCTP is involved in a variety of cellular anti-stress activities including stabilizing microtubules [63] and binding to calcium to maintain cell health and inhibit apoptosis [64]. A calcium-binding protein gene has previously been found in the *Qfhb1* marker interval [18,19,21,65] but was excluded as a genic component of *Qfhb1* because its overexpression in susceptible wheat did not improve FHB resistance [19] and because it was found to express in FHB-susceptible genotypes [18]. Su et al., however, believed that this calcium binding protein gene is a candidate genic component of *Qfhb1* because a deletion in its promoter region causes its silencing in FHB-resistant genotypes [65]. The down-regulation of Protein #53 could cause microtubules to become less stable, which was further evidenced by the observed down-regulation of alpha tubulin-2A (Protein #21) in our study.

Protein #65 is a member of the MYB family of transcription factors. This protein could play an important role in stress tolerance in plants, probably via the regulation of phenylpropanoid metabolism [66]. The approximately two-fold down regulation of Protein #65 in both NILs could mean a weaker stress tolerance after inoculation. Hofstad et al. revealed an up-regulated MYB transcriptional factor in the fhb1+ NIL, but the up-regulation was 96 h after inoculation [18]. Other DAPs in this group include up-regulated Protein #50, which may possess RNase activity [67], Proteins #35 and #76, which are cytoskeleton components, and two down-regulated DAPs (Proteins #33 and #54) with unknown functions.

### 2.5. Fusarium-Induced DAPs That Were Specific to the fhb1+ NIL

Of the 80 DAPs, only seven were found to be specific to the fhb1+ NIL; of these five were up-regulated and two were down-regulated. This group only has six members if Protein #13 is counted as a shared protein (Table 1, Categories 7 and 8). These DAPs could contribute to the FHB resistance presented by *Qfhb1*.

Protein #7 is a eukaryotic translation initiation factor 3 subunit D-like protein and was up-regulated by approximately two-fold in the fhb1+ NIL. Another eukaryotic translation initiation factor (Protein #68) was found to be down-regulated by 1.66-fold in the fhb1- NIL. Therefore, there was much higher translation initiation in the fhb1+ NIL than in the fhb1- NIL. Schweiger et al. [17] found a similar gene in the *Qfhb1* marker interval and proposed that it might contribute to easing the toxification of ribosomes by DON. Our observations suggest there is a pathogen-induced reduction of such detoxification efforts in the fhb1- NIL, and *Qfhb1* could decrease these reductions by significantly increasing translation activities.

Protein #18 is a glucose-1-phosphate adenylyltransferase (GPA) large subunit protein, which is involved in starch synthesis. GPA participates in sucrose metabolism by catalyzing the production of ADP-glucose in the starch synthesis pathway. Sucrose can be a signaling molecule in plants that regulates several transcriptional factors, including bZIP and MYB [68,69,70]. Sucrose-responsive elements (SURE-boxes), such as A- and B-boxes, the TGGACGG element and SP8 motifs, are known conserved cis elements in the promoters of many genes [71,72], which function as master regulators of many central developmental and physiological processes, including abiotic and biotic stress responses [69]. Sucrose also plays a synergistic role with ABA. Therefore, Protein #18 could work with other DAPs regulating sucrose homeostasis in the infected cells.

Proteins #49 and #70 are two proteins in this group that were down-regulated. Protein #49 could be a root phototropism 2 (RPT2) protein (protein score = 70), a cysteine-rich receptor-like protein kinase (CRK) 29-like protein (protein score = 56) or a RING finger protein (protein score = 54) (Table S3). In Arabidopsis, CRKs are synthesized upon pathogen perception and coordinately act to enhance plant immune responses [73]. CRKs could also be involved in directly sensing ROS via redox regulation by their extracellular protein domain [74]. The suppression of CRK synthesis in the fhb1+ NIL by pathogen infection could reduce ROS signaling and the plant immune response, which could be a possible FHB resistance mechanism presented by *Qfhb1*. The RING finger protein plays a key role in the ubiquitination pathway, which, in turn, targets the substrate protein for degradation [75,76], impacting gene transcription, translation, mRNA trafficking, cytoskeleton organization, epithelial development, cell adhesion, protein folding, chromatin remodeling and zinc sensing [77]. Schweiger et al. found a RING finger protein gene in the QTL interval that showed a low expression in both NILs, so it was excluded as a candidate genic component of *Qfhb1* [17].

Protein #70 is an abscisic stress ripening (ASR) protein regulated by ABA. ASRs are a group of plant-specific proteins produced in response to abiotic stresses with an undefined biological function that are potentially involved in an ABA-mediated pathway [78,79]. A wheat ASR was reported to confer drought stress tolerance via the regulation of ROS homeostasis [80]; a rice ARS functions as an effective ROS scavenger [81]. The observed significant down-regulation of Protein #70 in our study indicates a significant reduction in ROS scavenging in the fhb1+ NIL after *Fusarium* inoculation. Interestingly, Protein #47 is also an ASR protein. Comparing to its abundance in the fhb1+ NIL, Protein #47 was down-regulated approximately 1.86-fold and 1.14-fold in the fhb1- NIL before and after *Fusarium* inoculation, respectively. Therefore, our observations suggest that a possible role of *Qfhb1* is to reduce anti-stress activities by increasing ROS scavenging.

Proteins #6 and #44 are two up-regulated DAPs in this group with unknown function. Comparatively, these two DAPs had a lower abundance in the fhb1- NIL than in the fhb1+ NIL. We previously reported a functional component of *Qfhb1*, WFhb1_c1, with an unknown function [22]. WFhb1_c1 was also down-regulated only in the fhb1- NIL. Further study of these two DAPs should reveal more information about the pathway of *Qfhb1*-mediated FHB resistance.

However, a similar trend in terms of changes, although not significant in this study, was observed in the fhb1- NIL. Proteins #6, #7 and #13 were significantly less abundant in the fhb1- NIL than in the fhb1+ NIL after pathogen inoculation. Protein #13 could be a mechanosensitive ion channel membrane protein (protein score = 30) capable of responding to mechanical stress, a pentatricopeptide repeat domain-containing protein (protein score = 27) that regulates biogenesis of chloroplasts and mitochondria [82], or a serine/threonine protein kinase (protein score = 26), that could play a central signaling role in disease resistance (Table S3).

### 2.6. Fusarium-Induced DAPs specific to the fhb1- NIL

Thirty-seven DAPs generally exhibited a similar trend of change in both the NILs after pathogen inoculation, but the changes were significant only in the fhb1- NIL (Table 1, Categories # 3, 4, and 9,). Of the 37 DAPs, Proteins #57 (chlorophyll a-b binding protein, LHCB) and #71 (peroxiredoxin-2E-2, Prx2) were the only two that showed a significant difference in accumulation between the two *Fusarium*-inoculated samples, and both were down-regulated in the fhb1- NIL. This may indicate that the photosynthesis and redox are the two pathways most impacted by pathogen infection. In addition to Protein #57, 10 more DAPs in this group are involved in photosynthesis. We have already discussed the four shared DAPs (two up-regulated and two down-regulated) that are involved in photosynthesis. Therefore, *F. graminearum* infection in general could result in reduced photosynthesis in wheat spikes in absence of *Qfhb1*, since 12 of the 15 photosynthesis-related DAPs were down-regulated after inoculation. Five of the DAPs were LHCB proteins; all were down-regulated, while only one of the five RuBisCO proteins were down-regulated. This observation suggests that it is the light-harvesting mechanism, not the carbon fixation mechanism, that is most impacted by *Fusarium* infections. Other photosynthesis elements negatively impacted by the pathogen inoculation include a M-type thioredoxin (Protein #73), which is an essential redox regulator for light regulation in plants, a 15 kDa thylakoid lumenal protein 1 (Protein #77), which provides the environment for oxygen evolution, a plastocyanin-mediated electron transfer and photoprotection protein; an adenylate kinase (ADK, Protein #56), which catalyzes the inter-conversion of adenine nucleotides and plays an important role in cellular energy homeostasis, and a cytochrome b6-f complex iron-sulfur subunit (Protein #69), which catalyzes the transfer of electrons from plastoquinol to plastocyanin. During photosynthesis, the cytochrome b6-f complex is one step in the chain of reactions that transfer electrons from photosystem II to photosystem I, pumping protons into the thylakoid space and creating an electrochemical (energy) gradient that is later used to create ATP. Protein #61 is oxygen-evolving enhancer protein 2, required for high levels of photosynthetic oxygen evolution. Our observations suggest that almost all of the light reactions steps in photosynthesis are negatively impacted by pathogen inoculation.

Protein #71 (Prx2) is an antioxidant enzyme that reduces hydrogen peroxide and alkyl hydroperoxides. Proteins #43 (stearoyl-ACP desaturase, SAD) and #66 (thioredoxin-dependent peroxidase, TD-Prx) are two other DAPs in this group that are involved in anti-ROS activities. SAD plays a role in drought and hypoxia stress signaling in Arabidopsis crown galls by increasing the levels of unsaturated fatty acids under hypoxia and drought stress conditions [83]. TD-Prxs functions as an antioxidant agent toward ROS, protecting DNA against ROS-induced degradation [84]. The observed down-regulation of these three proteins indicates that there was a significant reduction in anti-ROS activities in the fhb1- NIL but not in the fhb1+ NIL after pathogen inoculation. This implies that *Qfhb1* functions to alleviate this reduction.

Proteins #9 and #12 are a phenylalanine ammonia-lyase (PAL), Proteins #36, #37 and #38 are S-adenosylmethionine synthases (SAMS), and Proteins #39 and #40 are caffeic acid 3-O-methyltransferases (COMTs). These seven up-regulated DAPs are all involved in lignin biosynthesis, and they all increased in abundance after pathogen inoculation by approximately two-fold in the fhb1- NIL, an increase that was approximately 1-fold higher than that in the fhb1+ NIL. Lignin are important components of plant cell walls. Increasing lignin biosynthesis and mobilizing more lignin to the wounding site constitute a known general resistance response against mechanical damage. Several other cell skeleton components, such as alpha tubulin-2A (Proteins #21 and #22), actin (Protein #35) and profilin (Protein #76), were also increased in abundance in both NILs after pathogen inoculation. Clearly, both NILs increased the repair of cell structures damaged by the pathogen, and the increase was much greater in the fhb1- NIL than the fhb1+ NIL. More importantly, cells use cinnamate made by PAL to produce not only lignin but also salicylic acid (SA) [85]. Therefore, the up-regulation of PAL could also enhance SA-signaling, resulting in HSR. SAM is also an ET precursor [86]. Increased SAM accumulation in the fhb1- NIL could also mean more ET production and thus enhanced ET-signaling. The role of ET in FHB resistance/susceptibility is complicated. While the ET-signaling pathway works synergistically with JA in mediating FHB resistance [26], *F. graminearum* can also exploit ET signaling in the host cells for its colonization [87]. Moreover, Ding et al. observed the association of ET synthesis with FHB susceptibility in their mutant study [49]. The increased production of ET in this case could result in stronger signals for the pathogen to colonize the host, leading to increased susceptibility.

Protein #26 is ATP synthase subunit 1, which is a key enzyme in mitochondria for ATP production and is regulated by cellular energy demands [88]. ATP synthase uses the energy from a proton gradient to produce ATP. Protein #78 is vacuolar ATPase (V-ATPase) subunit F, which, together with subunit D, forms the central rotor axle of V-ATPase [89]. V-ATPase uses the energy from ATP hydrolysis to produce a proton gradient. Therefore, the two enzymes work in opposite ways. Our data show that pathogen inoculation induced an approximately 1.74-fold reduction in the V-ATPase abundance, which could reduce the ATP consumption and proton gradient, and stimulate a 1.65-fold increase in the ATPase abundance, making more ATP and further reducing the proton gradient. Hence, *Fusarium* infection could cause a significantly lower proton gradient across the relevant membrane in the fhb1- NIL, which drastically may alter the intracellular environment and disrupts the balance of the redox state. For example, adequate cyclic electron flow is essential for photosynthetic machinery to work properly and for plants to tolerate abiotic stresses [90]. Altering the proton gradient across thylakoids will certainly disturb adequate cyclic electron flow and thus upset photosynthesis. Unbalanced proton gradients could also alter the intracellular pH environment, negatively impacting many biochemical reactions and leading to unhealthy cells or even death. A gene encoding an NB-ARC-containing protein is in the *Qfhb1* marker interval [17,20]. NB-ARC is a functional ATPase domain and functions as a molecular switch in R proteins [91,92]. However, both groups excluded this gene as a functional candidate of the QTL because it either lack changes in expression in resistant and susceptible wheat [20] or it was not expressed [17].

Protein #34 is a glutamine synthetase (GS) that plays a key role in the maintenance of redox balance in chloroplasts. GS assimilates N from NO_3_^−^ and NH_4_^+^ into the plant central metabolism via the GS and glutamine-oxoglutarate aminotransferase (GS/GOGAT) cycle [93]. Glutamate is the precursor to GSH synthesis. GSH participates in the ascorbate/GSH cycle and is known to play a major role in the antioxidant defense against biotrophic pathogens [94]. Necrotrophic pathogens are known to be able to exploit GSH content to induce host susceptibility [95,96]. Decreased GS activity is known to prolong the availability of glutamic acid, causing excitotoxicity and thus cellular damage in humans [97]. Similar to ROS, nitric oxide (NO) is a key player in HSR [98], and NO is known to target GS for its post-translational inactivation [99]. As discussed earlier, glutathione transferase F5 (Protein #62), which conjugates the reduced form of GSH to xenobiotic substrates, is also significantly down-regulated by pathogen inoculation in the fhb1- NIL, possibly resulting in a higher accumulation of GSH. At the same time, reduced GS activity leads to increased glutamate availability for GSH synthesis. Therefore, pathogen infection induces the production of more GSH and the removal of less GSH in the fhb1- NIL, resulting in a higher abundance of GSH, which probably is an intermediate step for HSR that eventually leads to higher FHB susceptibility. GS is also involved in the remobilization of amino acids in cells because glutamate plays a pivotal role in amino acid metabolism. Other enzymes involved in amino acid synthesis, such as methionine synthase 1 (Protein #8), 5,10-methylene-tetrahydrofolate reductase (Protein #25) and 5-methyltetrahydropteroyltriglutamate (Protein #11), were also increased in both the NILs after *F. graminearum* inoculation, but the changes were only significant in the fhb1- NIL. This observation suggests that there is a much higher demand for amino acid synthesis in the susceptible wheat than in the resistant wheat. Therefore, the pathogen-induced reduction of GS could also make cells unable to meet the GS demands, leading to markedly reduced amino acid synthesis and thus cell damage or even death.

Our data show that starch synthase (Protein #19), phosphoglucomutase (PG, Proteins #27, #28 and #29), and vacuolar invertase (Protein #52) were all significantly up-regulated in the fhb1- NIL by pathogen inoculation. The amount of glucose-1-phosphate adenylyltransferase (GPA) large subunit (Protein #18) was also increased by 1.17-fold. These proteins are all involved in sucrose metabolism. During sucrose metabolism, vacuolar invertase breaks down sucrose into glucose and fructose. Glucose is then converted to glucose 6-phosphate by hexokinase. PG facilitates the inter-conversion of α-d-glucose-1-phosphate from glucose-6-phosphate. GPA catalyzes the production of ADP-glucose from α-d-glucose-1-phosphate and ATP, which could be made by the significantly up-regulated Protein #26. ADP-glucose is used by starch synthase to synthesize starch. A fructokinase (Protein #41), which also regulates starch synthesis [100] was also up-regulated by 1.32-fold (Table S2). Fructokinase transfers a phosphate group from ATP to fructose to form D-fructose 6-phosphate. The observed up-regulation of GPA, PG, starch synthase, fructokinase and vacuolar invertase could significantly increase the removal of sucrose in the cytosol, promoting ABA/ET signaling-mediated susceptibility to *F. graminearum* infection.

Protein #43 is a stearoyl-ACP desaturase (SACPD), which initiates multiple desaturations of fatty acids to make oleic acid. Song et al. compromised the powdery mildew resistance in an Arabidopsis ssi2 mutant by overexpressing the wheat SACPD gene *TaSSI2* in the mutant, indicating that resistance to biotrophic pathogens needs a low level of SACPD activity [101]. Therefore, the observed significant down-regulation of SACPD in the fhb1- NIL after pathogen inoculation suggests a significantly higher resistance response to biotrophic pathogens and thus significantly higher HSR in the NIL.

Comparing the *F. graminearum*-inoculated samples of the two NILs revealed 13 DAPs (Table 1). These 13 DAPs should be important in defining the FHB resistance presented by *Qfhb1*. Relative to the corresponding DAP levels in the fhb1+ NIL, 11 of the 13 DAPs were down-regulated in the fhb1- NIL. As discussed above, the down-regulated DAPs were involved in photosynthesis (Proteins #57, #69 and #79), protein production (Proteins #7, #11 and #67), and stress reduction (Proteins #43 and #71); one was a potential signaling molecule (Protein #13). Proteins #6 and #13 may play a pivotal role in FHB resistance since they both were significantly up regulated by pathogen inoculation in the fhb1+ NIL and behaved similarly to WFhb1_c1, the proposed functional genic component of *Qfhb1* reported by Zhuang et al. [22]. The observed increase in starch synthase (Protein #19) suggests more sucrose was used for amylose production, so less was used for sucrose signaling.

## 3. Materials and Methods 

### 3.1. Plant Materials and Fusarium Inoculation

Two wheat NILs 260-1-1-2 (the fhb1+ NIL) and 260-1-1-4 (the fhb1- NIL) were used in this study. These two NILs have an identical genome, except for the Xgwm493-Xgwm533 interval on chromosome arm 3BS, which hosts *Qfhb1* [19]. The NILs were produced and kindly provided by Dr. James A. Anderson of the University of Minnesota. The two NILs were grown in 12 pots (6 pots with the fhb1+ NIL and 6 pots with the fhb1- NIL) filled with MeteroMix 360 (SunGro Horticulture, Canada) in a Conviron (Winnipeg, Canada) growth chamber under 16/8 h light/dark photoperiod and 20/16 °C day/night temperature cycles with complete randomization. Each pot had 3 wheat plants (Figure 1). The light intensity at the plant level was approximately 1200 ± 20 μmoles m^−2^ s^−1^. A total of 10 g of controlled release fertilizer (Multicote Agri; Tessman Seeds, Sioux Falls, SD) was applied to each pot one week after planting. Watering was performed as needed with tap water to keep appropriate soil moisture during the experiment.

The Butte 86 strain of *F. graminearum* (accession number NRRL38661) was used for pathogen inoculations of the NILs. At the 60% anthesis stage, point inoculation was done on the two central spikelet pairs of each plant (main tiller) with 10 µL of freshly prepared spore suspension (1 × 10^5^ macroconidia mL^−1^ in 0.2% [vol/vol] Tween 20) or with sterilized water (mock control). Each inoculated spike was sealed in a clear plastic zip-lock^TM^ bag containing a water-saturated cotton ball to maintain humidity until sampling. Samples of the inoculated spikelet were collected at 24 h after inoculation (hai) to investigate the early responses of the NILs, as this time point is critical to FHB development, as revealed by our previous studies [22,25,26]. One pair of an inoculated and a mock spikelet from each plant were sampled as one pool for protein and RNA isolation, and one pair of spikelet were left on the plant to visually monitor the success of inoculation by disease symptom development. All samples were snap frozen in liquid nitrogen immediately after harvesting from the plant and stored at −80 °C prior to use in the next step.

### 3.2. Proteomic Analyses and Identification of Differential Abundant Proteins

Protein extractions were performed per the procedure of Hurkman and Tanaka [102]. Briefly, the spikelet tissue was ground in liquid nitrogen to a fine powder using a mortar and pestle. Afterwards, 400 mg of fine powder from each sample was homogenized individually in 3 mL of 2D cell lysis buffer (30 mM Tris-HCl, pH 8.8, 0.9 M sucrose, 10 mM EDTA, 7 M urea, 0.4% 2-mercaptoethanol, 2 M thiourea and 4% CHAPS) and an equal volume of Tris-buffered phenol pH 8.8, vortexed for 30 s and incubated for 30 min at 4 °C [103]. The mixtures were centrifuged at 3381× *g* for 15 min at 4 °C, and the phenolic phase was collected and precipitated overnight at −20 °C by adding 5 volumes of ice-cold 0.1 M ammonium acetate in 100% methanol. The next day, the protein pellet was washed in 5 mL of 0.1 M ammonium acetate in 100% methanol and centrifuged for 20 min at 9391× *g*, followed by a wash in 5 mL of ice-cold 80% acetone and a final wash in 4 mL of 70% ethanol. The protein pellet was air-dried and stored at −80 °C prior to the subsequent experiments. The protein concentration was measured using the Bradford reagent with BSA as the standard reference [102].

The 2D-DIGE assays were performed by Applied Biomics (Hayward, CA, USA) following the procedures reported in the previous proteomic evaluations by our group and in other successful stress proteomic studies [104,105,106,107,108]. Briefly, 30 µg of a minimal fluorescent-labeled protein mixture was used for the analytical gels in each 2D-DIGE experiment. Each mixture consisted of an equal amount of Cy3-labeled fhb1+ NIL protein sample and Cy5-labeled fhb1- NIL protein sample. Two-times-concentrated 2D sample buffer (Bio-Rad, Hercules, CA, USA) was added to each mixture, followed by 100 μL of DeStreak solution and rehydration buffer (Bio-Rad, Hercules, CA, USA), to obtain a final volume of 350 μL for the gradient (pH 4.0–7.0) IPG strips. The protein samples were loaded onto the IPG strips and isoelectric focusing (IEF) was performed as described previously [104,105,106,107,108,109]. Upon the completion of IEF, the IPG strips were incubated in freshly-made equilibration buffer-I for 15 min, after which they were incubated in equilibration buffer-II (Bio-Rad, Hercules, CA, USA) for another 15 min with gentle shaking. The IPG strips were rinsed in Tris-glycine-SDS running buffer (Bio-Rad, Hercules, CA, USA) and then transferred onto a 12% SDS polyacrylamide gel. The gel was then sealed with 0.5% agarose solution for the second-dimension electrophoresis. The SDS gels were electrophoresed at 150 V at 15 °C until completion. During the 2D-DIGE, the two samples (mock and fungal inoculation) from each NIL were mixed and run on one gel. Three replicate runs per combination were conducted during the study. Therefore, a total of six gels were run for the NIL pair to achieve the statistical significance needed to confirm protein spots (Figure 1).

Gels were scanned using Typhoon TRIO (GE Healthcare, Pittsburgh, PA, USA) at a 100 µm (pixel size) resolution. The images were analyzed for protein spots using the Image QuantTL software (version 6.0, GE-Healthcare, Pittsburgh, PA, USA) and then subjected to in-gel analysis and cross-gel analysis for the selection of DAP spots using DeCyder software, version 6.5 (GE Healthcare, Pittsburgh, PA, USA). The gel images of the mock inoculations (260-1-1-2 (the fhb1+ NIL) = 2C and 260-1-1-4 (the fhb1- NIL) = 4C) were assigned as the control group, and the gel images of the *Fusarium*-inoculated samples (260-1-1-2 (the fhb1+ NIL) = 2F and 260-1-1-4 (the fhb1- NIL) = 4F) were assigned as treatments (Figure S1).

The protein spots of interest on the gel were selected based on statistical analysis with a cut-off value of 1.5-fold change in the protein abundance and a *p* value of ≤ 0.05. The selected gel spots (Figures S2–S4) were picked up from the prep-gels (250 µg total protein) using the Ettan Spot Picker (GE Healthcare, Pittsburgh, PA, USA). The picked gel spots were washed with 400 µL of buffer (10 mM ammonium bicarbonate/50% acetonitrile) for 30 min at room temperature. Afterwards, the supernatant was discarded, and the gel pieces were air-dried. Finally, in-gel protein digestion was carried out using a modified porcine trypsin protease (Trypsin Gold, Promega, Madison, WI, USA) followed by incubation at 37°C for 5 h. The reaction was stopped by adding 2 µL of 1% TFA. The digested tryptic peptides from each gel piece were desalted using Zip-tip C18 (Millipore, Billerica, MA, USA), eluted from the Zip-tip with 0.5 μL of matrix solution (5 mg mL^−1^ α-cyano-4-hydroxycinnamic acid in 50% acetonitrile, 0.1% TFA and 25 mM ammonium bicarbonate) and spotted into a well on a MALDI plate. MALDI-TOF MS and TOF/TOF tandem MS were performed on a 5800 Mass Spectrometer (AB Sciex, Framingham, MA). The MALDI-TOF mass spectra were produced in the reflectron positive ion mode. An average of 4000 laser shots per fragmentation spectrum were applied to each of the 5–10 most abundant ions present in each sample. Both the resulting peptide mass from the MS-MS analysis and the associated fragmentation spectra were submitted to MASCOT version 2.4 (Matrix Sciences, London, UK) and searched against the database. The searches were performed as described by Poschman et al. [110]. A search in the National Center for Biotechnology Information’s non-redundant (NCBInr) database was performed without constraining the protein MW or the pI value and with the variable carbamidomethylation of cysteine, the oxidation of methionine residues, and a mass tolerance of 100 ppm. Proteins with a protein score ≥72 were considered as correctly assigned. However, in the results and discussion section, we included a few protein identifications with a score ≤72 that have been supported by independent published work. The low score of these protein identifications might be due to several reasons, including poor ionization. The protein identifications with a C.I. (confidence interval) ≥ 95% were considered statistically significant. The protein peptide summary for all identified spots is listed in Table S2.

### 3.3. Real-Time RT-PCR Validation of Proteomic Data

To independently validate the data generated by this proteomic experiment, seven proteins (Spots #20, #47, #57, #62, #64, #67 and #71) were randomly selected. The differential mRNA expression of the corresponding genes was determined by real-time RT-PCR. Primers (Table S1) were designed using PrimerQuest software (Integrated DNA Technologies, Coralville, IA, USA) per the standard parameters for real-time RT-PCR [111].

Total RNA was extracted from the same spikelet samples of the NILs that were used for proteomics using TRIzol^®^ RNA Isolation Reagent (Invitrogen, Carlsbad, CA, USA). The quality and quantity of the RNA samples were assessed using a NanoDrop ND-1000 UV-Vis spectrophotometer (Life Technologies, Grand Island, NY, USA). Reverse transcription was performed in a 20 μL reaction with 2 µg of total RNA using the Superscript III enzyme (Invitrogen, Carlsbad, CA, USA) with the oligo (dT)15 primer (Promega, Madison, WI, USA). Then, the reverse transcription products were diluted 5-fold. A total of 1 µL of the diluted cDNA was used per 10 µL reaction with the SYBR green-I master mix using a 7900 HT Fast Real-Time PCR System (ABI, Foster City, CA, USA). The PCR program was as follows: 10 min at 94 °C; 40 cycles of 20 s at 94°C, 30 s at the melting temperature, and 30 s at 72 °C; and 3 min at 72 °C. A housekeeping gene of wheat, β-actin, was used as the reference gene to normalize the Ct values. All samples were run on the same plate. For each sample, three technical repeats were performed. The Student’s t test was performed for the reference gene across the replications (*p* ≤ 0.05). Fold changes were calculated using the 2^−ΔΔCt^ method [112]. A two-fold change was set as the threshold for significant differences.

### 3.4. Computational Analysis and Protein–Protein Interaction Predictions 

We used a computational approach for a few selected proteins to identify potential regulatory partners and to analyze the functional correlation. The two-step method was as follows: First, we blasted (PSI-Blast) the sequence of MS/MS-based identified proteins to obtain proteins with identical sequences [113]. Then, we selected the best homologous candidate based on identity and sequence similarity using ClustalW [114]. Second, to search the variety of functional protein–protein interactions with our candidate protein we used the online database resource STRING (http://string-db.org/) version 9.1 [115]. The obtained primary interactions were portrayed using Cytoscape software version 3.0.1 [116,117].

### 3.5. Clustering and Statistical Analyses 

The results from three biological repeats per treatment and three technical repeats per assay were compared for the control and the treatment (*Fusarium*-inoculated) groups. Observed differences in spot abundance on the 2D-DIGE gels were statistically evaluated using Student’s *t*-test. All experimental mean values and standard deviations were calculated from the three independent sets (n = 3) of harvests (biological replicates) and from three replicates of each of the three harvests (technical replicates). The statistical significance of the results was evaluated, and *p* ≤ 0.05 was considered statistically significant for the comparisons [118]. We used the log_2_-transformed abundance values of all protein spots of interest to generate the heat map, which was constructed using JAVA TREEVIEW [119,120].

## 4. Conclusions

**Figure 4:**
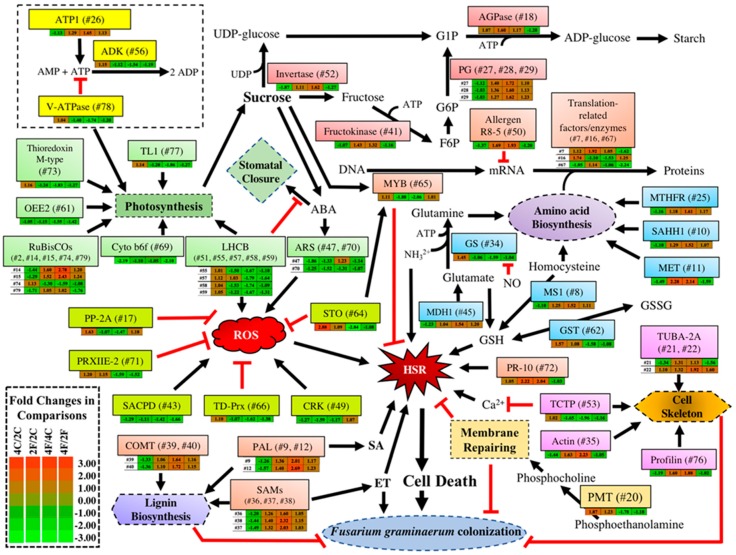
An illustration of major proteomic changes, their interactions and their impacts on the metabolisms and FHB pathogenesis of wheat. The arrow-tipped black lines indicate promotive actions of the proteins, and the T-tipped red lines indicate inhibitive actions of the proteins. The differentially accumulated proteins are in solid squares, and the heat maps underneath the squares indicate their fold changes in the four comparisons of this study. 2C: the mock-inoculated fhb1+ NIL; 2F: the *Fusarium*-inoculated fhb1+ NIL; 4C: the mock-inoculated fhb1- NIL; 4F: the *Fusarium*-*inoculated* fhb1- NIL; ADK: adenylate kinase; ADP: adenosine diphosphate; AGPase: glucose-1-phosphate adenylyltransferase large subunit; AMP: adenosine monophosphate; APX: thylakoid-bound ascorbate peroxidase; ARS: abscisic stress ripening protein; ATP: adenosine triphosphate; ATP1: ATP synthesis 1; COMT: caffeic acid 3-*O*-methyltransferase; CRK: cysteine-rich receptor-like protein kinase; Cyto b6f: chloroplastic cytochrome b6-f complex iron-sulfur subunit; ET: ethylene; F6P: fructose 6-phosphate; G1P: glucose-1-phosphate; G6P: glucose-6-phoaphate; GS: glutamine synthetase; GSH: glutathione; GSSG: glutathione disulfide; GST: glutathione transferase F5; HSR: hypersensitive reactions; LHCB: chlorophyll a/b binding protein; MDH1: Malate dehydrogenase 1; MET: 5-methyltetrahydropteroyltriglutamate; MIPS1: Myo-inositol-1-phosphate synthase 1; MS1: methionine synthase; MTHFR: 5,10-methylene-tetrahydrofolate reductase; MYB: MYB transcription factor; NO: nitric oxide; OEE2: oxygen-evolving enhancer protein 2; PAL: phenylalanine ammonia-lyase; PG: phosphoglucomutase; PMT: phosphoethanolamine methyltransferase; PP2A: protein phosphate 2A structural subunit; PR-10: pathogenesis-related protein 10; PRXIIE-2: peroxiredoxin-2; ROS: radical oxygen species; RPT2: root phototropism protein 2; RuBisCO: ribulose-1,5-bisphosphate carboxylase/oxygenase; SA: salicylic acid; SAD: stearoyl-ACP desaturase; SAHH1: adenosylhomocysteinase; SAMS: S-adenosylmethionine synthases; STO: salt tolerant protein; TCTP: translationally-controlled tumor protein; TD-Prx: thioredoxin-dependent peroxidase; TL1: 15 kDa thylakoid lumenal protein 1; TRXm: thioredpxin M-type; TUBA-2A: alpha tubulin-2A; UDP: uridin diphosphate; V-ATPase: vacuolar ATPase.

Figure 4 illustrates the major proteomic changes discovered by this study and their impacts on the metabolism and FHB pathogenesis in wheat. Basically, this proteomic study revealed a significant difference in the number of DAPs between the two NILs. In total, 50 DAPs were found to be specific to the fhb1- NIL, and only seven DAPs were specific to the fhb1+ NIL. We observed that 17 of the 18 stress-related DAPs were either not significant in the fhb1+ NIL or were significant but behaved similarly in both NILs. The only two stress-related DAPs (Proteins #49 and #70) that were specific to the fhb1+ NIL were significantly down-regulated after pathogen inoculation. Moreover, 12 of the 15 photosynthesis-related DAPs were down-regulated after inoculation in the absence of *Qfhb1*. These remarkable differences in the number of DAPs between the NILs imply that *F. graminearum* inoculation had a greater impact on the fhb1- NIL than on the fhb1+ NIL.

The proteomic data show that the absence of *Qfhb1* constitutively impairs the fhb1- NIL capabilities of photosynthesis, protein production, the detection and reduction of oxidative and other stresses, GSH removal, and the repair of membrane damages, while hindering the sucrose-signaling pathway by significantly reducing the sucrose pool by enhancing starch synthesis from sucrose. Hence, the fhb1- NIL is significantly more vulnerable than the fhb1+ NIL to *F. graminearum* attack. In the absence of *Qfhb1*, pathogen infection might further negatively impact all the steps in photosynthesis, except for that of carbon fixation, causing a significantly lower proton gradient across the relevant membranes, which could drastically alter intracellular environments and imbalance redox states.

The production of ROS, GSH and NO could be significantly induced in the fhb1- NIL by pathogen inoculation, and the ability to remove ROS and GHS could be significantly reduced simultaneously. Meanwhile, amino acid synthesis and protein production could be significantly reduced, and activities to detoxify trichothecene toxins could be suppressed. To counter these challenges, wheat could launch strong HSR-centered defense responses, which might be mainly mediated by SA- and ABA/ET-signaling pathways, leading to infected cells suicide and FHB development. HSR is known to be a major strategy by the host to limit colonization of biotrophic pathogens, but, in the case of FHB pathogenesis, HSR seems to function as a major pathogenic factor. Generally, we observed these defense activities were much more intense in the fhb1- NIL than in the fhb1+ NIL. The proteomic data also show that the main function of *Qfhb1* could be to alleviate the negative impacts of HSR defense responses, to increase GHS detoxification, to reduce ROS signaling and plant immune responses, to strengthen cell membranes with stronger repairing activities and to enhance translation activities. This observation agrees with that of Zhuang et al. [22], who observed that a key functional component of *Qfhb1*, WFhb1_fc1, was suppressed by pathogen infection in the FHB-susceptible genotypes but not in the FHB-resistant genotypes. These observations imply that the FHB resistance presented by *Qfhb1* may simply be unresponsive to suppression by pathogen infection. Our proteomic data support this hypothesis and further imply the following: (1) wheat responds to *F. graminearum* infection most likely via SA- and ABA/ET-signaling pathways; (2) FHB development is mainly due to HSR caused by the ROS, NO and GSH bursts induced by *F. graminearum* infection; and (3) the alleviation of these resistance responses is the main function of *Qfhb1*.

Genes for PFT [20], GDSL [17] and HRC [65] have been reported to be the functional components of the QTL. Based on the published sequence of the *Qfhb1* marker interval by Schweiger et al. [17], eight DAPs (Proteins #1, #3, #7, #20, #26, #49, #68 and #80) revealed in this proteomic study could be encoded by genes in the *Qfhb1* marker interval. We did not identify any DAP that is likely a PFT or a GDSL, but Protein #53 (TCTP) could be *HRC* because both Protein #53 and *HRC* have a calcium-binding domain. Schweiger et al. observed a strong constitutive expression of the gene for Protein #53 in the fhb1+ genotype but this gene was silenced in the fhb1- genotype at the transcriptional level [17]. Here, we observed a strong constitutive expression of Protein #53 and its significant down-regulation by *F. graminearum* inoculation in both NILs. The proteomic data revealed a diverse number of pathways that contribute to FHB development. To counter the pathogen infection and FHB development, wheat plants probably need more than one gene in the *Qfhb1* marker interval to work together. Alternatively, if there is only a single *Qfhb1* gene responsible for the resistance that this QTL could confer, this gene must encode a master regulator to all or at least most of the described pathways. In this case, neither PFT nor HRC is qualified as a master controller. GDSLs are a large family in plants. Overexpressing GLIP1 in Arabidopsis induced resistance to a range of pathogens, including hemitrophic pathogen *P. syringae* pv. tomato (Pst) DC3000 [27]. However, GDSL could be a down-stream player since ET-signaling is required for its expression and function [121]. Of the fhb1+ NIL-specific DAPs we revealed, Proteins #6 and #44 are uncharacterized. In addition, the exact functions of Proteins #13 and #19 are unclear. Further study of these four DAPs may perhaps provide additional clues about the regulator(s), the FHB development and the FHB resistance presented by *Qfhb1* in wheat.

In conclusion, our comparative proteomic study of FHB pathogenesis in the wheat NILs suggests that the initiation of FHB development mainly results from HSR in response to the invading *F. graminearum* and that *Qfhb1* largely functions to either reduce the suicide response of the host cells or to make the host cells not respond to the infection. Additionally, none of the 80 DAPs were found to be encoded by a previously reported *Qfhb1* gene. Our data suggest that wheat may use more than one functional gene in *Qfhb1* to counter the observed susceptible activities in FHB pathogenesis since several diverse pathways were found to be involved. Alternatively, a master regulator for all of these diverse pathways may exist in this QTL but was not identified in the DAPs we studied. Further analysis of the DNA sequence in the *Qfhb1* interval for non-coding RNAs or the study of additional DAPs may help clarify the role of *Qfhb1* in FHB resistance.

## Figures and Tables

**Table 1:** Differentially accumulated proteins and their abundance ratios in the two studied NILs under mock- and *Fusarium*-inoculations as revealed by 2D-DIGE followed by MALDI-MS/MS.

Category	ID	Annotation	GenBank ID	kDa	pI	4C/2C	2F/2C	4F/4C	4F/2F
1	80 **	60S ribosomal protein L5	gi|360042516	34.44	9.3	**−6.87**	**3.53**	**19.54**	−1.24
	24	Myo-inositol-1-phosphate synthase	gi|90289596	49.68	4.9	**−2.51**	1.20	**3.43**	1.14
	12	Phenylalanine ammonia-lyase	gi|3024363	75.89	6.1	**−1.57**	1.40	**2.69**	1.23
	52	Vacuolar invertase	gi|3219509	56.4	5.8	**−1.87**	1.11	**1.62**	−1.27
	1 **	Glycosyltransferase	gi|56409844	65.08	6.0	**−1.71**	−1.18	1.19	−1.21
	47	Abscisic stress ripening protein	gi|357163453	25.95	5.1	**−1.86**	−1.33	1.23	−1.14
	69	Cytochrome b6-f complex iron-sulfur subunit, chloroplastic	gi|68566191	23.71	8.5	**−2.19**	−1.10	−1.05	**−2.10**
	67	Eukaryotic translation initiation factor 5A1	gi|74048999	17.35	5.7	**−1.85**	1.14	−1.06	**−2.24**
	79	Ribulose bisphosphate carboxylase small chain clone 512	gi|132107	13.05	5.8	**−1.71**	1.05	1.02	**−1.76**
2	17	Protein phosphatase 2A structural subunit	gi|328775741	65.46	5.0	**1.63**	−1.07	−1.47	1.18
	16	Protein disulfide isomerase	gi|222446340	56.64	5.0	**1.74**	−1.10	**−1.53**	1.25
	20	Phosphoethanolamine methyltransferase	gi|17887465	56.82	5.2	**1.87**	1.23	**−1.78**	−1.18
	62	Glutathione transferase F5	gi|23504745	23.42	5.8	**1.57**	1.08	**−1.58**	−1.08
	64	Salt tolerant protein	gi|63021412	17.05	4.7	**2.88**	1.09	**−2.84**	−1.08
3	34	Glutamine synthetase isoform GSr1	gi|40317416	38.71	5.4	1.45	−1.06	**−1.59**	−1.04
	51	Chlorophyll a-b binding protein of LHCII type III, chloroplastic-like, predicted	gi|357122389	28.56	5.0	1.01	−1.39	**−1.51**	−1.07
	56	Adenylate kinase, chloroplastic-like	gi|357139457	31.43	7.1	1.15	−1.12	**−1.54**	−1.19
	59	Light-harvesting complex I chlorophyll a/b binding protein 3	gi|79320443	23.73	5.6	1.05	−1.22	**−1.67**	−1.31
	61	20S proteasome beta-4 subunit	gi|52548238	23.31	5.6	−1.05	−1.15	**−1.55**	−1.42
	66	Thioredoxin-dependent peroxidase	gi|256708473	17.39	5.2	1.10	−1.07	**−1.62**	−1.38
	68	Eukaryotic translation initiation factor 5A1	gi|74048999	17.35	5.7	1.29	−1.05	**−1.66**	−1.22
	73	Thioredoxin M-type, chloroplastic; Precursor	gi|11135474	19.12	8.7	1.16	−1.24	**−1.83**	−1.27
	74	Ribulose bisphosphate carboxylase small chain clone 512 (Fragment)	RBS3_WHEAT	13.05	5.8	1.13	−1.30	**−1.59**	−1.08
	77	Thylakoid lumenal 15 kDa protein 1, chloroplastic, predicted	gi|225449424	23.42	6.5	1.14	−1.28	**−1.86**	−1.27
	78	Vacuolar ATPase subunit F	gi|94537548	14.37	5.3	1.04	−1.40	**−1.74**	−1.20
	57	Chlorophyll a-b binding protein	gi|225690794	26.96	5.4	1.12	1.03	**−1.79**	**−1.64**
	71	Peroxiredoxin-2E-2, chloroplastic-like, predicted	gi|357139104	23.21	6.7	1.20	1.15	**−1.59**	**−1.52**
4	22	Alpha tubulin-2A	gi|90289596	49.68	4.9	1.10	1.32	**1.92**	**1.60**
	3 **	Glycosyltransferase	gi|56409844	65.08	6.0	1.01	1.49	**1.68**	1.14
	2	Ribulose bisphosphate carboxylase small chain PW9, chloroplastic	RBS2_WHEAT	19.44	8.5	−1.20	1.35	**1.80**	1.11
	4 *	COP9 signalosome subunit	gi|159463648	50.44	6.0	−1.05	1.12	**1.52**	1.29
	5	Predicted barley protein	gi|326503600	99.43	5.2	−1.04	1.34	**1.56**	1.13
	8	Methionine synthase 1 enzyme	gi|68655495	84.51	5.7	−1.10	1.25	**1.52**	1.11
	9	Phenylalanine ammonia-lyase	gi|3024363	75.89	6.1	−1.26	1.36	**2.01**	1.17
	10	Adenosylhomocysteinase	SAHH_WHEAT	53.4	5.7	−1.10	1.29	**1.52**	1.07
	23	Mitochondrial-processing peptidase subunit beta-like, predicted	gi|357113428	58.38	5.6	−1.36	1.24	**1.66**	−1.01
	25	5,10-methylene-tetrahydrofolate reductase	gi|115589742	64.83	5.9	−1.16	1.18	**1.61**	1.17
	26	ATPase subunit 1	gi|81176509	55.27	5.7	−1.13	1.29	**1.65**	1.13
	27	Phosphoglucomutase	gi|18076790	62.75	5.7	−1.12	1.40	**1.72**	1.10
	28	Phosphoglucomutase	gi|18076790	62.75	5.7	−1.03	1.36	**1.60**	1.13
	29	Phosphoglucomutase	gi|18076790	62.75	5.7	−1.03	1.27	**1.62**	1.23
	36	S-adenosylmethionine synthase	gi|223635315	43.15	5.6	−1.20	1.26	**1.60**	1.05
	38	S-adenosylmethionine synthase	gi|223635315	43.15	5.6	−1.44	1.40	**2.32**	1.15
	37	Acyl transferase 5, putative	gi|151175359	46.83	5.5	−1.49	1.32	**2.03**	1.03
	39	Caffeic acid 3-*O*-methyltransferase	gi|145321007	38.57	5.7	−1.33	1.06	**1.64**	1.16
	40	Caffeic acid 3-*O*-methyltransferase	gi|145321007	38.57	5.7	−1.36	1.10	**1.72**	1.15
	42	60S acidic ribosomal protein (Os08g0130500)	gi|115474653	34.36	5.4	−1.36	1.46	**1.76**	−1.13
	45	Malate dehydrogenase 1, mitochondrial-like, predicted	gi|357132456	35.37	8.5	−1.23	1.04	**1.54**	1.20
5	11	5-methyltetrahydro-pteroyltriglutamate/hom-ocysteine methyl-transferase-like, predicted	gi|357155679	84.64	6.0	−1.49	**2.28**	**2.14**	**−1.59**
	72	Pathogenesis related protein 10	gi|196051131	17.05	5.2	1.05	**2.22**	**2.04**	−1.03
	50	Group 5/9 grass pollen allergen R8-5	gi|365769201	24.91	5.6	−1.37	**1.69**	**1.93**	−1.20
	35	Actin-97-like, predicted	gi|357135037	41.7	5.2	−1.44	**1.63**	**2.23**	−1.05
	76	Profilin	gi|300807845	14.18	4.9	−1.19	**1.60**	**1.88**	−1.02
	14	Ribulose-1,5-bisphosphate carboxylase/oxygenase large subunit	gi|14017580	52.82	6.2	−1.44	**1.60**	**2.78**	1.20
	15	Ribulose-1,5-bisphosphate carboxylase/oxygenase large subunit	gi|14017580	52.82	6.2	−1.29	**1.52**	**2.43**	1.24
6	33	Predicted barley protein	gi|326512860	36.77	5.0	1.22	**−1.76**	**−1.99**	1.08
	53	Translationally-controlled tumor protein	gi|146285306	18.77	4.6	1.02	**−1.65**	**−1.96**	−1.16
	54	Predicted barley protein	gi|326520557	16.18	4.8	−1.03	**−1.55**	**−1.66**	−1.11
	55	Chlorophyll a/b binding protein, predicted	gi|302566696	28.2	5.1	1.01	**−1.50**	**−1.67**	−1.10
	58	Chlorophyll a-b binding protein CP24 10A, predicted	gi|356525886	27.67	6.2	1.04	**−1.53**	**−1.74**	−1.09
	65	MYB-related protein	gi|359952782	48.05	8.9	1.11	**−1.88**	**−2.06**	1.01
7	13 **	Mechanosensitive ion channel	gi|86439721	34.71	8.5	−1.21	**2.51**	1.48	**−2.05**
	6	Predicted Micromona protein	gi|255083775	44.95	10.4	1.07	**2.27**	1.24	**−1.72**
	7	Eukaryotic translation initiation factor 3 subunit D-like, predicted	gi|357128487	78.27	5.7	1.12	**1.92**	1.05	**−1.62**
	44	Uncharacterized maize protein LOC100273160	gi|226528643	39.94	6.1	−1.03	**1.65**	1.33	−1.27
	18	Glucose-1-phosphate adenylyltransferase large subunit, chloroplastic/amyloplastic (Fragment)	GLGL3_WHEAT	55.52	6.6	1.07	**1.60**	1.17	−1.28
8	49 **	Root phototropism 2, putative, expressed	gi|108862102	28.47	9.7	−1.27	**−1.59**	−1.17	1.07
	70	Abscisic acid stress ripening 1 protein	gi|321155395	10.45	6.6	−1.25	**−1.52**	−1.31	−1.07
9	19 *	Starch synthase IIa-3	gi|8953573	86.74	6.3	1.43	1.18	1.40	**1.70**
	21	Alpha tubulin-2A	gi|90289596	49.68	4.9	−1.34	1.31	1.13	**−1.56**
	43	Stearoyl-ACP desaturase	gi|319739540	44.43	8.2	−1.29	−1.11	−1.42	**−1.66**

*: low confidence; **: No confidence; 2C and 2F: the mock- and the FHB-inoculated NIL 260-1-1-2 (fhb1+), respectively; 4C and 4F: the mock- and the FHB-inoculated NIL 260-1-1-4 (fhb1-), respectively; Significant fold change (fold change ≥ 1.5) are in red.

## Supplementary Materials

The following are available online at http://www.mdpi.com/2076-0817/7/3/58/s1, Figure S1: 2D-DIGE representation of the Cy2/Cy5 overlay of gels. (A) Proteomic images of the mock-inoculated fhb1+ NIL (2C) and fhb1- NIL (4C) and the overlaid images of both for abundance comparison (2C/4C). (B) Proteomic images of the FHB-inoculated fhb1+ NIL (2F) and fhb1- NIL (4F) and the overlaid images of both for the abundance comparison (2F/2F). Figure S2: An example of DeCyder software analysis showing the proteomic expression of starch synthase IIa-3 (Protein #19) in the FHB-inoculated fhb1+ NIL (2F) and fhb1- NIL (4F). (A) Gel images showing a spot (in yellow) in both 2F (left) and 4F (right). (B) 3D view of the differential expression of starch synthase IIa-3 in 2F (left) and 4F (right). Figure S3: An example of DeCyder software analysis showing the proteomic expression of eukaryotic translation initiation factor 3 subunit D-like protein (Protein #7) in the FHB-inoculated fhb1+ NIL (2F) and fhb1- NIL (4F). (A) Gel images showing a spot (in yellow) in both 2F (left) and 4F (right). (B) 3D view of the differential expression of eukaryotic translation initiation factor 3 subunit D-like protein in 2F (left) and 4F (right). Figure S4: An example of DeCyder software analysis showing the proteomic expression of peroxiredoxin-2E-2 protein (Protein #71) in the FHB-inoculated fhb1+ NIL (2F) and fhb1- NIL (4F). (A) Gel images showing a spot (in yellow) in both 2F (left) and 4F (right). (B) 3D view of the differential expression of peroxiredoxin-2E-2 protein in 2F (left) and 4F (right). Figure S5: Validation of the 2D-DIGE-MS/MS results by quantitative real-time PCR. Bar diagram shows the compared fold change values of the proteins and their mRNA expression levels determined by real-time PCR. The protein spots used for validation were #67 (eukaryotic translation initiation factor 5A1), #47 (abscisic stress ripening protein), #62 (glutathione transferase F5), #57 (chlorophyll a-b binding protein), #71 (peroxiredoxin-2E-2), #64 (salt tolerant protein) and #20 (phosphoethanolamine methyltransferase). 2F/2C indicates the comparison between the *Fusarium*-inoculated and the mock-inoculated fhb1+ NIL samples, and 4F/4C indicates the comparison between the *Fusarium*-inoculated and the mock-inoculated fhb1- NIL samples. Figure S6: Functional classification of DAPs based on their Gene Ontology annotations. The individual pies have been categorized to show: total DAPs identified in the experiment, constitutively expressed DAPs identified in the experiment, shared DAPs between fhb1+ NIL and fhb1- NIL, DAPs specifically expressed in fhb1- NIL and DAPs specifically expressed in fhb1 + NIL. Figure S7: A diagram showing the regulatory network controlled by pathogenesis-related protein 10 (PR-10). The regulatory network comprises nine nodes and 34 edges, showing functional protein–protein interactions. *Zea mays* PR-10 (yellow box) is the central regulatory component of the network, to better understand the wide range of defense-related regulatory interactions. K7TGS7 (terpene synthase 6), B6TL22 (zeamatin precursor), B4FVP5 (pathogenesis related protein-4), B6SY34 (a putative uncharacterized protein), A3FMA3 (putative serine type endopeptidase inhibitor), B8A260 (glucan endo-1,3-beta-glucosidase), B6SLW8 (cytochrome b5) and B6SN21 (polygalacturonase inhibitor) are indicated in the figure. Figure S8: The heat map of proteins identified during pre- and post-infection with *F. graminearum* showing differential expression of 80 proteins was created using Euclidean distance similarity metric and complete linkage method. Hierarchical clustering was done using Gene Cluster 3.0 software based on the log2-transformed ratios of protein abundance as shown in Table 1. Clusters were visualized using JAVA TREEVIEW software. (“C” and “F” correspond to control and after infection condition, respectively; “2” indicates the resistant 260-1-1-2 [fhb1 +] NILs; and “4” indicates the susceptible 260-1-1-4 [fhb1-] NILs; corresponding numbers on Y axis indicates protein spot numbers; * indicates proteins with no confidence). Table S1. Primer sequences used for real-time RT-PCR. Table S2. Top 80 differentially accumulated proteins identified by the 2D-DIGE and MALDI-MS/MS assays. Table S3. Fold changes of the proteins identified by 2D-DIGE and MALDI-MS/MS analyses.

## References

[1] Desjardins A.E., Hohn T.M. (1997). Mycotoxins in plant pathogenesis. Mol. Plant-Microbe Interact..

[2] Snijders C.H.A., Perkowski J. (1990). Effects of head blight caused by *Fusarium culmorum* on toxin content and weight of wheat kernels. Phytopathology.

[3] Parry D.W., Jenkinson P., Mcleod L. (1995). Fusarium ear blight (Scab) in small grain cereals—A review. Plant Pathol..

[4] Schroeder H.W., Christensen J.J. (1963). Factors affecting resistance of wheat to scab caused by *Gibberella Zeae*. Phytopathology.

[5] Foroud N.A., Ouellet T., Laroche A., Oosterveen B., Jordan M.C., Ellis B.E., Eudes F. (2012). Differential transcriptome analyses of three wheat genotypes reveal different host response pathways associated with Fusarium head blight and trichothecene resistance. Plant Pathol..

[6] Anderson J.A., Stack R.W., Liu S., Waldron B.L., Fjeld A.D., Coyne C., Moreno-Sevilla B., Fetch J.M., Song Q.J., Cregan P.B. (2001). DNA markers for Fusarium head blight resistance QTLs in two wheat populations. Theor. Appl. Genet..

[7] Bai G.H., Kolb F.L., Shaner G., Domier L.L. (1999). Amplified fragment length polymorphism markers linked to a major quantitative trait locus controlling scab resistance in wheat. Phytopathology.

[8] Basnet B.R., Glover K.D., Ibrahim A.M.H., Yen Y., Chao S.M. (2012). A QTL on chromosome 2DS of ‘Sumai 3’ increases susceptibility to Fusarium head blight in wheat. Euphytica.

[9] Buerstmayr H., Lemmens M., Hartl L., Doldi L., Steiner B., Stierschneider M., Ruckenbauer P. (2002). Molecular mapping of QTLs for Fusarium head blight resistance in spring wheat. I. Resistance to fungal spread (type II resistance). Theor. Appl. Genet..

[10] Waldron B.L., Moreno-Sevilla B., Anderson J.A., Stack R.W., Frohberg R.C. (1999). RFLP mapping of QTL for fusarium head blight resistance in wheat. Crop Sci..

[11] Zhou W.C., Kolb F.L., Bai G.H., Shaner G., Domier L.L. (2002). Genetic analysis of scab resistance QTL in wheat with microsatellite and AFLP markers. Genome.

[12] Liu J.J., Ekramoddoullah A.K.M. (2006). The family 10 of plant pathogenesis-related proteins: Their structure, regulation, and function in response to biotic and abiotic stresses. Physiol. Mol. Plant Pathol..

[13] McMullen M., Jones R., Gallenberg D. (1997). Scab of wheat and barley: A re-emerging disease of devastating impact. Plant Dis..

[14] Wisniewska H., Kowalczyk K. (2005). Resistance of cultivars and breeding lines of spring wheat to *Fusarium culmorum* and powdery mildew. J. Appl. Genet..

[15] Choulet F., Wicker T., Rustenholz C., Paux E., Salse J., Leroy P., Schlub S., Le Paslier M.C., Magdelenat G., Gonthier C. (2010). Megabase level sequencing reveals contrasted organization and evolution patterns of the wheat gene and transposable element spaces. Plant Cell.

[16] Liu S.X., Pumphrey M.O., Gill B.S., Trick H.N., Zhang J.X., Dolezel J., Chalhoub B., Anderson J.A. (2008). Toward positional cloning of *Fhb1*, a major QTL for Fusarium head blight resistance in wheat. Cereal Res. Commun..

[17] Schweiger W., Steiner B., Vautrin S., Nussbaumer T., Siegwart G., Zamini M., Jungreithmeier F., Gratl V., Lemmens M., Mayer K.F.X. (2016). Suppressed recombination and unique candidate genes in the divergent haplotype encoding *Fhb1*, a major Fusarium head blight resistance locus in wheat. Theor. Appl. Genet..

[18] Hofstad A.N., Nussbaumer T., Akhunov E., Shin S., Kugler K.G., Kistler H.C., Mayer K.F.X., Muehlbauer G.J. (2016). Examining the transcriptional response in wheat *Fhb1* near-isogenic lines to *Fusarium graminearum* infection and deoxynivalenol treatment. Plant Genome.

[19] Pumphrey M.O. (2007). Towards Map-Based Cloning of Fusarium Head Blight Resistance QTL Fhb1 and Non-Additive Expression of Homoeologous Genes in Allohexaploid Wheat. Ph.D. Thesis.

[20] Rawat N., Pumphrey M.O., Liu S., Zhang X., Tiwari V.K., Ando K., Trick H.N., Bockus W.W., Akhunov E., Anderson J.A. (2016). Wheat *Fhb1* encodes a chimeric lectin with agglutinin domains and a pore-forming toxin-like domain conferring resistance to Fusarium head blight. Nat. Genet..

[21] Xiao J., Jin X.H., Jia X.P., Wang H.Y., Cao A.Z., Zhao W.P., Pei H.Y., Xue Z.K., He L.Q., Chen Q.G. (2013). Transcriptome-based discovery of pathways and genes related to resistance against Fusarium head blight in wheat landrace Wangshuibai. BMC Genom..

[22] Zhuang Y., Gala A., Yen Y. (2013). Identification of functional genic components of major fusarium head blight resistance quantitative trait loci in wheat cultivar Sumai 3. Mol. Plant-Microbe Interact..

[23] Lemmens M., Scholz U., Berthiller F., Dall’Asta C., Koutnik A., Schuhmacher R., Adam G., Buerstmayr H., Mesterhazy A., Krska R. (2005). The ability to detoxify the mycotoxin deoxynivalenol colocalizes with a major quantitative trait locus for fusarium head blight resistance in wheat. Mol. Plant-Microbe Interact..

[24] Gunnaiah R., Kushalappa A.C., Duggavathi R., Fox S., Somers D.J. (2012). Integrated metabolo-proteomic approach to decipher the mechanisms by which wheat QTL (*Fhb1*) contributes to resistance against *Fusarium graminearum*. PLoS ONE.

[25] Zhuang Y. (2014). Genetic Dissection of Fusarium Head Blight in Wheat (*Triticum aestivum* L.). Ph.D. Thesis.

[26] Li G., Yen Y. (2008). Jasmonate and ethylene signaling pathway may mediate Fusarium head blight resistance in wheat. Crop Sci..

[27] Kwon S.J., Jin H.C., Lee S., Nam M.H., Chung J.H., Il Kwon S., Ryu C.M., Park O.K. (2009). GDSL lipase-like 1 regulates systemic resistance associated with ethylene signaling in Arabidopsis. Plant J..

[28] Bhadauria V., Banniza S., Wang L.X., Wei Y.D., Peng Y.L. (2010). Proteomic studies of phytopathogenic fungi, oomycetes and their interactions with hosts. Eur. J. Plant Pathol..

[29] Mehta A., Brasileiro A.C.M., Souza D.S.L., Romano E., Campos M.A., Grossi-De-Sa M.F., Silva M.S., Franco O.L., Fragoso R.R., Bevitori R. (2008). Plant-pathogen interactions: What is proteomics telling us?. FEBS J..

[30] Quirino B.F., Candido E.S., Campos P.F., Franco O.L., Kruger R.H. (2010). Proteomic approaches to study plant-pathogen interactions. Phytochemistry.

[31] Yang F., Jacobsen S., Jorgensen H.J.L., Collinge D.B., Svensson B., Finnie C. (2013). *Fusarium graminearum* and its interactions with cereal heads: Studies in the proteomics era. Front. Plant Sci..

[32] Finnie C., Melchior S., Roepstorff P., Svensson B. (2002). Proteome analysis of grain filling and seed maturation in barley. Plant Physiol..

[33] Thurston G., Regan S., Rampitsch C., Xing T. (2005). Proteomic and phosphoproteomic approaches to understand plant-pathogen interactions. Physiol. Mol. Plant Pathol..

[34] Das A., Paudel B., Rohila J.S., Al-Khayri J.M., Jain S.M., Johnson D.V. (2015). Potentials of Proteomics in Crop Breeding. Advances in Plant Breeding Strategies: Breeding, Biotechnology and Molecular Tools.

[35] Wang Y., Yang L.M., Xu H.B., Li Q.F., Ma Z.Q., Chu C.G. (2005). Differential proteomic analysis of proteins in wheat spikes induced by *Fusarium graminearum*. Proteomics.

[36] Zhang X.H., Fu J.M., Hiromasa Y., Pan H.Y., Bai G.H. (2013). Differentially expressed proteins associated with fusarium head blight resistance in wheat. PLoS ONE.

[37] Zhou W.C., Kolb F.L., Riechers D.E. (2005). Identification of proteins induced or upregulated by fusarium head blight infection in the spikes of hexaploid wheat (*Triticum aestivum*). Genome.

[38] Beranova-Giorgianni S. (2003). Proteome analysis by two-dimensional gel electrophoresis and mass spectrometry: Strengths and limitations. Trends Anal. Chem..

[39] Lilley K.S., Dupree P. (2006). Methods of quantitative proteomics and their application to plant organelle characterization. J. Exp. Bot..

[40] Lilley K.S., Friedman D.B. (2004). All about DIGE: Quantification technology for differential-display 2D-gel proteomics. Expert Rev. Proteom..

[41] Schenkluhn L., Hohnjec N., Niehaus K., Schmitz U., Colditz F. (2010). Differential gel electrophoresis (DIGE) to quantitatively monitor early symbiosis- and pathogenesis-induced changes of the *Medicago truncatula* root proteome. J. Proteom..

[42] Unlu M., Morgan M.E., Minden J.S. (1997). Difference gel electrophoresis: A single gel method for detecting changes in protein extracts. Electrophoresis.

[43] Amey R.C., Schleicher T., Slinn J., Lewis M., Macdonald H., Neill S.J., Spencer-Phillips P.T.N. (2008). Proteomic analysis of a compatible interaction between *Pisum sativum* (pea) and the downy mildew pathogen *Peronospora viciae*. Eur. J. Plant Pathol..

[44] Dornez E., Croes E., Gebruers K., Carpentier S., Swennen R., Laukens K., Witters E., Urban M., Delcour J.A., Courtin C.M. (2010). 2-D DIGE reveals changes in wheat xylanase inhibitor protein families due to *Fusarium graminearum* deltaTri5 infection and grain development. Proteomics.

[45] Liu S., Zhang X., Pumphrey M.O., Stack R.W., Gill B.S., Anderson J.A. (2006). Complex microcolinearity among wheat, rice, and barley revealed by fine mapping of the genomic region harboring a major QTL for resistance to Fusarium head blight in wheat. Funct. Integr. Genom..

[46] Cho Y.H., Yoo S.D. (2011). Signaling role of fructose mediated by FINS1/FBP in *Arabidopsis thaliana*. PLoS Genet..

[47] Li P., Wind J.J., Shi X.L., Zhang H.L., Hanson J., Smeekens S.C., Teng S. (2011). Fructose sensitivity is suppressed in Arabidopsis by the transcription factor ANAC089 lacking the membrane-bound domain. Proc. Natl. Acad. Sci. USA.

[48] Engelsdorf T., Horst R.J., Prols R., Proschel M., Dietz F., Huckelhoven R., Voll L.M. (2013). Reduced carbohydrate availability enhances the susceptibility of Arabidopsis toward *Colletotrichum higginsianum*. Plant Physiol..

[49] Ding L.N., Xu H.B., Yi H.Y., Yang L.M., Kong Z.X., Zhang L.X., Xue S.L., Jia H.Y., Ma Z.Q. (2011). Resistance to hemi-biotrophic *F. graminearum* infection is associated with coordinated and ordered expression of diverse defense signaling pathways. PLoS ONE.

[50] Sheehan D., Meade G., Foley V.M., Dowd C.A. (2001). Structure, function and evolution of glutathione transferases: Implications for classification of non-mammalian members of an ancient enzyme superfamily. Biochem. J..

[51] Douglas K.T. (1987). Mechanism of action of glutathione-dependent enzymes. Adv. Enzymol. Relat. Areas Mol. Biol..

[52] Gomez L.D., Noctor G., Knight M.R., Foyer C.H. (2004). Regulation of calcium signalling and gene expression by glutathione. J. Exp. Bot..

[53] Rentel M.C., Knight M.R. (2004). Oxidative stress-induced calcium signaling in Arabidopsis. Plant Physiol..

[54] Vanacker H., Carver T.L.W., Foyer C.H. (2000). Early H_2_O_2_ accumulation in mesophyll cells leads to induction of glutathione during the hyper-sensitive response in the barley-powdery mildew interaction. Plant Physiol..

[55] Durian G., Rahikainen M., Alegre S., Brosche M., Kangasjarvi S. (2016). Protein phosphatase 2A in the regulatory network underlying biotic stress resistance in plants. Front. Plant Sci..

[56] Nagaoka S., Takano T. (2003). Salt tolerance-related protein STO binds to a Myb transcription factor homologue and confers salt tolerance in Arabidopsis. J. Exp. Bot..

[57] Belles-Boix E., Babiychuk E., Van Montagu M., Inze D., Kushnir S. (2000). CEO1, a new protein from *Arabidopsis thaliana*, protects yeast against oxidative damage. FEBS Lett..

[58] Fujibe T., Saji H., Arakawa K., Yabe N., Takeuchi Y., Yamamoto K.T. (2004). A methyl viologen-resistant mutant of Arabidopsis, which is allelic to ozone-sensitive rcd1, is tolerant to supplemental ultraviolet-B irradiation. Plant Physiol..

[59] Xu Y.H., Liu R., Yan L., Liu Z.Q., Jiang S.C., Shen Y.Y., Wang X.F., Zhang D.P. (2012). Light-harvesting chlorophyll a/b-binding proteins are required for stomatal response to abscisic acid in *Arabidopsis*. J. Exp. Bot..

[60] Bowles D.J. (1990). Defense-related proteins in higher plants. Annu. Rev. Biochem..

[61] Van Loon L.C., Pierpoint W.S., Boller T., Conejero V. (1994). Recommendations for naming plant pathogenesis-related proteins. Plant Mol. Biol. Rep..

[62] Wu J., Kim S.G., Kang K.Y., Kim J.G., Park S.R., Gupta R., Kim Y.H., Wang Y., Kim S.T. (2016). Overexpression of a pathogenesis-related protein 10 enhances biotic and abiotic stress tolerance in rice. Plant Pathol. J..

[63] Gachet Y., Tournier S., Lee M., Lazaris-Karatzas A., Poulton T., Bommer U.A. (1999). The growth-related, translationally controlled protein P23 has properties of a tubulin binding protein and associates transiently with microtubules during the cell cycle. J. Cell Sci..

[64] Nagano-Ito M., Ichikawa S. (2012). Biological effects of mammalian translationally controlled tumor protein (TCTP) on cell death, proliferation, and tumorigenesis. Biochem. Res. Int..

[65] Su Z., Jin S., Bernardo A., Amand P.S., Bai G. (2016). Development of High-Throughput Diagnostic Marker for Fhb1, a Major Gene for FHB Resistance in Wheat.

[66] Ambawat S., Sharma P., Yadav N.R., Yadav R.C. (2013). MYB transcription factor genes as regulators for plant responses: An overview. Physiol. Mol. Biol. Plants.

[67] Bufe A., Schramm G., Keown M.B., Schlaak M., Becker W.M. (1995). Major allergen Phl p Vb in timothy grass is a novel pollen RNase. FEBS Lett..

[68] Pontis H.G. (1978). On the scent of the riddle of sucrose. Trends Biochem. Sci..

[69] Tognetti J.A., Pontis H.G., Martinez-Noel G.M. (2013). Sucrose signaling in plants: A world yet to be explored. Plant Signal. Behav..

[70] Wind J., Smeekens S., Hanson J. (2010). Sucrose: Metabolite and signaling molecule. Phytochemistry.

[71] Grierson C., Du J.S., Zabala M.D., Beggs K., Smith C., Holdsworth M., Bevan M. (1994). Separate cis sequences and trans factors direct metabolic and developmental regulation of a potato-tuber storage protein gene. Plant J..

[72] Ishiguro S., Nakamura K. (1992). The nuclear factor SP8BF binds to the 5′-upstream regions of three different genes coding for major proteins of sweet potato tuberous roots. Plant Mol. Biol..

[73] Yadeta K.A., Elmore J.M., Creer A.Y., Feng B.M., Franco J.Y., Rufian J.S., He P., Phinney B., Coaker G. (2017). A cysteine-rich protein kinase associates with a membrane immune complex and the cysteine residues are required for cell death. Plant Physiol..

[74] Idanheimo N., Gauthier A., Salojarvi J., Siligato R., Brosche M., Kollist H., Mahonen A.P., Kangasjarvi J., Wrzaczek M. (2014). The *Arabidopsis thaliana* cysteine-rich receptor-like kinases CRK6 and CRK7 protect against apoplastic oxidative stress. Biochem. Biophys. Res. Commun..

[75] Joazeiro C.A.P., Weissman A.M. (2000). RING finger proteins: Mediators of ubiquitin ligase activity. Cell.

[76] Lorick K.L., Jensen J.P., Fang S.Y., Ong A.M., Hatakeyama S., Weissman A.M. (1999). RING fingers mediate ubiquitin-conjugating enzyme (E2)-dependent ubiquitination. Proc. Natl. Acad. Sci. USA.

[77] Laity J.H., Lee B.M., Wright P.E. (2001). Zinc finger proteins: New insights into structural and functional diversity. Curr. Opin. Struct. Biol..

[78] Amitai-Zeigerson H., Scolnik P.A., Bar-Zvi D. (1995). Tomato *Asr1* mRNA and protein are transiently expressed following salt stress, osmotic stress and treatment with abscisic acid. Plant Sci..

[79] Cakir B., Agasse A., Gaillard C., Saumonneau A., Delrot S., Atanassova R. (2003). A grape ASR protein involved in sugar and abscisic acid signaling. Plant Cell.

[80] Hu W., Huang C., Deng X.M., Zhou S.Y., Chen L.H., Li Y., Wang C., Ma Z.B., Yuan Q.Q., Wang Y. (2013). TaASR1, a transcription factor gene in wheat, confers drought stress tolerance in transgenic tobacco. Plant Cell Environ..

[81] Kim I.S., Kim Y.S., Yoon H.S. (2012). Rice ASR1 protein with reactive oxygen species scavenging and chaperone-like activities enhances acquired tolerance to abiotic stresses in *Saccharomyces cerevisiae*. Mol. Cells.

[82] Barkan A., Small I. (2014). Pentatricopeptide Repeat Proteins in Plants. Annu. Rev. Plant Biol..

[83] Klinkenberg J., Faist H., Saupe S., Lambertz S., Krischke M., Stingl N., Fekete A., Mueller M.J., Feussner I., Hedrich R. (2014). Two fatty acid desaturases, stearoyl-acyl carrier protein Δ^9^-desaturase6 and fatty acid desaturase3, are involved in drought and hypoxia stress signaling in Arabidopsis crown galls. Plant Physiol..

[84] Goyer A., Haslekas C., Miginiac-Maslow M., Klein U., Le Marechal P., Jacquot J.P., Decottignies P. (2002). Isolation and characterization of a thioredoxin-dependent peroxidase from *Chlamydomonas reinhardtii*. Eur. J. Biochem..

[85] Vogt T. (2010). Phenylpropanoid Biosynthesis. Mol. Plant.

[86] Yang S.F., Hoffman N.E. (1984). Ethylene biosynthesis and its regulation in higher plants. Annu. Rev. Plant Physiol. Plant Mol. Biol..

[87] Chen X., Steed A., Travella S., Keller B., Nicholson P. (2009). *Fusarium graminearum* exploits ethylene signalling to colonize dicotyledonous and monocotyledonous plants. New Phytol..

[88] Boyer P.D. (1993). The binding change mechanism for ATP synthase—some probabilities and possibilities. Biochim. Biophys. Acta.

[89] Kitagawa N., Mazon H., Heck A.J., Wilkens S. (2008). Stoichiometry of the peripheral stalk subunits E and G of yeast V1-ATPase determined by mass spectrometry. J. Biol. Chem..

[90] Gururani M.A., Venkatesh J., Tran L.S.P. (2015). Regulation of photosynthesis during abiotic stress-induced photoinhibition. Mol. Plant.

[91] Tameling W.I.L., Vossen J.H., Albrecht M., Lengauer T., Berden J.A., Haring M.A., Cornelissen B.J.C., Takken F.L.W. (2006). Mutations in the NB-ARC domain of I-2 that impair ATP hydrolysis cause autoactivation. Plant Physiol..

[92] Van Ooijen G., Mayr G., Kasiem M.M.A., Albrecht M., Cornelissen B.J.C., Takken F.L.W. (2008). Structure-function analysis of the NB-ARC domain of plant disease resistance proteins. J. Exp. Bot..

[93] Lam H.M., Coschigano K.T., Oliveira I.C., MeloOliveira R., Coruzzi G.M. (1996). The molecular-genetics of nitrogen assimilation into amino acids in higher plants. Annu. Rev. Plant Physiol. Plant Mol. Biol..

[94] De Gara L., de Pinto M.C., Tommasi F. (2003). The antioxidant systems vis-a-vis reactive oxygen species during plant-pathogen interaction. Plant Physiol. Biochem..

[95] Seifi H.S., Van Bockhaven J., Angenon G., Hofte M. (2013). Glutamate metabolism in plant disease and defense: Friend or foe?. Mol. Plant-Microbe Interact..

[96] von Gonner M., Schlosser E. (1993). Oxidative stress in interactions between *Avena sativa* L. and *Drechslera* spp.. Physiol. Mol. Plant Pathol..

[97] Swamy M., Sirajudeen K.N.S., Chandran G. (2009). Nitric oxide (NO), citrulline-NO cycle enzymes, glutamine synthetase, and oxidative status in kainic acid-mediated excitotoxicity in rat brain. Drug Chem. Toxicol..

[98] De Stefano M., Ferrarini A., Delledonne M. (2005). Nitric oxide functions in the plant hypersensitive disease resistance response. BMC Plant Biol..

[99] Melo P.M., Silva L.S., Ribeiro I., Seabra A.R., Carvalho H.G. (2011). Glutamine synthetase is a molecular target of nitric oxide in root nodules of *Medicago truncatula* and is regulated by tyrosine nitration. Plant Physiol..

[100] Odanaka S., Bennett A.B., Kanayama Y. (2002). Distinct physiological roles of fructokinase isozymes revealed by gene-specific suppression of Frk1 and Frk2 expression in tomato. Plant Physiol..

[101] Song N., Hu Z.R., Li Y.H., Li C., Peng F.X., Yao Y.Y., Peng H.R., Ni Z.F., Xie C.J., Sun Q.X. (2013). Overexpression of a wheat stearoyl-ACP desaturase (SACPD) gene TaSSI2 in *Arabidopsis* ssi2 mutant compromise its resistance to powdery mildew. Gene.

[102] Hurkman W.J., Tanaka C.K. (1986). Solubilization of plant membrane-proteins for analysis by two-dimensional gel-electrophoresis. Plant Physiol..

[103] Damerval C., de Vienne D., Michel Z., Hervé T. (1986). Technical improvements in two-dimensional electrophoresis increase the level of genetic variation detected in wheat-seedling proteins. Electrophoresis.

[104] Amme S., Matros A., Schlesier B., Mock H.P. (2006). Proteome analysis of cold stress response in *Arabidopsis thaliana* using DIGE-technology. J. Exp. Bot..

[105] Das A., Eldakak M., Paudel B., Kim D.W., Hemmati H., Basu C., Rohila J.S. (2016). Leaf proteome analysis reveals prospective drought and heat stress response mechanisms in soybean. BioMed Res. Int..

[106] Gupta D., Eldakak M., Rohila J.S., Basu C. (2014). Biochemical analysis of ‘kerosene tree’ *Hymenaea courbaril* L. under heat stress. Plant Signal. Behav..

[107] Hayashi G., Moro C.F., Rohila J.S., Shibato J., Kubo A., Imanaka T., Kimura S., Ozawa S., Fukutani S., Endo S. (2015). 2D-DIGE-based proteome expression changes in leaves of rice seedlings exposed to low-level gamma radiation at Iitate village, Fukushima. Plant Signal. Behav..

[108] Robbins M.L., Roy A., Wang P.H., Gaffoor I., Sekhon R.S., Buanafina M.M.D., Rohila J.S., Chopra S. (2013). Comparative proteomics analysis by DIGE and iTRAQ provides insight into the regulation of phenylpropanoids in maize. J. Proteom..

[109] Paudel B., Das A., Tran M., Boe A., Palmer N.A., Sarath G., Gonzalez-Hernandez J.L., Rushton P.J., Rohila J.S. (2016). Proteomic responses of switchgrass and prairie cordgrass to senescence. Front. Plant Sci..

[110] Poschmann G., Sitek B., Sipos B., Ulrich A., Wiese S., Stephan C., Warscheid B., Kloppel G., Vander Borght A., Ramaekers F.C.S. (2009). Identification of proteomic differences between squamous cell carcinoma of the lung and bronchial epithelium. Mol. Cell. Proteom..

[111] Skeeles L.E., Fleming J.L., Mahler K.L., Toland A.E. (2013). The impact of 3′ UTR variants on differential expression of candidate cancer susceptibility genes. PLoS ONE.

[112] Livak K.J., Schmittgen T.D. (2001). Analysis of relative gene expression data using real-time quantitative PCR and the 2^−ΔΔC^T method. Methods.

[113] Altschul S.F., Madden T.L., Schaffer A.A., Zhang J.H., Zhang Z., Miller W., Lipman D.J. (1997). Gapped BLAST and PSI-BLAST: A new generation of protein database search programs. Nucleic Acids Res..

[114] Thompson J.D., Gibson T.J., Higgins D.G. (2003). Multiple sequence alignment using ClustalW and ClustalX. Current Protocols in Bioinformatics.

[115] Szklarczyk D., Franceschini A., Kuhn M., Simonovic M., Roth A., Minguez P., Doerks T., Stark M., Muller J., Bork P. (2011). The STRING database in 2011: Functional interaction networks of proteins, globally integrated and scored. Nucleic Acids Res..

[116] Shannon P., Markiel A., Ozier O., Baliga N.S., Wang J.T., Ramage D., Amin N., Schwikowski B., Ideker T. (2003). Cytoscape: A software environment for integrated models of biomolecular interaction networks. Genome Res..

[117] Smoot M.E., Ono K., Ruscheinski J., Wang P.L., Ideker T. (2011). Cytoscape 2.8: New features for data integration and network visualization. Bioinformatics.

[118] Salavati A., Khatoon A., Nanjo Y., Komatsu S. (2012). Analysis of proteomic changes in roots of soybean seedlings during recovery after flooding. J. Proteom..

[119] Camon E., Magrane M., Barrell D., Lee V., Dimmer E., Maslen J., Binns D., Harte N., Lopez R., Apweiler R. (2004). The Gene Ontology Annotation (GOA) Database: Sharing knowledge in Uniprot with Gene Ontology. Nucleic Acids Res..

[120] De Hoon M.J.L., Imoto S., Nolan J., Miyano S. (2004). Open source clustering software. Bioinformatics.

[121] Oh I.S., Park A.R., Bae M.S., Kwon S.J., Kim Y.S., Lee J.E., Kang N.Y., Lee S.M., Cheong H., Park O.K. (2005). Secretome analysis reveals an *Arabidopsis* lipase involved in defense against *Alternaria brassicicola*. Plant Cell.

