# Supplementary material for: A Quantitative Proteomics View on the Function of Qfhb1, a Major QTL for Fusarium Head Blight Resistance in Wheat
Source: Pathogens. 2018 Jun 22;7(3):58. doi: 10.3390/pathogens7030058 (PMC6161305; doi:10.3390/pathogens7030058)
Supplement: Supplementary file 1 [file pathogens-07-00058-s001.zip › Table S1.docx]

**Supplementary Table S1**. PCR primers used for real time RT-PCR validation of expression of the genes encoding seven randomly-selected proteins from those identified by 2D-DIGE and MALDI-MS/MS analyses.

| **Protein ID** | **Protein Name** | **Primer direction** | **Primer sequence** |
| --- | --- | --- | --- |
| 67 | Eukaryotic translation initiation factor 5A1 | Forward | CCAGAATATCTACCGCCCTTG |
|  |  | Reverse | GGCAAGCCATCACCATTTATG |
| 47 | PREDICTED: uncharacterized protein LOC100822602 | Forward | GATGAAGGCGTGGAAGGAG |
|  |  | Reverse | CATGGGCTTGTCGGAGAG |
| 62 | Glutathione transferase F5 | Forward | ATGGATGCGTATGGGAATCTG |
|  |  | Reverse | AACAGATATCATGGACCCGTG |
| 57 | Chlorophyll a-b binding protein | Forward | CACCAAGAGCCCCAAGG |
|  |  | Reverse | CAAACAAGCAACCTCAGACG |
| 71 | Peroxiredoxin-2E-2 | Forward | GAGCAGTACAAGAGCATCGAG |
|  |  | Reverse | CACCTCCACCTTCTCCTTG |
| 64 | Salt tolerant protein | Forward | TGCTCATGTACCTCAACGAC |
|  |  | Reverse | TCGGGTTCACTTTCCTCTTG |
| 20 | Phosphoethanolamine methyltransferase | Forward | GAGTCCGACAAGTACAGGAAG |
|  |  | Reverse | TCGATCTTGTGCCTGTGC |
