# Supplementary material for: A Quantitative Proteomics View on the Function of Qfhb1, a Major QTL for Fusarium Head Blight Resistance in Wheat
Source: Pathogens. 2018 Jun 22;7(3):58. doi: 10.3390/pathogens7030058 (PMC6161305; doi:10.3390/pathogens7030058)
Supplement: Supplementary file 1 [file pathogens-07-00058-s001.zip › Table S2.pdf]

Supplementary Table S2. Fold changes of the proteins identified by 2D-DIGE and MALDI-MS/MS analyses within and between the resistant (fhb1+) and susceptible (fhb1-) NILs before and after Fusarium graminearum infection.

Protein ID	Annotation	The fhb1- control/ the fhb+ control	The fhb+ infected/ the fhb+control	The fhb1-infected/ the fhb+control	The fhb+infected/ the fhb1-control	The fhb1-infected/ the fhb1-control	The fhb1-infected/ the fhb1+ infected
1*	Glycosyltransferase [Triticum aestivum] / Pm3 [Triticum aestivum]	-1.71	-1.18	-1.43	1.44	1.19	-1.21
2	Ribulose bisphosphate carboxylase small chain PW9	-1.20	1.35	1.50	1.62	1.80	1.11
3*	Glycosyltransferase [Triticum aestivum] / MYB-related protein [Triticum aestivum]	1.01	1.49	1.69	1.47	1.68	1.14
4	COP9 signalosome subunit [Chlamydomonas reinhardtii]	-1.05	1.12	1.45	1.18	1.52	1.29
5	Predicted protein [Hordeum vulgare subsp. vulgare]	-1.04	1.34	1.51	1.39	1.56	1.13
6	Predicted protein [Micromonas sp. RCC299]	1.07	2.27	1.32	2.13	1.24	-1.72
7	Eukaryotic translation initiation factor 3 subunit D-like [Brachypodium distachyon], predicted	1.12	1.92	1.18	1.71	1.05	-1.62
8	Methionine synthase 1 enzyme	-1.10	1.25	1.39	1.37	1.52	1.11
9	Phenylalanine ammonia-lyase	-1.26	1.36	1.59	1.72	2.01	1.17
10	Adenosylhomocysteinase	-1.10	1.29	1.38	1.42	1.52	1.07
11	5-methyltetrahydropteroyltriglutamate-homocysteine methyltransferase-like [Brachypodium distachyon], predicted	-1.49	2.28	1.43	3.40	2.14	-1.59
12	Phenylalanine ammonia-lyase	-1.57	1.40	1.72	2.19	2.69	1.23
13*	Mechanosensitive ion channel / PPR domain containing protein / Serine/threonine protein kinase [Triticum aestivum]	-1.21	2.51	1.23	3.03	1.48	-2.05
14	Ribulose-1,5-bisphosphate carboxylase/oxygenase large subunit	-1.44	1.60	1.93	2.32	2.78	1.20
15	Ribulose-1,5-bisphosphate carboxylase/oxygenase large subunit	-1.29	1.52	1.89	1.96	2.43	1.24
16	Protein disulfide isomerase	1.74	-1.10	1.13	-1.92	-1.53	1.25
17	Protein phosphatase 2A structural subunit	1.63	-1.07	1.11	-1.75	-1.47	1.18
18	Glucose-1-phosphate adenylyltransferase large subunit, chloroplastic/amyloplastic (Fragment)	1.07	1.60	1.25	1.50	1.17	-1.28
19	Starch synthase IIa-3	1.43	1.18	2.00	-1.22	1.40	1.70
20	Phosphoethanolamine methyltransferase	1.87	1.23	1.05	-1.52	-1.78	-1.18
21	Alpha tubulin-2A	-1.34	1.31	-1.19	1.76	1.13	-1.56
22	Alpha tubulin-2A	1.10	1.32	2.11	1.20	1.92	1.60
23	Mitochondrial-processing peptidase subunit beta-like [Brachypodium distachyon], predicted	-1.36	1.24	1.23	1.68	1.66	-1.01
24	Myo-inositol-1-phosphate synthase	-2.51	1.20	1.37	3.02	3.43	1.14
25	5,10-methylene-tetrahydrofolate reductase	-1.16	1.18	1.39	1.37	1.61	1.17
26	Atp1	-1.13	1.29	1.46	1.46	1.65	1.13
27	Phosphoglucomutase	-1.12	1.40	1.54	1.56	1.72	1.10
28	Phosphoglucomutase	-1.03	1.36	1.54	1.41	1.60	1.13
29	Phosphoglucomutase	-1.03	1.27	1.57	1.32	1.62	1.23
30	Ribulose-1,5-bisphosphate carboxylase/oxygenase large subunit	-1.35	-1.12	-1.54	1.21	-1.14	-1.37
31	Heat shock protein HSP26	1.11	1.22	1.52	1.10	1.37	1.25
32	Plastid glutamine synthetase 2	-1.05	-1.26	-1.51	-1.20	-1.44	-1.20
33	Predicted protein [Hordeum vulgare subsp. vulgare]	1.22	-1.76	-1.63	-2.14	-1.99	1.08
34	Glutamine synthetase isoform GSr1	1.45	-1.06	-1.10	-1.53	-1.59	-1.04
35	Actin [Triticum aestivum]	-1.44	1.63	1.55	2.34	2.23	-1.05
36	S-adenosylmethionine synthase	-1.20	1.26	1.32	1.52	1.60	1.05
37	S-adenosylmethionine synthase	-1.49	1.32	1.36	1.97	2.03	1.03
38	S-adenosylmethionine synthase	-1.44	1.40	1.61	2.02	2.32	1.15
39	Caffeic acid 3-O-methyltransferase	-1.33	1.06	1.23	1.41	1.64	1.16
40	Caffeic acid 3-O-methyltransferase	-1.36	1.10	1.26	1.50	1.72	1.15
41	Fructokinase-1-like [Brachypodium distachyon], predicted	-1.07	1.43	1.23	1.53	1.32	-1.16
42	Os08g0130500 [Oryza sativa Japonica Group]	-1.36	1.46	1.29	1.98	1.76	-1.13
43	Stearoyl-ACP desaturase [Triticum aestivum]	-1.29	-1.11	-1.84	1.17	-1.42	-1.66
44	Uncharacterized protein LOC100273160 [Zea mays]	-1.03	1.65	1.29	1.69	1.33	-1.27
45	Malate dehydrogenase 1, mitochondrial-like [Brachypodium distachyon], predicted	-1.23	1.04	1.25	1.28	1.54	1.20
46	Isopentenyl pyrophosphate isomerase	1.07	-1.41	-1.26	-1.51	-1.36	1.11
47	Uncharacterized protein LOC100822602 [Brachypodium distachyon], predicted	-1.86	-1.33	-1.52	1.40	1.23	-1.14
48	NADPH: isoflavone reductase [Glycine max]	-1.40	1.09	-1.00	1.53	1.39	-1.10
49*	Root phototropism 2 [Oryza sativa Japonica Group], putative, expressed	-1.27	-1.59	-1.49	-1.25	-1.17	1.07
50	Group 5/9 grass pollen allergen R8-5 [Triticum turgidum subsp. durum x Secale cereale]	-1.37	1.69	1.41	2.31	1.93	-1.20
51	Chlorophyll a-b binding protein of LHCII type III, chloroplastic-like [Brachypodium distachyon], predicted	1.01	-1.39	-1.49	-1.41	-1.51	-1.07
52	Vacuolar invertase	-1.87	1.11	-1.15	2.07	1.62	-1.27
53	Translationally-controlled tumor protein	1.02	-1.65	-1.92	-1.69	-1.96	-1.16
54	Predicted protein [Hordeum vulgare subsp. vulgare]	-1.03	-1.55	-1.71	-1.50	-1.66	-1.11
55	Chloroplast light-harvesting chlorophyll a/b binding protein	1.01	-1.50	-1.65	-1.52	-1.67	-1.10
56	Adenylate kinase, chloroplastic-like	1.15	-1.12	-1.34	-1.29	-1.54	-1.19
57	Chlorophyll a-b binding protein	1.12	1.03	-1.60	-1.09	-1.79	-1.64
58	Chlorophyll a-b binding protein CP24 10A, chloroplastic [Glycine max], predicted	1.04	-1.53	-1.67	-1.60	-1.74	-1.09
59	Chlorophyll a-b binding protein 8 [Zea mays]	1.05	-1.22	-1.60	-1.28	-1.67	-1.31
60	bZIP transcription factor	-1.11	1.38	1.29	1.53	1.43	-1.07
61	Oxygen-evolving enhancer protein 2, chloroplastic	-1.05	-1.15	-1.63	-1.09	-1.55	-1.42
62	Glutathione transferase F5	1.57	1.08	-1.01	-1.46	-1.58	-1.08
63*	P450 / Abscisic acid-induced protein [Triticum aestivum] / Mlo protein [Triticum aestivum]	1.31	-1.19	1.02	-1.56	-1.28	1.21
64	Salt tolerant protein	2.88	1.09	1.02	-2.63	-2.84	-1.08
65	MYB-related protein	1.11	-1.88	-1.86	-2.07	-2.06	1.01
66	Thioredoxin-dependent peroxidase [Leymus chinensis]	1.10	-1.07	-1.47	-1.17	-1.62	-1.38
67	Eukaryotic translation initiation factor 5A1	-1.85	1.14	-1.97	2.11	-1.06	-2.24
68	Eukaryotic translation initiation factor 5A1	1.29	-1.05	-1.29	-1.36	-1.66	-1.22
69	Cytochrome b6-f complex iron-sulfur subunit	-2.19	-1.10	-2.31	2.00	-1.05	-2.10
70	ASR1 protein [Solanum peruvianum]	-1.25	-1.52	-1.63	-1.22	-1.31	-1.07
71	Peroxiredoxin-2E-2, chloroplastic-like [Brachypodium distachyon], predicted	1.20	1.15	-1.32	-1.04	-1.59	-1.52
72	Pathogenesis related protein 10	1.05	2.22	2.15	2.11	2.04	-1.03
73	Thioredoxin M-type	1.16	-1.24	-1.58	-1.44	-1.83	-1.27
74	Ribulose bisphosphate carboxylase small chain clone 512 (Fragment)	1.13	-1.30	-1.41	-1.47	-1.59	-1.08
75	Ribulose-1,5-bisphosphate carboxylase/oxygenase small subunit	-1.33	-1.15	-1.68	1.15	-1.26	-1.45
76	Profilin	-1.19	1.60	1.58	1.91	1.88	-1.02
77	Thylakoid lumenal protein 1 [Vitis vinifera], predicted	1.14	-1.28	-1.64	-1.46	-1.86	-1.27
78	Vacuolar ATPase subunit F [Triticum aestivum]	1.04	-1.40	-1.67	-1.45	-1.74	-1.20
79	Ribulose bisphosphate carboxylase small chain clone 512	-1.71	1.05	-1.68	1.79	1.02	-1.76
80*	60S ribosomal protein L5 / Ent-kaurene synthase like 1 / Thylakoid-bound ascorbate peroxidase	-6.87	3.53	2.85	24.26	19.54	-1.24

* Protein with no confidence; Red indicates differentially accumulated proteins.
