# Supplementary material for: A Quantitative Proteomics View on the Function of Qfhb1, a Major QTL for Fusarium Head Blight Resistance in Wheat
Source: Pathogens. 2018 Jun 22;7(3):58. doi: 10.3390/pathogens7030058 (PMC6161305; doi:10.3390/pathogens7030058)
Supplement: Supplementary file 1 [file pathogens-07-00058-s001.zip › Table S3.pdf]

Supplementary Table S1. Differenrially accumulated proteins identified by 2D-DIGE and MALDI-MS/MS analyses and their top ten functional annotations

ID	Rank	Top 10 Annotations	Sequence	Protein MW	Protein PI	Rep Coint	Protein Score	Protein C.I.%	Total Ion Score	Total Ion C.I.%
1		MIKAGERPNSKLLRGGGRQESRRFRMLAIVVGFFIVSLTFVLVSKPDAILFGLNGKLPADQAPASILIQQKVNTPAQKTSTDAIIGGDPKVVDDETYVKPKEIRGGEEEDHRVLSEPDPASGMAEPAANKDGDARKSSDETLGGERKDEAEEERDEEKHAKVTLPTVSNYTIHDAEDAENAKQEGLSLSNVQHEQQQQQGSKPLCDFSNFRANVCEMRGDVRVHPKATSVLFMEPEGSQRDEVWKIKPYPRKGDEFCLSHITELTVKSSKVAAECTRYHDVPVVIFSLTGYTGNLFHDFTDVIVPLFTTAAQFDGEVQFLITDMALWWTVKYHTLLQKLSKYPLIDFSKDDQVRCFKHAIVGTHAYMEFTIDAAKSPNGVTMVDFNRFMRAAYSLPKAAAAALGESPRVKPRLLIIKRHRTRMFLNLEEIIGMAEELGFEVVIDEANVSSDINGFAKLVNSVDVMMGVHGAGLTNCVFLPQNATLIQIVPFGGLDWISRTDFGNPSEMMGLRYKQYAITVDESSLTDHYPRDHKIFKDPISFHKRGFEFIRRTFMDKQNVRLDCKRFRPVLLEALDNLNQ								
	1	Glycosyltransferase [Triticum aestivum]	gi|56409844	65081	5.95	9	32	0		
	2	Leucyl-tRNA synthetase [Triticum aestivum], putative, expressed	gi|300681548	108946	5.96	13	31	0		
	3	Pm3 [Triticum aestivum]	gi|296280022	159265.2	6.03	14	23	0		
	4	ATPase-like zinc transporter [Triticum aestivum], putative	gi|95114390	110389.1	6.58	11	23	0		
	5	Powdery mildew resistance protein PM3C [Triticum aestivum]	gi|82492377	159009.1	6.18	12	22	0	141	100
	6	Pm3 [Triticum aestivum]	gi|296280016	158971	6.07	13	22	0	56	99
	7	RecName: Full=Alpha-2-purothionin; Contains:	gi|401184	14547.8	5.13	5	21	0		
	8	Ribosomal protein L16 [Triticum aestivum]	gi|254998340	10715.8	10.64	3	20	0		
	9	Powdery mildew resistance protein PM3 variant	gi|225580379	158875	6.18	12	20	0		
	10	Pm3 [Triticum aestivum]	gi|296280024	159020	6.07	12	20	0		
2		MAPAVMASSATTVAPFQGLKSTAGLPISCRSGSTGLSSVSNGGRIRCMQVWPIEGIKKFETLSYLPPLSTEALLKQVDYLIRSKWVPCLEFSKVGFVFREHNSSPGYYDGRYWTMWKLPMFGCTDATQVLNEVEEVKKEYPDAYVRVIGFDNMRQVQCVSFIAFRPPGCEESGKA								
	1	Ribulose bisphosphate carboxylase small chain PW9 [Triticum aestivum]	RBS2_WHEAT	19441.7	8.52	7	39	95.506		
	2	Alpha-2-purothionin [Triticum aestivum]	THN2_WHEAT	14547.8	5.13	6	32	78.978	7	84.896
	3	Ribulose bisphosphate carboxylase small chain clone	RBS3_WHEAT	13046.5	5.84	4	22	0		
	4	Gibberellin 3-beta-dioxygenase 2-1 [Triticum aestivum]	G3O21_WHEAT	40219.2	6.43	6	21	0		
	5	Bowman-Birk type proteinase inhibitor I-2B (Fragment) [Triticum aestivum]	IBB1_WHEAT	6218.9	8.67	4	19	0		
	6	DNA-directed RNA polymerase subunit beta [Triticum aestivum]	RPOB_WHEAT	121575.7	8.91	9	17	0	5	75.896
	7	Gibberellin 3-beta-dioxygenase 2-2 [Triticum aestivum]	G3O22_WHEAT	40329.4	6.73	5	16	0		
	8	Gibberellin 3-beta-dioxygenase 2-3 [Triticum aestivum]	G3O23_WHEAT	40300.4	6.43	5	16	0		
	9	Ribulose bisphosphate carboxylase small chain [Triticum aestivum]	RBS1_WHEAT	19404.8	8.99	4	14	0		
	10	Ninja-family protein 2 [Triticum aestivum]	NNJA2_WHEAT	33805.9	9.51	4	14	0		
3		MIKAGERPNSKLLRGGGRQESRRFRMLAIVVGFFIVSLTFVLVSKPDAILFGLNGKLPADQAPASILIQQKVNTPAQKTSTDAIIGGDPKVVDDETYVKPKEIRGGEEEDHRVLSEPDPASGMAEPAANKDGDARKSSDETLGGERKDEAEEERDEEKHAKVTLPTVSNYTIHDAEDAENAKQEGLSLSNVQHEQQQQQGSKPLCDFSNFRANVCEMRGDVRVHPKATSVLFMEPEGSQRDEVWKIKPYPRKGDEFCLSHITELTVKSSKVAAECTRYHDVPVVIFSLTGYTGNLFHDFTDVIVPLFTTAAQFDGEVQFLITDMALWWTVKYHTLLQKLSKYPLIDFSKDDQVRCFKHAIVGTHAYMEFTIDAAKSPNGVTMVDFNRFMRAAYSLPKAAAAALGESPRVKPRLLIIKRHRTRMFLNLEEIIGMAEELGFEVVIDEANVSSDINGFAKLVNSVDVMMGVHGAGLTNCVFLPQNATLIQIVPFGGLDWISRTDFGNPSEMMGLRYKQYAITVDESSLTDHYPRDHKIFKDPISFHKRGFEFIRRTFMDKQNVRLDCKRFRPVLLEALDNLNQ								
	1	Glycosyltransferase [Triticum aestivum]	gi|56409844	65081	5.95	9	30	0		
	2	MYB-related protein [Triticum aestivum]	gi|359952782	48051.4	8.93	8	29	0		
	3	Ribulose bisphosphate carboxylase small chain clone 512	gi|132107	13046.5	5.84	4	23	0		
	4	YABBY2 [Triticum aestivum]	gi|158822026	20986.4	9.42	5	22	0		
	5	Ribulose-bisphosphate carboxylase small chain precursor - wheat (fragment)	gi|82619	15344.9	8.95	4	22	0		
	6	Transcription factor [Triticum aestivum], putative	gi|157272093	48867.5	9.34	9	22	0		
	7	Ribulosebisphosphate carboxylase [Triticum aestivum]	gi|755804	18119.9	8.95	4	22	0		
	8	Putative protein [Triticum aestivum]	gi|283099379	57999.7	5.26	7	21	0		
	9	Bowman-Birk trypsin inhibitor-like protein [Triticum aestivum]	gi|162319710	9856.6	8.67	2	20	0	12	0
	10	Peroxidase 66 precursor, putative [Triticum aestivum], expressed	gi|300681482	35689.8	5.72	6	20	0		
4		MEDYGFEYSDEDPEEEDVDIENQYYNAKGMLEGDDPREAIDGFKQVVEMEQEKGEWGFKSLKQLVKLHYKLGQYAKMMESYRLMLSYAEGSTITKNASEKKINSLLDFMGGASDMQVLQEFYEATLKALEKAKNERLWFKTQLKLANLWFKKQEYGRMAKIIRELHKSCQKEDGSEDPKKGTQLLDIYALEIQMATEQRNNKKLKKLYQQALTVKSAIPHPRIMGIIRECGGKMHMHDELWSEAATDFFEAFKAYDEAGVGRRVQCLKYLVLANMLMESRVDPFDAQEAKPYKQDPEIVSMTNLVAAFQRNDINDFEKILRTNKRTIMDDPFIRNYVEDLLRKIRTQVLLKIIQPYTRVRIPFIAQKLNIPAPDVEQLLVSLILDGRVAGNIDQVNQILEVGSRQEGARKYTALDKWAAQIGSLHSAVQNKLVMNTA								

	1	COP9 signalosome subunit [Chlamydomonas reinhardtii]	gi|159463648	50437.8	6.03	16	72	92.684		
	2	Hypothetical protein OsI_02271 [Oryza sativa Indica groip]	gi|218188383	14361.2	6.99	7	63	47.002	11	0
	3	Maturase K [Jacqueshuberia brevipes]	gi|183529139	59996.3	9.28	13	61	21.61		
	4	PREDICTED: DEAD-box ATP-dependent RNA helicase 36-like [Glycine max]	gi|356528665	66719.2	9.12	14	61	16.003		
	5	RNA polymerase beta subunit (chloroplast) [Olea europaea]	gi|283794960	120754.7	8.23	16	56	0	13	0
	6	PREDICTED: pentatricopeptide repeat-containing protein At4g37170-like [Glycine max]	gi|356547226	78764.6	8.02	15	54	0		
	7	Minichromosome maintenance protein 4 [Volvox carteri f. nagariensis]	gi|302845553	108094.9	6.11	8	54	0	37	27.737
	8	Chloroplast Mg-chelatase subunit XANTHA-G precursor [Hordeum vulgare]	gi|71493349	82619.1	5.52	15	53	0		
	9	Maturase-like protein [Leucaena retusa]	gi|37518612	59801.3	9.41	12	52	0		
	10	Unknown protein; 70672-76070 [Arabidopsis thaliana]	gi|12324587	104123.1	4.79	17	52	0		
5	MDLPKEIFLKDYKKPDYLFDTVDLEFQLGDEKTIVTSNIAVSPGNEGTSSPLALNGRDLKLLSIKINGTELKSDKYTVDPRHLTILTPPAGVFNMEIVTEIYPQLNTSLEGLYRSTGNFCTQCEAEGFRKITYFQD RPDVMAKYTCRIEGDKTLYPVLLSNGNLIEQGDLEGGKHYALWEDPFKKPCYLFALVAGQLECREDSFVTCSGRKVTLRIWTPAQDLPKTSHAMYSLKEAMKWDEEVFGLEYDLDLFNIVVVPDFNMGAM ENKSLNVFQSRLVLASPEAATDGDYAAILGVIGHEYFHNWTGNRVTCRDWFQLTLKEGLTVFRDQEFSSDLGCRTVKRIADVSKLRSYQFPQDAGPMAHPIRPLSYIKMDNFYTVTVYEKGAEVVRMYKTM LGASGFRKGMDLYFQRHDGQAVTCEDFYAAMCDANNAQLPNFLQWYSQAGTPTVKATSSYDEGSQTFSLKLSQEVPPTPGQPMKEPMFIPVAVGLVDSTGEDMPLTSIYSDGMVQTLSNDGHPVFTTVLQF KKTEEEFIFNNVPERPVPSLLRGYSAPIRLDSDLTESDLYFLLANDSDEFNRWEAGQILARKLMFSLVADFQQQKTLALNTKFVDGLRAILRSTSLDKEFIAKAITLPGQGEIMDMMSIADPDAVHAVRTFIKKE LAFQLKDDLLAAVTSNRSSEAYVFNHDSVARRALKNTCLAYLASLNEPDVTELALNEYKSATNMTEQFAALAALSQNPGQVREDALLDFYNKWQQDYLVVSKWFALQATSDIPGNVANVRKLLAHPAFDM [illegible]									
	1	Predicted protein [Hordeum vulgare subsp. vulgare]	gi|326503600	99426.8	5.17	17	204	100	141	100
	2	PREDICTED: pentatricopeptide repeat-containing protein At4g37170-like [Glycine max]	gi|356547226	78764.6	8.02	12	66	70.875	30	0
	3	Heat shock protein 70, mitochondrial precursor [Micromonas pusilla CCMP1545]	gi|303275878	78601.4	6.08	7	62	31.725	49	94.836
	4	Katanin p80 WD40 repeat-containing subunit B1 homolog 1-like [Brachypodium distachyon], predicted	gi|357162659	103217.7	8.24	16	59	0	11	0
	5	Hypothetical protein VOLCADRAFT_120430 [Volvox carteri f. nagariensis]	gi|302831071	48086.5	6.82	9	56	0	22.00	0
	6	Hypothetical protein MTR_5g089990 [Medicago truncatula]	gi|357494001	9860.8	6.03	7	54	0		
	7	Hypothetical protein VITISV_020021 [Vitis vinifera]	gi|147787791	116094.5	9.51	17	53	0		
	8	Maturase K	gi|68052514	59985.5	9.47	12	53	0		
	9	Predicted protein [Chlamydomonas reinhardtii]	gi|159485182	23517.2	11.18	9	50	0		
	10	Predicted protein [Physcomitrella patens subsp. patens]	gi|168058449	35660.3	8.54	10	48	0		
6	MASSAPSRRAAASLLASLRAVTPATRCPNPAVPSTRAFDRRGFASWASTPSPASGVAWMTSRVKELKDAARAQMDAAKVAPERLKSAARESVRRRLASALGPRAAESIMSGTLSLNPATHARNLTLRARAFR FRHRAPIAAVAGAGATCATYGAAVGAAERLRGRGDDDAQRLDATSKLATLALSAGCVAAAATYLRARSRVDPERVHAAVMRRLERHPGLCEVLGAPLVSSEHRAVVTTGGVWTSRTYTPRFRDAKVHMAF RVAGTRKLGLVTVEAKKRKAWGGGVRASWHHFIGGDPHEYALVAVDVAADNGDEHRVYLAGGSERYAKQGEVTGMHMREALVSVASEAYEAEQRGEEAEEDERLRREKAEARAESAPKPLDKGGGMWP AERVTDYVAQKRHEAARFVTKLGVADARK									
	1	Predicted protein [Micromonas sp. RCC299]	gi|255083775	44954.3	10.40	4	66	71.538	56	99.166
	2	Intron-binding protein aquarius [Brachypodium distachyon], predicted	gi|357112007	179208.8	5.47	22	57	0		
	3	Hypothetical protein ARALYDRAFT_894344 [Arabidopsis lyrata subsp. lyrata]	gi|297841293	33755.1	8.19	11	54	0		
	4	RNA polymerase beta' subunit protein [Meliosma aff. cuneifolia Moore 333]	gi|290489566	80834.2	9.00	16	54	0		
	5	Unnamed protein product [Ostreococcus tauri]	gi|308799307	33417.7	6.09	12	51	0		
	6	E3 ubiquitin-protein ligase PUB23-like [Glycine max], predicted	gi|356543678	45164	8.81	11	49.00	0		
	7	Predicted protein [Chlamydomonas reinhardtii]	gi|159468908	22756.3	6.22	9	47.00	0		
	8	Predicted protein [Ostreococcus lucimarinus CCE9901]	gi|145341341	148305.1	5.65	24	47.00	0		
	9	Predicted protein [Micromonas sp. RCC299]	gi|255089182	60494	7.75	12	46.00	0		
	10	Unnamed protein product [Vitis vinifera]	gi|296090673	11253.8	11.94	7	46.00	0		
7	MVRHIQQTCGMNTVQTLTIIYENNAACVAQVQTGYVKSNLTEHINPKFFYAHELQKMNEDLDATSEPDSWQELHNKAQSRPDSSPKAPFSSFRIANPNRTLAPPLPPPSGSKPPPTATMGFDVGVVPFNPDGW GPPETPAAPPSLGGATATASIPFAPFSRSDKLGRIADWTRNPGGPGMHPAASRDSVFDFTSADDSLAAAAEDSSFHLVDAKPPPRHPRFGPKWRFNQRPQLPQRRDEEVEAKRREAEKERARRERHYQNHRSH HHQGFRGNQSSSAKPSVDIQPDWTMREQIPFANFTKLSFTVNDQPEDLLVCGAVEFYDRAYDRVNPKAARRLERFKSRNFFKVTTTDDPVIRRLAEEDKATVFATDAILAALMCTPRSILSWDIVVQRVGNKL FFDKRDGSQLDLLTVNETAQEQLPENKDDINSAHALAVEATYINQNFSQQVLVHEGEKVTFDEPNPFASEGEDAASNGYRYRRWKLDEEISIIARCEVHAASADASGGRQFFTLNALNEFDPKITGVDWRQK LETQRGAVLATELKNNANKLARWTCQALLSGADMMKLGYVSRVHPRDHYNHSILTVMGYKPRDFAAQINLNTANMWGIVKSIVDICMKFEEGKYVLVKDPAKPQVRIYSVPNDAFENDYVEEPLPEEEQV [illegible]									
	1	Predicted protein [Hordeum vulgare subsp. vulgare]	gi|326533418	67502.8	5.52	21	296	100	161	100
	2	Eukaryotic translation initiation factor 3 subunit D-like [Brachypodium distachyon], predicted	gi|357128487	78267.8	5.67	11	148	100	109	100

7	3	Uncharacterized protein LOC100383775 [Zea mays]	gi|293336540	36090.9	4.80	4	119	100	109	100
	4	Hypothetical protein SELMODRAFT_425252 [Selaginella moellendorffii]	gi|302809182	30026.3	9.81	11	107	99.998	48	93.769
	5	Unknown [Glycine max]	gi|255634452	20542.2	4.54	3	73	95.277	62	99.72
	6	Os05g0566500 [Oryza sativa Japonica Group]	gi|297604927	65040.4	5.30	12	72	92.684		
	7	30S ribosomal protein S1, chloroplastic;	gi|133872	44759.1	5.41	8	71	92.339		
	8	Uncharacterized protein ycf45-like [Brachypodium distachyon], predicted	gi|357125252	71118.1	5.54	5	70	89.666	61	99.695
	9	Spermidine synthase 2-like [Glycine max], predicted	gi|356567616	36810.3	4.80	3	69	88.135	62	99.72
	10	F-box protein SKIP23 [Medicago truncatula]	gi|357492719	33514.1	8.44	3	68	85.403	60	99.562
8	MASHIVGYPRMGPKRELKFALESFWDGKSSAEDLEKVATDLRASIWKQMAGAGIKYIPSNTFAYYDQVLDTTAMLGAVPDRYSWTGGEINLSTYFSMARGNATVPAMEMTKWFDTNYHFIVPELGPDTKF<br>SYSSHKAINEYKEAKALGVDTVPVLVGPVSYLLLSKAAKGVEKSFSPLSLLSSILPVYKEVIAELKAAGASWIQFDEPTLVKDLESHQLAAFSAAYTELESTLSGLNVLVETYFADVPAESYKTLTSLSSVTAYG<br>FDLERGTKTLELVKSGFPAGKYLFAGVVDGRNIWADDLAASLATLQSLEAIVGKDKLVVSTSCSLMHTAVDLVNETKLDDEIKSWLAFAAQKVVEVNALAKALAGQKDEAYFAANAAALASRRSSPRVTNE<br>EVQKAATALKGSDHRRATTVSARLDAQQKKLNLPVLPTTTIGSFPQTVELRRVRREYKAKKISEEEYTNAIKEEISKVVKIQEELDIDVLVHGEPERNDMVEYFGEQLSGFTFTANGWVQSYGSRCVKPPIIYG<br>DVSRPNPMTVFWSKMAQSMTARPMKGMLTGPVTILNWSFVRNDQPRFETCYQIALAIKKEVEDLEAGGIQVIQIDEAALREGLPLRKSEHAFYLDWAVHSFRITNCGVQDTTQIHTHMCYSNFNDIIQSIINM<br>DADVITIENSRSDEKLLSVFREGVVYGAGIGPGVYDIHSPRIPSKEEIADRVNKMLAVLDTNILWVNPDCGLKTRKYAEVKPALTNMVEAAKQIRAELAKAQ									
	1	Methionine synthase 1 enzyme [Hordeum vulgare subsp. vulgare]	gi|68655495	84511.3	5.74	27	1,100	100	928	100
	2	Cobalamin-independent methionine synthase [Triticum monococcum]	gi|115589740	26146.4	6.10	11	622	100	546	100
	3	5-methyltetrahydropteroyltriglutamate-homocysteine methyltransferase-like [Brachypodium distachyon], predicted	gi|357155679	84641.5	5.97	21	573	100	468	100
	4	Os12g0623900 [Oryza sativa Japonica Group]	gi|115489652	84531.5	5.93	19	535	100	448	100
	5	5-methyltetrahydropteroyltriglutamate-homocysteine methyltransferase, putative, expressed [Oryza sativa Japonica Group]	gi|108862992	84582.4	5.93	18	529	100	451	100
	6	Predicted protein [Hordeum vulgare subsp. vulgare]	gi|326513870	84376.2	5.82	18	528	100	449	100
	7	5-methyltetrahydropteroyltriglutamate-homocysteine methyltransferase, putative, expressed [Oryza sativa Japonica Group]	gi|108862990	78929.9	7.19	17	522	100	448	100
	8	Os12g0624000 [Oryza sativa Japonica Group]	gi|115489654	84612.4	5.93	17	521	100	451	100
	9	5-methyltetrahydropteroyltriglutamate--homocysteine methyltransferase-like [Brachypodium distachyon], predicted	gi|357119807	84451.3	6.05	16	513	100	449	100
	10	LOC100286153 [Zea mays]	gi|226531522	84417.3	5.85	16	441	100	379	100
9	MACAWRSRSRADPLNWGKAAEELSGSHLEAVKRMVEEYRKPVVTMEGATTIAMVAAVAAGSDTRVELDESARGRVKESSDWVMNSMMNGTDSYGVTTGFGATSHRRTKEGGALQRELIRFLNAGAFGTGTDGHVLPAAA<br>TRAAMLVRVNTLLQGYSGIRFEILETIATLLNANVTPCLPLRGTITASGDLVPLSYIAGLVTGRPNSMATAPDGSKVNAAEAFKIAGIQHGFFELQPKEGLAMVNGTAVGSGLASMVLFEANVLSLLAEVLSGVFCEVMNGKPEF<br>TDHLTHKLKHHPGQIEAAAIMEHILEGSSYMMLAKKLGELDPLMKPKQDRYALRTSPQWLGPQIEVIRAATKSIEREINSVNDNPLIDVSRGKAIHGGNFQGTPIGVSMDNTRLAIAAIGKLMFAQFSELVNDFYNNGLPSNLSG<br>GRNPSLDYGFKGAEIAMASYCSELQFLGNPVTNHVQSAEQHNQDVNSLGLISSRKTAEAIDILKLMSSTFLVALCQAIDLRHLEENVKNAVKSCVKTVARKTLSTDNNGHLHNARFCEKDLLLTIDREAVFAYADDPCSANYPL<br>MQKMRAVLVEHALANGEAEAHVETSVFAKLAMFEQELRAVLPKEVEAARSAVENGTAAQQNRIAECRSYPLYRFVRKELGTEYLTGEKTRSPGEEVDKVFVAMNQGKHIDALLECLKEWNGEPLPLC									
	1	Phenylalanine ammonia-lyase	gi|3024363	75889.5	6.10	10	120	100	88	100
	2	phenylalanine ammonia-lyase [Triticum aestivum]	gi|371928984	55174.2	5.88	4	97	100	88	100
	3	Inducible phenylalanine ammonia-lyase [Triticum	gi|9965440	59360.4	5.88	4	96	100	88	100
	4	Kinase R-like protein [Triticum aestivum]	gi|17981551	18167.4	7.12	5	28	0		
	5	Eukaryotic translation initiation factor [Triticum aestivum],putative, expressed	gi|300681560	117082.5	9.36	12	26	0		
	6	Glucan water dikinase [Triticum aestivum]	gi|295687224	112709.9	4.91	11	24	0		
	7	Chloroplast acetyl-CoA carboxylase [Triticum aestivum]	gi|55668382	8203.9	4.39	3	24	0		
	8	WRKY transcription factor [Triticum aestivum]	gi|125491389	34615	10.09	6	23	0		
	9	Flavanone 3-hydroxylase [Triticum aestivum]	gi|71361211	17494.9	8.83	4	23	0		
	10	Os07g0450000 [Oryza sativa Japonica Group]	gi|115471897	12130.5	10.25	5	23	0		
10	MALSVEKTSSGREYKVKDLFQADFGRLELELAEVEMPGLMACRTEFGPSQPFKGARISGSLHMTIQTAVLIETLTALGAEVRWCSCNIFSSQDHAAAAIARDSAAVFAWKGETLEEYWWCTERCLDWGVGGGPDLIVDDGGD<br>ATLLIHEGVKAEEEFEKSGKVPDPESTDNPEFKIVLTIIRDGLKTDASKYRKMKERLVGVSEETTTGVKRLYQMQESGTLLFPAINVNDSVTKSKFDNLYGCRHSLPDGLMRATDVMIAGKVAVVCGYGDVGKGCAAALKQAG<br>ARVIVTEIDPICALQALMEGIQILTLEDVVSEADIFVTTTGNKDIIMVDHMRKMKNNAIVCNIGHFDNEIDMNGLETYPGVKRITIKPQTDRWVFPETKTGIIVLAEGRLMNLGCATGHPSFVMSCSFTNQVIAQLELWNEKASG<br>KYEKKVYVLPKHLDEKVAALHLGKLGARLTKLTKSQSDYISIPIEGPYKLRLYRY									
	1	Adenosylhomocysteinase [Triticum aestivum]	SAHH_WHEAT	53402.1	5.65	4	26	8.237	17	98.718
	2	Ribulose bisphosphate carboxylase small chain clone 512 (Fragment) [Triticum aestivum]	RBS3_WHEAT	13046.5	5.84	4	21	0		
	3	NAD(P)H-quinone oxidoreductase subunit 4L, chloroplastic [Triticum aestivum]	NU4LC_WHEAT	11296.1	9.09	3	20	0		
	4	Alpha-1-purothionin (Fragment) [Triticum aestivum]	THN1_WHEAT	13516.2	4.76	2	19	0	10	93.47
	5	Glutathione S-transferase OS=Triticum aestivum	GSTZ_WHEAT	23737.3	6.06	4	17	0		
	6	Phosphoglycerate kinase, cytosolic OS=Triticum	PGKY_WHEAT	42095.7	5.64	3	16	0	8	90.561
	7	Gibberellin 3-beta-dioxygenase 2-3 [Triticum aestivum]	G3O23_WHEAT	40300.4	6.43	5	15	0		

	8	Alpha-2-purothionin [Triticum aestivum]	THN2_WHEAT	14547.8	5.13	4	15	0		
	9	DNA-directed RNA polymerase subunit beta [Triticum aestivum]	RPOC2_WHEAT	169939	6.50	11	15	0	1	46.678
	10	Small heat shock protein, chloroplastic [Triticum aestivum]	HS21C_WHEAT	26579.5	9.64	4	14	0		
11	MASHIVGYPRMGPKRELKFALESFWDGKSSAEDLEKVATDLRASIWKQMATTGIKYIPSNTFSYYDQVLDTTAMLGAVPDRYSWTGGEINLSTYFSMARGNATVPAMEMTKWFDTNYHFIVPELGPNTKFSYSSHKAINEYKE<br>AKALGIDTVPVLVGPVSYLLLSKPAKGVEKSFSLLSLLSSILPVYKEVIAELKAAGASWIQFDEPTLVKDLDSHQLAAFSSAYTELESALSGLNVLVETYFADVPAESYKTLTSLSSVTAYGFDLERGTKTLDLVRSGFPAGKYLFA<br>GVVDGRNIWADDLAASLITLQSLEAVVGKDKLVVSTSCSLMHTAVDLVNETKLDEEIKSWLAFAAQKVVEVNALAKALAGQKDEAYFAANAAAQSSRRSSPRVTNAEVQKAATALKGSDHRRATNVSARLDAQQKKLNLPI<br>LPTTTIGSFPQTVELRRVRREYKAKKISEEEYTNAIKEEISKVVKIQEELDIDVLVHGEPERNDMVEYFGEQLSGFAFTANGWVQSYGSRCVKPPIIYGDVSRPNPMTVFWSKTAQSMTARPMKGMLTGPVTILNWSFVRNDQPR<br>FETCYQIALAIKKEVEDLEAGGIQVIQIDEAALREGLPLRKAEHAFYLDWAVHSFRITNCGVQDTTQIHTHMCYSNFNDIIQSIINMDADVITIENSRSDEKLLSVFREGVVYGAGIGPGVYDIHSPRIPSMEEIADRVNKMLAVLD<br>TNILWVNPDCGLKTRKYTEVKPALANMVEAAKLIRKQLASTK									
	1	5-methyltetrahydropteroyltriglutamate-homocysteine methyltransferase-like [Brachypodium distachyon]	gi|357155679	84641.5	5.97	10	161	100	140	100
	2	Methionine synthase 1 enzyme [Hordeum vulgare subsp. vulgare]	gi|68655495	84511.3	5.74	10	161	100	140	100
	3	Cobalamin-independent methionine synthase [Triticum monococcum]	gi|115589740	26146.4	6.10	5	160	100	140	100
	4	Methionine synthase [Medicago truncatula]	gi|357508777	88795	6.16	9	157	100	140	100
	5	Methionine synthase [Medicago truncatula]	gi|357508781	83133.1	5.87	7	151	100	140	100
	6	Alcohol dehydrogenase [Gossypium thurberi]	gi|6017997	23597.9	6.58	9	66	74.633		
	7	Hypothetical protein OsJ_17627 [Oryza sativa Japonica Group]	gi|222630692	8358.2	5.00	6	51	0		
	8	Predicted protein [Micromonas sp. RCC299]	gi|255076057	172367	7.09	17	51	0		
	9	Putative protein [Arabidopsis thaliana]	gi|3297818	73366.5	6.22	14	51	0		
	10	Hypothetical protein CHLNCDRAFT_55969 [Chlorella variabilis]	gi|307110782	52676.6	8.33	11	50	0		
12	MACAWRSRSRADPLNWGKAAEELSGSHLEAVKRMVEEYRKPVVTMEGATTIAMVAAVAAGSDTRVELDESARGRVKESSDWVMNSMMNGTDSYGVTTGFGATSHRRTKEGGALQRELIRFLNAGAFGTGTDGHVLPAAA<br>TRAAMLVRVNTLLQGYSGIRFEILETIATLLNANVTPCLPLRGTITASGDLVPLSYIAGLVTGRPNSMATAPDGSKVNAAEAFKIAGIQHGFFELQPKEGLAMVNGTAVGSGLASMVLFEANVLSLLAEVLSGVFCEVMNGKPEF<br>TDHLTHKLKHHPGQIEAAAIMEHILEGSSYMMLAKKLGELDPLMKPKQDRYALRTSPQWLGPQIEVIRAATKSIEREINSVNDNPLIDVSRGKAIHGGNFQGTPIGVSMDNTRLAIAAIGKLMFAQFSELVNDFYNNGLPSNLSG<br>GRNPSLDYGFKGAEIAMASYCSELQFLGNPVTNHVQSAEQHNQDVNSLGLISSRKTAEAIDILKLMSSTFLVALCQAIDLRHLEENVKNAVKSCVKTVARKTLSTDNNGHLHNARFCEKDLLLTIDREAVFAYADDPCSANYPL<br>MQKMRAVLVEHALANGEAEAHVETSVFAKLAMFEQELRAVLPKEVEAARSAVENGTAAQQNRIAECRSYPLYRFVRKELGTEYLTGEKTRSPGEEVDKVFVAMNQGKHIDALLECLKEWNGEPLPLC									
	1	Phenylalanine ammonia-lyase	gi|3024363	75889.5	6.10	10	105	100	74	100
	2	Phenylalanine ammonia-lyase [Triticum aestivum]	gi|371928984	55174.2	5.88	4	82	99.997	74	100
	3	Inducible phenylalanine ammonia-lyase [Triticum	gi|9965440	59360.4	5.88	4	82	99.997	74	100
	4	Cellulose synthase [Triticum aestivum], expressed	gi|299109313	111072.7	6.07	9	24	0	8	0
	5	Ribulose-bisphosphate carboxylase (EC 4.1.1.39) small chain precursor (clone 234) - wheat (fragment)	gi|82619	15344.9	8.95	4	22	0		
	6	Ribulosebisphosphate carboxylase [Triticum aestivum]	gi|755804	18119.9	8.95	4	22	0		
	7	G protein alpha subunit [Triticum aestivum]	gi|22336036	45247.1	5.75	6	22	0		
	8	P450 [Triticum aestivum]	gi|62148972	59565.9	6.77	7	21	0		
	9	ferritin [Triticum aestivum]	gi|157674655	28167.2	5.67	5	21	0		
	10	alpha-1 purothionin [Triticum aestivum]	gi|1304262	14531.8	5.13	5	21	0		
13	HIYLSDLMRFMRQEEALKAMDLFEGAQEQNRVSKRSLKNWVVNAFRERKALALTLNDTKTAVNKLHHMANVVVALIVFALWLLILGFATTKTFVFLSSQLLVAAFIFGNTLKTIFEAIIFLFVMHPFDVGDRCEVDGMQVVVE<br>EMNIMTTIFLRYDNLKVYYPNSKLATLPIMNYYRSPDMGDAVDFSVNVATPPEKLALMKERLMQYKEHWYPGSMVVLRDIDDTNRLKISIWCRHTINFQDMGMRFDRRELILQEMMKILRDLDIEYRMLPLDINVRNAPPINS<br>TRMPTTWALNF									
	1	Mechanosensitive ion channel [Triticum aestivum]	gi|86439721	34712.9	8.46	8	30	0		
	2	PPR repeat domain containing protein [Triticum aestivum]	gi|300681579	81867.8	6.21	11	27	0		
	3	PDI-like protein [Triticum aestivum], putative	gi|299469376	40249.9	6.17	7	27	0		
	4	Serine/threonine protein kinase [Triticum aestivum]	gi|14571547	45928.1	8.39	7	26	0		
	5	Annexin [Triticum aestivum]	gi|38606205	35416.1	9.01	7	26	0		
	6	Glycosyltransferase [Triticum aestivum]	gi|56409844	65081	5.95	8	26	0		
	7	Cyclase/dehydrase, putative, expressed [Triticum aestivum]	gi|300681463	25605.9	9.60	5	24	0		
	8	S-locus receptor kinase [Triticum aestivum], putative	gi|62131096	59470.1	7.12	8	24	0		
	9	Oxygen-evolving enhancer protein 2, chloroplastic	gi|131394	27252.8	8.84	5	22	0		
	10	homeobox-like resistance [Triticum aestivum]	gi|166210345	37428.5	5.86	6	22	0		

	MSPQTETKAGVGFKAGVKDYKLTYYTPEYETKDTDILAAFRVSPQPGVPPEEAGAAVAAESSTGTWTTVWTDGLTSLDRYKGRCYHIEPVAGEDSQWICYVAYPLDLFEEGSVTNMFTSIVGNVFGFKALRALRLEDLRIPPTY<br>SKTFQGPPHGIQVERDKLNKYGRPLLGCTIKPKLGLSAKNYGRACYECLRGGLDFTKDDENVNSQPFMRWRDRFVFCAEAIYKSQAETGEIKGHYLNATAGTCEEMIKRAVFARELGVPIVMHDYLTGGFTANTTLAHYCRD<br>NGLLLHIHRAMHAVIDRQKNHGMHFRVLAKALRMSGGDHIHSGTVVGKLEGEREMTLGFVDLLRDDFIEKDRARGIFFTQDWVSMPGVIPVASGGIHVWHMPALTEIFGDDSVLQFGGGTLGHPWGNAPGAAANRVALEAC<br>VQARNEGRDLAREGNEIIRAACKWSPELAAACEVWKAIKFEFEPVDTIDK									
14	1	Ribulose-1,5-bisphosphate carboxylase/oxygenase large subunit [Triticum aestivum]	gi|14017580	52817.4	6.22	14	149	100	82	100
	2	Ribulose-1,5-bisphosphate carboxylase/oxygenase large subunit [Triticum aestivum]	gi|32966580	52757.3	6.22	14	148	100	82	100
	3	Ribulose-1,5-bisphosphate carboxylase/oxygenase large subunit [Triticum aestivum]	gi|339742637	21900	7.57	7	129	100	82	100
	4	Ribulose-1,5-bisphosphate carboxylase/oxygenase large subunit, partial [Triticum aestivum]	gi|313664567	22368.2	5.78	6	119	100	82	100
	5	Ribulose 1,5-bisphosphate carboxylase/oxygenase large subunit [Triticum aestivum]	gi|12344	46972.5	6.60	10	38	25.245		
	6	Starch synthase IIIb precursor [Triticum aestivum]	gi|189096677	143134.1	5.13	13	27	0		
	7	Multiple inositol polyphosphate phosphatase PhyIIc [Triticum aestivum]	gi|116294385	58093.8	7.55	8	25	0		
	8	Multiple inositol polyphosphate phosphatase PhyIIb [Triticum aestivum]	gi|116294383	58056.7	7.55	8	24	0		
	9	ADP-glucose pyrophosphorylase small subunit, plastid [Triticum aestivum]	gi|224021585	56253.8	6.40	7	23	0		
	10	WRKY39 transcription factor [Triticum aestivum]	gi|189172061	26186.2	8.98	5	23	0		
	MSPQTETKAGVGFKAGVKDYKLTYYTPEYETKDTDILAAFRVSPQPGVPPEEAGAAVAAESSTGTWTTVWTDGLTSLDRYKGRCYHIEPVAGEDSQWICYVAYPLDLFEEGSVTNMFTSIVGNVFGFKALRALRLEDLRIPPTY<br>SKTFQGPPHGIQVERDKLNKYGRPLLGCTIKPKLGLSAKNYGRACYECLRGGLDFTKDDENVNSQPFMRWRDRFVFCAEAIYKSQAETGEIKGHYLNATAGTCEEMIKRAVFARELGVPIVMHDYLTGGFTANTTLAHYCRD<br>NGLLLHIHRAMHAVIDRQKNHGMHFRVLAKALRMSGGDHIHSGTVVGKLEGEREMTLGFVDLLRDDFIEKDRARGIFFTQDWVSMPGVIPVASGGIHVWHMPALTEIFGDDSVLQFGGGTLGHPWGNAPGAAANRVALEAC<br>VQARNEGRDLAREGNEIIRAACKWSPELAAACEVWKAIKFEFEPVDTIDK									
15	1	Ribulose-1,5-bisphosphate carboxylase/oxygenase large subunit [Triticum aestivum]	gi|14017580	52817.4	6.22	25	806	100	611	100
	2	Ribulose-1,5-bisphosphate carboxylase/oxygenase large subunit [Triticum aestivum]	gi|32966580	52757.3	6.22	24	793	100	611	100
	3	Ribulose-1,5-bisphosphate carboxylase/oxygenase large subunit [Triticum aestivum]	gi|12344	46972.5	6.60	20	574	100	434	100
	4	Ribulose-1,5-bisphosphate carboxylase/oxygenase large subunit [Triticum aestivum]	gi|339742637	21900	7.57	11	411	100	321	100
	5	Ribulose-1,5-bisphosphate carboxylase/oxygenase large subunit, partial [Triticum aestivum]	gi|313664567	22368.2	5.78	10	399	100	321	100
	6	Ribulose-1,5-bisphosphate carboxylase/oxygenase large subunit (30 AA) [Triticum aestivum]	gi|12366	3423.7	4.66	2	130	100	113	100
	7	P450 [Triticum aestivum]	gi|48762803	59137.5	6.41	11	45	85.424		
	8	P450 [Triticum aestivum]	gi|62148972	59565.9	6.77	9	32	0		
	9	P450 [Triticum aestivum]	gi|48526681	59208.6	6.61	9	32	0		
	10	P450 [Triticum aestivum]	gi|48526683	59201.6	6.30	9	32	0		
	MAICKVWISLLLALAVVLSAPAARAEEAAAAAEEAAAPEAVLTLHADNFDDAIAKHPFILVEFYAPWCGHCKSLAPEYEKAAQLLSKHDPAIVLAKVDANDEKNKPLAGKYEVQGFPTLKIFRNGGKNIQEYKGPREAEGIVE<br>YLKKQVGPASKEIKAPEDATYLEDGKIHIVGVFTEFSGTEFTNFLELAEKLRSDYDFGHTVHANHLPRGDAAVERPLVRLFKPFDELVVDSKDFDVSALEKFIDASSTPKVVTFDKNPDNHPYLLKFFQTNAPKAMLFLNFSTGP<br>FESFKSAYYGAVEEFSGKDVKFLIGDIEASQGAFQYFGLKEDQAPLILIQDSDSKKFLKEQVEAGQIVAWLKDYFDGKLTPFRKSEPIPEANNEPVKVVVADNVHDVVFKSGKNVLIEFYAPWCGHCKKLAPILDEAAATLQSEE<br>DVVIAKMDATANDVPSEFDVQGYPTLYFVTPSGKKVSYEGGRTADEIVDYIKKNKETAGQAAAADTEKAAEPAATEPLKDEL									
16	1	Protein disulfide isomerase [Triticum aestivum]	gi|222446340	56635.9	4.96	30	803	100	532	100
	2	Protein disulfide-isomerase	gi|1709620	56497.9	4.99	29	787	100	532	100
	3	Protein disulfide isomerase 1 proprotein [Triticum aestivum]	gi|13925723	56544	4.99	28	773	100	532	100
	4	Protein disulfide isomerase [Triticum aestivum]	gi|222446344	56635.9	4.93	30	723	100	454	100
	5	Protein disulfide isomerase [Triticum aestivum]	gi|222446342	56377.8	5.03	29	709	100	454	100
	6	Protein disulfide isomerase [Triticum aestivum]	gi|335906219	56333.8	5.06	28	695	100	454	100
	7	Protein disulfide isomerase [Triticum aestivum]	gi|335906215	56611.9	4.93	25	655	100	454	100
	8	Protein disulfide isomerase [Triticum aestivum]	gi|335906217	56364.7	4.96	24	643	100	454	100
	9	Protein disulfide isomerase precursor [Triticum aestivum]	gi|67508765	56559.9	4.99	26	550	100	340	100
	10	Protein disulfide isomerase [Triticum aestivum]	gi|47118054	16692.4	5.16	9	540	100	464	100
	MAMVDEPLYPIAVLIDELKNEDIQLRLNSIRRLSTIARALGEERTRKELIPFLSENNDDEDEVLLAMAEELGVFIPYVGGVEHAHVLLPPLETLSTVEETCVRDKAVESLCRIGAQMKENDIVDYFIAVVKRLAAGEWFTARVSSC<br>GLFHIAYPSATDPLKTELRTIYGQLCQDDMPMVRRAAASNLGKFAATVEQSHLKTEIMSIFDDLTQDDQDSVRLLAVEGCAALGKLLEPQDCVAHILPVIVNFSQDKSWRVRYMVANQLYELCEAVGPEPTRADLVPAYVRLLR<br>DNEAEVRIAAAGKVTKFCRILSPQLAIQHILPCVKELSSDSSQHVRSALASVIMGMAPVLGKDATIEQLLPIFLSLLKDEFPDVRLNIISKLDQVNQVIGIDLLSQSLLPAIVELAEDRHWRVRLAIIEYIPLLASQLGVGFFDDKLG<br>ALCMQWLEDKVFSIREAAANNLKRLAEEFGPEWAMQHIIPQVLEKINNPHYL									
	YRMTILQAISLLAPVMGAEITCQKLLPVVINSSKDRVPNIKFNVAKVLQSLVPILDQSVAEKTVKPCLVELSEDPDVDVRYYANQALQACDQIMMSS									
	1	Protein phosphatase 2A structural subunit [Triticum aestivum]	gi|328775741	65460.1	4.95	18	514	100	403	100
	2	Protein disulfide isomerase [Triticum aestivum]	gi|222446342	56377.8	5.03	20	233	100	100	100

17	3	Protein disulfide isomerase [Triticum aestivum]	gi|335906219	56333.8	5.06	19	222	100	100	100
	4	Protein disulfide isomerase [Triticum aestivum]	gi|222446344	56635.9	4.93	19	222	100	100	100
	5	Protein disulfide isomerase [Triticum aestivum]	gi|335906217	56364.7	4.96	18	212	100	100	100
	6	Protein disulfide isomerase [Triticum aestivum]	gi|222446340	56635.9	4.96	16	192	100	100	100
	7	Protein disulfide-isomerase, precursor	gi|1709620	56497.9	4.99	16	191	100	100	100
	8	Protein disulfide isomerase [Triticum aestivum]	gi|335906215	56611.9	4.93	15	182	100	100	100
	9	Protein disulfide isomerase 1 proprotein [Triticum	gi|13925723	56544	4.99	15	182	100	100	100
	10	Protein disulfide isomerase [Triticum aestivum]	gi|48093451	10215.2	5.43	7	166	100	100	100
18	RASPPSESRAPLRAPQRSATRQHQARQGPRRMCNGGRGPPYWTAGVTSAPARQTPLFSGRPSGGLSDPNEVAAVILGGGTGTQLFPLTSTRATPAVPIGGCYRLIDIPMSNCFNSGINKIFVMTQFNSASLNRHIHRTYLGGGINFT<br>DGSVEVLAATQMPGEAAGWFRGTADAWRKIIWVLEDYYKNKSIEHILILSGDQLYRMDYMELVQKHVDDNADITLSCAPVGESRASEYGLVKFDSSGRVVQFSEQPKGDDLEAMKVDTSFLNFAIDDPAKYPYIASMGVYVF<br>KRDVLLNLLKSRYAELHDFGSEILPRALHDHNVQAYVFTDYWEDIGTIRSFFDANRALCEQPPKFEFYDPKTPFFTSPRYLPPTKSDKCRIKEAIILHGCFLRECKIEHTAFSRLNSGSELKNAMMMGADSYETEDEMSRLMSEG<br>KVPIGVGENTKISNCIIDMNARIGRDVVISNKEGVQEADRPEEGYYIRSGIVVIQKNATIKDGTVV									
	1	Adenylosuccinate synthetase, chloroplastic (Fragment) [Triticum aestivum]	PURA_WHEAT	50886.1	5.93	8	26	12.367		
	2	Glucose-1-phosphate adenylyltransferase large subunit,chloroplastic/amyloplastic (Fragment) [Triticum aestivum]	GLGL3_WHEAT	55524.7	6.61	6	26	8.237	12	96.153
	3	Ribulose bisphosphate carboxylase small chain clone 512 (Fragment) [Triticum aestivum]	RBS3_WHEAT	13046.5	5.84	4	22	0		
	4	Alpha-2-purothionin OS=Triticum aestivum GN=THI1.2	THN2_WHEAT	14547.8	5.13	5	19	0		
	5	Phosphoribulokinase, chloroplastic [Triticum aestivum]	KPPR_WHEAT	45112.9	5.72	6	18	0		
	6	Flavone O-methyltransferase 1 OS=Triticum aestivum	FOMT1_WHEAT	38834.6	5.75	5	16	0		
	7	30S ribosomal protein S11, chloroplastic [Triticum aestivum]	RR11_WHEAT	15556.6	12.01	4	16	0		
	8	Gibberellin 20 oxidase 1-A OS=Triticum aestivum	GAO1A_WHEAT	40679.3	6.27	4	15	0	4	78.614
	9	Dihydrodipicolinate synthase 2, chloroplastic [Triticum aestivum]	DAPA2_WHEAT	40849.9	6.80	5	15	0		
	10	Protochlorophyllide reductase A, chloroplastic [Triticum aestivum]	PORA_WHEAT	41130.1	9.42	5	15	0		
19	MSSAVASAASFLALASASPGRSRRRTRVSASPPHTGAGRLHWPPSPPQRTARDGAVAARAAGKKDAGIDDAAPARQPRALRGGAATKVAERRDPVKTLDRDAAEGGAPSPPAPRQEDARLPSMNGMPVNGENKSTGGGATK<br>DSGLPAPARAPQPSSQNRVPVNGENKANVASPPTSIAEVAAPDPAATISISDKAPESVVPAEKAPPSSGSNFVPSASAPGSDTVSDVELELKKGAVIVKEAPNPKALSPPAAPAVQQDLWDFKKYIGFEEPVEAKDDGRAVADDAG<br>SFEHHQNHDSGPLAGENVMNVVVVAAECSPWCKTGGLGDVAGALPKALAKRGHRVMVVVPRYGDYEEAYDVGVRKYYKAAGQDMEVNYFHAYIDGVDFVFIDAPLFRHRQEDIYGGSRQEIMKRMILFCKAAVEVPWH<br>VPCGGVPYGDGNLVFIANDWHTALLPVYLKAYYRDHGLMQYTRSIMVIHNIAHQGRGPVDEFPFTELPEHYLEHFRLYDPVGGEHANYFAAGLKMADQVVVVSPGYLWELKTVEGGWGLHDIIRQNDWKTRGIVNGIDNM<br>EWNPEVDVHLKSDGYTNFSLGTLDSGKRQCKEALQRELGLQVRGDVPLLGFIGRLDGQKGVEIIADAMPWIVSQDVQLVMLGTGRHDLEGMLRHFEREHHDKVRGWVGFSVRLAHRITAGADALLMPSRFEPCGLNQLYA<br>MAYGTVPVVHAVGGLRDTVPPFDPFNHSGLGWTFDRAEAQKLIEALGHCLRTYRDYKESWRGLQERGMSQDFSWEHAAKLYEDVLVKAKYQW									
	1	Starch synthase IIa-3 [Triticum aestivum]	gi|8953573	86740.6	6.27	7	37	0	25	93.636
	2	Starch synthase IIa-1 [Triticum aestivum]	gi|7529653	86891.4	6.05	6	36	0	25	93.636
	3	Starch synthase IIA [Triticum aestivum]	gi|5825480	87179.8	6.20	6	35	0	25	93.636
	4	MYB-related protein [Triticum aestivum]	gi|359950756	60684.5	9.43	11	34	0		
	5	Starch synthase [Triticum aestivum]	gi|1620660	55636.9	6.15	4	34	0	25	93.636
	6	Thioredoxin H [Triticum aestivum]	gi|27461140	12685.4	5.29	5	28	0		
	7	MYB-related protein [Triticum aestivum]	gi|359952782	48051.4	8.93	6	28	0	11	0
	8	Dehydroascorbate reductase [Triticum aestivum]	gi|259017810	23343.1	5.88	5	27	0	4	0
	9	CHY-type zinc finger protein [Triticum aestivum]	gi|282767742	32057.4	6.70	8	24	0		
	10	Starch branching enzyme II [Triticum aestivum]	gi|145666648	15223.9	4.85	2	24	0	16	48.861
20	MDTITVVENVFGEVERKVQKSYWEEHSKDLTVESMMLDSRAKDLDKEERPEVLAILPSYAGKTVLELGAGIGRFTGELAKEAGHVIALDFIDSVIKKNEEINGDIYKNITFMCADVTSPELKIEDNSVDIVFSNWLLMYLNDEE<br>VEKLIGRIVKWLKPGGHIFIRESCFHQSGDSKRKVNPTHYREPRFYTKVFKECHSYDQEGNSFELSLVTSKCIGAYVKSKKNQNQICWLWEKVKCTEDKGFQRFLDNVQYKSTGILRYERVFGEGYVSTGGFETTKEFVDKLDL<br>KAGQKVLDVGCGIGGGDFYMAETYDVHVLGIDLSINMVSFAIERAIGRSCSVEFEVADCTTKEYAENTFDVIYSRDTILHIQDKPALFRNFFKWLKPGGKVLISDYCRSPGTPSEEFAAYIKQRGYDLHDVKTYGKMLEDAGFH<br>DVVAEDRTDQFLRVLERELGETEKNKEAFLADFTQEDYDDIVNGWSAKLKRSSAGEQKWGLFIATK									
	1	Beta-amylase/1,4-alpha-D-glucan maltohydrolase	gi|3334120	56575.1	5.24	18	696	100	575	100
	2	phosphoethanolamine methyltransferase [Triticum aestivum]	gi|17887465	56822.4	5.21	12	128	100	72	100
	3	Vacuolar ATPase subunit B1 [Triticum aestivum]	gi|125744990	53958.6	5.12	10	42	67.368		
	4	Peroxiredoxin [Triticum aestivum]	gi|190684059	23962.2	6.31	6	38	26.947	7	0
	5	1-Cys peroxiredoxin PER1/Rehydrin homolog/Thioredoxin p	gi|75324900	23950.1	6.08	6	38	21.722	7	0
	6	Kinesin heavy chain isoform [Triticum aestivum], putative	gi|226434268	31496.7	5.31	7	30	0		
	7	Ent-copalyl diphosphate synthase [Triticum aestivum]	gi|226528027	90143	5.88	10	28	0		

	8	Heat shock protein HSP26 [Triticum aestivum]	gi|4028567	26482.4	9.36	6	26	0		
	9	Ribulose-1,5-bisphosphate carboxylase/oxygenase small subunit [Triticum aestivum]	gi|11990901	19448.8	8.81	5	24	0		
	10	Ribulose bisphosphate carboxylase small chain PW9, chloroplastic	gi|132099	19441.7	8.52	5	24	0		
21	MRECISIHIGQAGIQVGNACWELYCLEHGIQPDGQMPGDKTVGGGDDAFNTFFSETGAGKHVPRAVFVDLEPTVIDEVRTGAYRQLFHPEQLISGKEDAANNFARGHYTIGKEIVDLCLDRIRKLSDNCTGLQGFLVFNAVGGG<br>TGSGLGSLLLERLSVDYGKKSKLGFTVYPSPQVSTSVVEPYNSVLSTHSLLEHTDVSILLDNEAIYDICRRSLDIERPTYTNLNRLVSQVISSLTASLRFDGALNVDVNEFQTNLVPYPRIHFMLSSYAPVISAEKAYHEQLSVAEIT<br>NSAFEPSSMMAKCDPRHGKYMACCLMYRGDVVPKDVNAAVATIKTKRTIQFVDWCPTGFKCGINYQPPGVVPGGDLAKVQRAVCMISNSTSVVEVFSRIDHKFDLMYAKRAFVHWYVGEGMEEGEFSEAREDLAALEKDY<br>EEVGAEFDEGEDGDEGDEY									
	1	Alpha tubulin-2A [Triticum aestivum]	gi|90289596	49683.3	4.89	15	304	100	212	100
	2	Tubulin alpha-3 chain	gi|8928432	49697.4	4.89	15	304	100	212	100
	3	Alpha tubulin-4A [Triticum aestivum]	gi|90289610	49755.4	4.81	11	267	100	212	100
	4	Alpha tubulin-1D [Triticum aestivum]	gi|90289618	49579.5	4.95	10	171	100	124	100
	5	Alpha tubulin-1B [Triticum aestivum]	gi|90289622	49619.6	4.99	8	155	100	124	100
	6	ATP synthase beta subunit [Triticum aestivum]	gi|525291	59211.9	5.56	6	93	100	74	100
	7	Annexin [Triticum aestivum]	gi|38606205	35416.1	9.01	8	46	86.397	11	0
	8	Ribulose-bisphosphate carboxylase (EC 4.1.1.39) small chain precursor (clone 234) - wheat (fragment)	gi|82619	15344.9	8.95	6	41	57.962		
	9	Ribulose bisphosphate carboxylase small chain clone 512	gi|132107	13046.5	5.84	5	33	0		
	10	Ribulose-1,5-bisphosphate carboxylase/oxygenase small subunit [Triticum aestivum]	gi|11990893	19500.8	9.06	6	32	0		
22	MRECISIHIGQAGIQVGNACWELYCLEHGIQPDGQMPGDKTVGGGDDAFNTFFSETGAGKHVPRAVFVDLEPTVIDEVRTGAYRQLFHPEQLISGKEDAANNFARGHYTIGKEIVDLCLDRIRKLSDNCTGLQGFLVFNAVGGG<br>TGSGLGSLLLERLSVDYGKKSKLGFTVYPSPQVSTSVVEPYNSVLSTHSLLEHTDVSILLDNEAIYDICRRSLDIERPTYTNLNRLVSQVISSLTASLRFDGALNVDVNEFQTNLVPYPRIHFMLSSYAPVISAEKAYHEQLSVAEIT<br>NSAFEPSSMMAKCDPRHGKYMACCLMYRGDVVPKDVNAAVATIKTKRTIQFVDWCPTGFKCGINYQPPGVVPGGDLAKVQRAVCMISNSTSVVEVFSRIDHKFDLMYAKRAFVHWYVGEGMEEGEFSEAREDLAALEKDY<br>EEVGAEFDEGEDGDEGDEY									
	1	Alpha tubulin-2A [Triticum aestivum]	gi|90289596	49683.3	4.89	8	65	99.84	28	97.22
	2	Tubulin alpha-3 chain	gi|8928432	49697.4	4.89	7	57	99.101	28	97.22
	3	Alpha tubulin-4A [Triticum aestivum]	gi|90289610	49755.4	4.81	6	51	95.698	28	97.22
	4	Ribulose-1,5-bisphosphate carboxylase/oxygenase small subunit [Triticum aestivum]	gi|11990893	19500.8	9.06	6	39	39.237		
	5	Ribulose-1,5-bisphosphate carboxylase/oxygenase small subunit [Triticum aestivum]	gi|11990897	19447.8	8.80	6	38	23.504		
	6	ADP-glucose pyrophosphorylase small subunit, plastid [Triticum aestivum]	gi|224021585	56253.8	6.40	8	34	0		
	7	Bowman-Birk trypsin inhibitor-like protein [Triticum aestivum]	gi|162319710	9856.6	8.67	5	34	0		
	8	D1 protease precursor [Triticum aestivum]	gi|19774133	42003.6	5.72	7	32	0		
	9	5-methylcytosine DNA glycosylase [Triticum aestivum]	gi|333471381	218295.3	6.06	15	30	0		
	10	Ribulose-1,5-bisphosphate carboxylase/oxygenase small subunit [Triticum aestivum]	gi|11990901	19448.8	8.81	5	30	0		
23	MAFRRILSAAVRRRSAAAAAAAGNAREASTAVAAGPGVIGPDAPAVRAPLMPYDRISEAVNARLRRLEHPDPRFLRYASPVPAHVDHTAILAAPETRVTTLPNGLRVATESSLAARTATVGVWIDAGSRYEKDSQAGVAHFVEH<br>MLFKGTGMRTAGQLEQEIEDIGGHLNAYTSREQTTYYAKVLDKDVPRALEVLADILQNSNLAEERIERERDVILREMEEVEGQSEEVIFDHLHATAFQYTSLGRPILGSADNVRSITQEDLKAYIKTHYTAPRMVITAAGAVKHE<br>DIVEQATKLFDKLSTDPTTTSMLVDKEPASFTGSEVRIIDDDMPLAQFAVAFNGASWADPDSIALMVMQTMLGSWNKSAGGGKHMGSELVQRVAINDIAESIMAFNTNYKDTGLFGVYAVAKADCLDDLAFAIMQEMSKLSYR<br>VTEEDVIRARNQLKSSIQLHLDGSTAVVEDIGRQLLIYGRRIPIPELFARIDAVDPSTIRHVANRFIFDQDVAIAAMGPIQSLPDYNWFRRRTYMLRY									
	1	Predicted protein [Hordeum vulgare subsp. vulgare]	gi|326513540	58472.9	6.05	17	266	100	172	100
	2	Mitochondrial-processing peptidase subunit beta-like [Brachypodium distachyon], predicted	gi|357113428	58384.8	5.63	13	219	100	160	100
	3	Hypothetical protein OsJ_09894 [Oryza sativa Japonica Group]	gi|222624442	53597.1	5.18	10	200	100	160	100
	4	Hypothetical protein OsI_10505 [Oryza sativa Indica Group]	gi|125542883	58719.9	5.50	10	196	100	160	100
	5	LOC100283246 [Zea mays]	gi|226508302	58406.6	5.87	10	139	100	103	100
	6	Uncharacterized protein LOC100191520 [Zea mays]	gi|212275227	58342.7	5.96	9	130	100	103	100
	7	Hypothetical protein SORBIDRAFT_01g043060 [Sorghum bicolor]	gi|242041787	58336.7	6.05	8	126	100	103	100
	8	Floricaula protein [Misopates orontium]	gi|82734197	44863.4	6.06	13	66	72.186		
	9	Phosphoenolpyruvate carboxylase [Dendrobium loddigesii]	gi|7768113	41211.6	9.32	12	65	66.56		
	10	hypothetical protein [Zea mays]	gi|195615876	10109.2	6.26	7	64	62.48		
	MFIESFKVESPNVRYGAGEIESEYRYDTTELVHESHDGASKWVVRPKSVNYHFKTNTTVPKLGVMLVGWGGNNGSTLMAGVIANREGISWATKDKVQQANYFGSLTQASTIRVGSYNGEEIYAPFKSLLPMVNPDDLVFGGW<br>DISSMNLADAMTRTKVLDIDLQKQLRPYMESIVPLPGIYDPDFIAANQGSRANNVIKGTKKEQMEQIIKDIREFKEKNKVDKVVVLWTANTERYSNVSVGLNDTMENLLASVDKNEAEISPSTLYAIACVMEGVPFINGSPQNTF<br>VPGLIDLAIKNNCLIGGDDFKSGQTKMKSVLVDFLVGAGIKPTSIVSYNHLGNNDGMNLSAPQTFRSKEISKSNVVDDMVSSSAILYKPGEHPDRVVVIKYVPYVGDSKRAMDEYTSEIFMGGKSTIVLHNTCEDSLLAAPIILD<br>LVLLAELSTRIQLKAEGEDKFHSFHPVATILSYLTKAPLVPPGTPVVNALAKQRAMLENIMRACVGLAPENNMILEYK									
	1	Myo-inositol-1-phosphate synthase [Triticum aestivum]	gi|351767989	56086.6	5.64	20	822	100	663	100

24	2	Inositol-3-phosphate synthase/Myo-inositol-1-phosphate synthase	gi|14548098	56258.6	5.38	8	30	0		
	3	High molecular weight glutenin subunit Dx5 [Triticum	gi|343174543	13214.8	5.12	4	28	0		
	4	MYB-related protein [Triticum aestivum]	gi|359950756	60684.5	9.43	8	27	0		
	5	Pm3 [Triticum aestivum]	gi|296280026	159148.2	6.30	16	24	0		
	6	Zinc finger protein [Triticum aestivum]	gi|255642812	47342.4	8.82	6	23	0		
	7	Glycosyltransferase, HGA-like [Triticum aestivum], putative,expressed	gi|300681530	68832	6.38	8	22	0		
	8	Ribulose-bisphosphate carboxylase (EC 4.1.1.39) small chain precursor (clone 234) - wheat (fragment	gi|82619	15344.9	8.95	4	22	0		
	9	Ribulosebisphosphate carboxylase [Triticum aestivum]	gi|755804	18119.9	8.95	4	22	0		
	10	HMW glutenin subunit [Triticum aestivum]	gi|45533895	20813.4	5.15	4	22	0		
25		MAGPSSPSSTSRPRRREGVENLFERMDRMVAHGPNFCDITWGAGGSTADVTLDIANRMQNMVCVETMMHLTCTNMPVEKIDNALDTIKSNGIQNVLALRGDPPHGQDKFVQVAGGFSCALDLVEHIKAKYGDYFGITVAGY PEAHPEVILGEEGATEEAYRKDLAYLKRKVDAGADVIVTQLFYDTDIFLKFVNDCRQIGITCPIVPGIMPINNYKGFVRMTGFCKTKIPAEITAALDPIKDNEEAVKAYGIHLGTEMCKKILASGIKTLHLYTLNMEKTALAILMNL GLIEESKLSRTLPWRPPTNVFRVKEDVRPIFWANRPKSYISRTTGWDQYPHGRWGDSRNPSYGALNDHQFTRPRGRGKKLQEEWAVPLKSVQDINERFVNFCEGKLKSSPWSELDGLQPETTIIDDQLVKINSKGFLTINSQPAVN AEKSESPSVGWGGPGGYVYQKAYVEFFCAKEKLGQLIEKSKAFPSLTYIAVNKEGESISNIPANAVNAVTWGVFPGKEIIQPTVVDSASFMVWKDEAFEIWSRGWACLFPEGDSSRELLEQIQKSYYLVSLVDNDYISGDLFAAF KEI								
	1	5,10-methylene-tetrahydrofolate reductase [Triticum monococcum]	gi|115589742	64833.7	5.86	30	382	100	150	100
	2	Os03g0815200 [Oryza sativa Japonica Group]	gi|115456165	66388.3	5.38	8	187	100	169	100
	3	Methylenetetrahydrofolate reductase, 3-partial [Oryza sativa Japonica Group]	gi|37718877	41789	6.10	6	187	100	169	100
	4	Hypothetical protein SORBIDRAFT_01g003700 [Sorghum bicolor]	gi|242032523	66298.5	5.73	11	151	100	115	100
	5	Methylenetetrahydrofolate reductase 1 [Zea mays]	gi|162459521	66386.4	5.62	10	145	100	115	100
	6	Methylenetetrahydrofolate reductase-like [Brachypodium distachyon], predicted	gi|357124307	66224.2	5.31	14	105	99.997	39	57.793
	7	Methylenetetrahydrofolate reductase 2-like [Glycine max]	gi|356517828	66893.6	5.60	10	64	59.797	36	13.765
	8	Methylenetetrahydrofolate reductase [Populus trichocarpa]	gi|224117802	67453.1	6.01	8	60	0	39	57.793
	9	Methylenetetrahydrofolate reductase 2-like [Glycine max], predicted	gi|356509485	66953.7	5.76	9	57	0	36	13.765
	10	conserved hypothetical protein [Ricinus communis]	gi|255606550	5468.7	8.94	6	54	0		
26		MEFSPRAAELTTLLESRMTNFYTNFQVDEIGRVVSVGDGIARVYGLNEIQAGEMVEFASGVKGIALNLENENVGIVVFGSDTAIKEGDLVKRTGSIVDVPAGKAMLGRVVDALGVPIDGKGALSDHERRRVEVKAPGIIERKSV HEPMQTGLKAVDSLVPIGRGQRELIIGDRQTGKTAIAIDTILNQKQMNSRGTNESETLYCVYVAIGQKRSTVAQLVQILSEANALEYSILVAATASDPAPLQFLAPYSGCAMGEYFRDNGMHALIIYDDLSKQAVAYRQMSLLLRR PPGREAFPGDVFYLHSRLLERAAKRSDQTGAGSSTALPVIETQAGDVSAYIPTNVISITDGQICLETELFYRGIRPAINVGLSVSRVGSAAQLKAMKQVCGSSKLELAQYREVAAFAQFGSDLDAATQALLNRGARLTEVPKQPQ YEPLPIEKQIVVIYAAVNGFCDRMPLDRISQYEKAILSTINPELQKSFLEKGGLTNERKMEPDASLKESTLPYL								
	1	ATP1 [Triticum aestivum]	gi|81176509	55271.7	5.70	25	752	100	550	100
	2	ATP synthase subunit alpha, mitochondrial	gi|114419	55229.6	5.70	24	487	100	299	100
	3	Cold acclimation protein WCOR615 [Triticum aestivum]	gi|1657857	17773.5	4.92	7	37	3.697		
	4	Glycosyltransferase, HGA-like [Triticum aestivum], putative,expressed	gi|300681530	68832	6.38	9	28	0		
	5	Expansin EXPB4 [Triticum aestivum]	gi|44894802	29008.4	8.03	6	27	0		
	6	Conserved hypothetical protein, expressed [Triticum aestivum]	gi|300681533	65462.9	9.33	8	25	0		
	7	Type 1 non specific lipid transfer protein precursor [Triticum aestivum]	gi|84617223	11139.6	9.20	4	25	0		
	8	Cyclophilin [Triticum aestivum]	gi|82547214	13583.8	9.19	4	25	0		
	9	Non-specific lipid transfer protein 1 precursor [Triticum	gi|94315719	11110.6	9.20	4	24	0		
	10	Phenylalanine ammonia-lyase	gi|3024363	75889.5	6.10	9	24	0	6	0
27		MVFSVTKKETKPYEGQKPGTSGLRKKVTVFQQPHYLANFVQSTFNALPADQVKGATIVVSGDGRYFSKDAVQIIAKMAAANGVRRVWVGQDSLLSTPAVSAIIRERISADGAKATGAFILTASHNPGGPTEDFGIKYNMGNGGP APESVTDKIFSNTKTVTEYLIAEDLPNVDISVIGVTSFTGPEGPFDVDVFDSATDYIKLMKTIFDFESIKKLLASPKFSFCFDGLHGVAGAYAKRMFVDELGASESSLLNCVPKEDFGGGHPDPNLTYAKELVDRMGLGKTSNVEP PEFGAAADGDADRNMVLGKRFFVTPSDSVAIIAANAVQSIPYFASGLKGVARSMPTSAALDVVAKNLNLKFFEVPTGWKFFGNLMDAGMCSVCGEESFGTGSDHIREKDGIWAVLAWLSILAYKNKDNLGGDKLVTVEDIVL QHWATYGRHYYTRYDYENVDAEAAKELMANLVKMQSSLSDVNKSIKEIQPTVADVVSADEFEYKDPVDGSVSKHQGIRYLFGDGSRLVFRLSGTGSVGATIRIYIEQYEKDSSKTGRASSDALSPLVDVALKFSKIKEYTGRSA PTVIT								
	1	Phosphoglucomutase [Triticum aestivum]	gi|18076790	62749.8	5.66	19	762	100	641	100
	2	NADP-dependent malic enzyme [Triticum aestivum]	gi|158701881	63049.4	5.56	14	65	99.854		
	3	Unnamed protein product [Triticum aestivum]	gi|300681516	13915.9	5.92	5	32	0		
	4	Glycosyltransferase, HGA-like [Triticum aestivum], putative,expressed	gi|300681530	68832	6.38	9	28	0		
	5	Ribulose-1,5-bisphosphate carboxylase/oxygenase small subunit [Aegilops longissima]	gi|4038673	18533.4	8.83	5	26	0		
	6	Ferritin 2C [Triticum aestivum]	gi|210061155	22620.2	4.99	5	26	0		

	7	Mitogen-activated protein kinase MAPK1d [Triticum aestivum]	gi|84795224	42746.3	8.82	7	25	0		
	8	Ribulose-1,5-bisphosphate carboxylase/oxygenase small subunit [Triticum aestivum]	gi|11990897	19447.8	8.80	5	24	0		
	9	Ribulose bisphosphate carboxylase small chain clone 512	gi|132107	13046.5	5.84	4	24	0		
	10	pTACR7 [Triticum aestivum]	gi|12656895	11070.4	10.99	5	23	0		
28	MVFSVTKKETKPYEGQKPGTSGLRKKVTVFQQPHYLANFVQSTFNALPADQVKGATIVVSGDGRYFSKDAVQIIAKMAAANGVRRVWVGQDSLLSTPAVSAIIRERISADGAKATGAFILTASHNPGGPTEDFGIKYNMGNGGPAPESVTDKIFSNTKTVTEYLIAEDLPNVDISVIGVTSFTGPEGPFDVDVFDSATDYIKLMKTIFDFESIKKLLASPKFSFCFDGLHGVAGAYAKRMFVDELGASESSLLNCVPKEDFGGGHPDPNLTYAKELVDRMGLGKTSNVEPPEFGAAADGDADRNMVLGKRFFVTPSDSVAIIAANAVQSIPYFASGLKGVARSMPTSAALDVVAKNLNLKFFEVPTGWKFFGNLMDAGMCSVCGEESFGTGSDHIREKDGIWAVLAWLSILAYKNKDNLGGDKLVTVEDIVLQHWATYGRHYYTRYDYENVDAEAAKELMANLVKMQSSLSDVNKSIKEIQPTVADVVSADEFEYKDPVDGSVSKHQGIRYLFGDGSRLVFRLSGTGSVGATIRIYIEQYEKDSSKTGRASSDALSPLVDVALKFSKIKEYTGRSAPTVIT									
	1	Phosphoglucomutase [Triticum aestivum]	gi|18076790	62749.8	5.66	21	609	100	465	100
	2	NADP-dependent malic enzyme [Triticum aestivum]	gi|158701881	63049.4	5.56	13	110	100	50	99.989
	3	Ribulose bisphosphate carboxylase small chain clone 512	gi|132107	13046.5	5.84	5	33	0		
	4	Ribulose-1,5-bisphosphate carboxylase/oxygenase small subunit [Triticum aestivum]	gi|11990897	19447.8	8.80	6	32	0		
	5	Ribulosebisphosphate carboxylase [Triticum aestivum]	gi|755804	18119.9	8.95	5	30	0		
	6	Ribulose-bisphosphate carboxylase (EC 4.1.1.39) small chain precursor (clone 234) - wheat (fragment	gi|82619	15344.9	8.95	5	29	0		
	7	Protein serine/threonine kinase [Triticum aestivum]	gi|310723069	51387.9	8.09	7	24	0		
	8	Ribulose-1,5-bisphosphate carboxylase/oxygenase small subunit [Triticum aestivum]	gi|11990893	19500.8	9.06	5	24	0		
	9	Unnamed protein product [Triticum aestivum]	gi|300681516	13915.9	5.92	4	24	0		
	10	Glycosyltransferase, HGA-like [Triticum aestivum], putative,expressed	gi|300681530	68832	6.38	8	23	0		
29	MVFSVTKKETKPYEGQKPGTSGLRKKVTVFQQPHYLANFVQSTFNALPADQVKGATIVVSGDGRYFSKDAVQIIAKMAAANGVRRVWVGQDSLLSTPAVSAIIRERISADGAKATGAFILTASHNPGGPTEDFGIKYNMGNGGPAPESVTDKIFSNTKTVTEYLIAEDLPNVDISVIGVTSFTGPEGPFDVDVFDSATDYIKLMKTIFDFESIKKLLASPKFSFCFDGLHGVAGAYAKRMFVDELGASESSLLNCVPKEDFGGGHPDPNLTYAKELVDRMGLGKTSNVEPPEFGAAADGDADRNMVLGKRFFVTPSDSVAIIAANAVQSIPYFASGLKGVARSMPTSAALDVVAKNLNLKFFEVPTGWKFFGNLMDAGMCSVCGEESFGTGSDHIREKDGIWAVLAWLSILAYKNKDNLGGDKLVTVEDIVLQHWATYGRHYYTRYDYENVDAEAAKELMANLVKMQSSLSDVNKSIKEIQPTVADVVSADEFEYKDPVDGSVSKHQGIRYLFGDGSRLVFRLSGTGSVGATIRIYIEQYEKDSSKTGRASSDALSPLVDVALKFSKIKEYTGRSAPTVIT									
	1	Phosphoglucomutase [Triticum aestivum]	gi|18076790	62749.8	5.66	26	879	100	673	100
	2	Unnamed protein product [Triticum aestivum]	gi|300681516	13915.9	5.92	5	32	0		
	3	Nuclear transcription factor 1 [Triticum aestivum]	gi|313575761	22026.9	8.11	6	29	0		
	4	Serine/threonine protein kinase [Triticum aestivum]	gi|14571547	45928.1	8.39	7	27	0		
	5	Mitogen-activated protein kinase MAPK1d [Triticum aestivum]	gi|84795224	42746.3	8.82	7	24	0		
	6	Ribulose bisphosphate carboxylase small chain clone 512	gi|132107	13046.5	5.84	4	24	0		
	7	Protein serine/threonine kinase [Triticum aestivum]	gi|310723069	51387.9	8.09	7	24	0		
	8	pTACR7 [Triticum aestivum]	gi|12656895	11070.4	10.99	5	23	0		
	9	Resistance-related receptor-like kinase [Triticum aestivum]	gi|116008311	72503.7	7.10	8	22	0		
	10	Glycosyltransferase, HGA-like [Triticum aestivum], putative,expressed	gi|300681530	68832	6.38	8	22	0		
30	MSPQTETKAGVGFKAGVKDYKLTYYTPEYETKDTDILAAFRVSPQPGVPPEEAGAAVAAESSTGTWTTVWTDGLTSLDRYKGRCYHIEPVAGEDSQWICYVAYPLDLFEEGSVTNMFTSIVGNVFGFKALRALRLEDLRIPPTYSKTFQGPPHGIQVERDKLNKYGRPLLGCTIKPKLGLSAKNYGRACYECLRGGLDFTKDDENVNSQPFMRWRDRFVFCAEAIYKSQAETGEIKGHYLNATAGTCEEMIKRAVFARELGVPIVMHDYLTGGFTANTTLAHYCRDNGLLLHIHRAMHAVIDRQKNHGMHFRVLAKALRMSGGDHIHSGTVVGKLEGEREMTLGFVDLLRDDFIEKDRARGIFFTQDWVSMPGVIPVASGGIHVWHMPALTEIFGDDSVLQFGGGTLGHPWGNAPGAAANRVALEACVQARNEGRDLAREGNEIIRAACKWSPELAAACEVWKAIKFEFEPVDTIDK									
	1	Ribulose-1,5-bisphosphate carboxylase/oxygenase large subunit [Triticum aestivum]	gi|14017580	52817.4	6.22	20	592	100	406	100
	2	Ribulose-1,5-bisphosphate carboxylase/oxygenase large subunit [Triticum aestivum]	gi|32966580	52757.3	6.22	19	578	100	406	100
	3	Ribulose-1,5-bisphosphate carboxylase/oxygenase large subunit [Triticum aestivum]	gi|12344	46972.5	6.60	17	503	100	353	100
	4	Ribulose-1,5-bisphosphate carboxylase/oxygenase large subunit [Triticum aestivum]	gi|339742637	21900	7.57	8	269	100	198	100
	5	Ribulose-1,5-bisphosphate carboxylase/oxygenase large subunit, partial [Triticum aestivum]	gi|313664567	22368.2	5.78	8	268	100	198	100
	6	VIVIPAROUS1 protein [Triticum aestivum]	gi|7801372	73395.2	8.63	8	34	0	12	0
	7	Viviparous 1 protein [Triticum aestivum]	gi|219819649	72598.7	8.16	8	34	0	12	0
	8	VIVIPAROUS1 protein [Triticum aestivum]	gi|94962615	72756.8	8.44	8	34	0	12	0
	9	VIVIPAROUS1 protein [Triticum aestivum]	gi|7801376	73086.9	8.44	7	28	0	12	0
	10	Starch synthase IIIb precursor [Triticum aestivum]	gi|189096677	143134.1	5.13	10	27	0		
	MADANAPFALVSRLSPAARLPIRAWRAARPAPLSTGGRTRPLSVASAAQENRDNSVDVQVSQAQNAGNQQGNAVQRRPRRAGFDISPFGLVDPMSPMRTMRQMLDTMDRLFDDAVGFPTARRSLAAASEMPRMPWDIMEDDKEVKMRFDMPGLSREEVKVMVEGDALVIRGEHKKEAGEGQGEAAEGGDGWWKERSVSSYDMRLALPDECDKSQVRAELKNGVLLVSVPKRETERKVIDVKVQ									

31	1	Heat shock protein HSP26 [Triticum aestivum]	gi|4028569	26857.5	7.85	5	45	83.264	28	97.862
	2	Phospoenolpyruvate carboxylase [Triticum aestivum]	gi|3341490	110082.5	5.61	7	33	0	23	92.327
	3	Dehydroascorbate reductase [Triticum aestivum]	gi|259017810	23343.1	5.88	6	31	0		
	4	Cold-regulated protein 15b [Arabidopsis thaliana]	gi|15227952	14951.7	6.62	5	30	0		
	5	DEP1 [Triticum aestivum]	gi|208293840	31669.2	8.29	8	28	0		
	6	Ribulose-bisphosphate carboxylase (EC 4.1.1.39) small chain precursor (clone 234) - wheat (fragment)	gi|82619	15344.9	8.95	5	27	0		
	7	P450 [Triticum aestivum]	gi|51572463	59563.8	7.10	9	25	0		
	8	Serine/threonine protein kinase Stpk-D [Triticum aestivum]	gi|333385001	44526.8	7.63	7	25	0		
	9	PDI-like protein [Triticum aestivum], putative	gi|299469376	40249.9	6.17	7	25	0		
	10	40S ribosomal protein [Triticum aestivum]	gi|307941401	14750.9	9.90	5	24	0		
32	MAQAVVPAMQCQVGVRGRSAVPARQPAGRVWGVRRTARAASGFKVLALGPETTGVIQRMQQLLDMDTTPFTDKIIAEYIWVGGSGIDLRSKSRTISKPVEDPSELPKWNYDGSSTGQAPGEDSEVILYPQAIFKDPFRGGNNIL<br>VICDTYTPQGEPIPTNKRHMAAQIFSDPKVTSQVPWFGIEQEYTLMQRDVNWPLGWPVGGYPGPQGPYYCAVGSDKSFGRDISDAHYKACLYAGIEISGTNGEVMPGQWEYQVGPSVGIDAGDHIWASRYILERITEQAGVVL<br>TLDPKPIQGDWNGAGCHTNYSTLSMREDGGFDVIKKAILNLSLRHDLHIAAYGEGNERRLTGLHETASISDFSWGVANRGCSIRVGRETEAKGKGYLEDRRPASNMDPYTVTALLAETTILWEPTLEAEALAAKKLALKV									
	1	Glutamine synthetase 2 [Triticum aestivum], plastid	gi|251832986	46659.4	5.75	22	891	100	699	100
	2	Glutamine synthetase 2 [Triticum aestivum], plastid	gi|251832993	46643.4	5.75	22	860	100	668	100
	3	Glutamine synthetase isoform GS2b [Triticum aestivum], plastid	gi|71362638	46615.5	6.04	19	674	100	522	100
	4	Glutamine synthetase isoform GS2a [Triticum aestivum], plastid	gi|71362455	46659.3	5.42	18	332	100	197	100
	5	Glutamine synthetase isoform GSr1 [Triticum aestivum]	gi|40317416	38705.3	5.35	3	156	100	146	100
	6	Glutamine synthetase isoform GS1c [Triticum aestivum]	gi|71361904	39216.6	5.41	3	154	100	144	100
	7	Glutamine synthetase isoform GSr2 [Triticum aestivum]	gi|40317418	38633.2	5.34	2	153	100	146	100
	8	Glycosyltransferase, HGA-like [Triticum aestivum], putative,expressed	gi|300681530	68832	6.38	11	43	75.246		
	9	Putative protein [Triticum aestivum]	gi|283099379	57999.7	5.26	8	29	0		
	10	Glutathione transferase [Triticum aestivum]	gi|20067419	24995.9	6.35	3	28	0	20	88.594
33	MAIPANAAATSLSFPVIHMEKLETEERGAAMEVIRDACENWGFFELLNHGISHELMDEVERVSKAHYAACREEQFKEFAARTLEAGEKGADVKDVDWESTFFVRHLPASNLADLPNLDHHYRQVMKEFASEIEKLAEKVLDL<br>LCENLGLEEGYLKRAFTGSNGPTFGTKVSSYPPCPRPDLVDGLRAHTDAGGVILLFQDDQVSGLQLLKDGAWVDVPPMRHAVVVNIGDQLEVITNGRYKSVMHRVLTRPDGNRMSIASFYNPGADAVIFPAPALMGAAEAAG<br>KNEGEEGTAEYPSFVFEDYMNLYLRHKFEAKEPRFEAMKVDAAPIATV									
	1	Predicted protein [Hordeum vulgare subsp. vulgare]	gi|326512860	36765.2	4.96	11	132	100	71	99.977
	2	Predicted protein [Hordeum vulgare subsp. vulgare]	gi|326522108	36658.3	5.08	9	114	100	71	99.977
	3	uncharacterized protein LOC100191321 [Zea mays]	gi|212274981	35273.5	4.89	5	77	97.739	59	99.618
	4	unknown [Zea mays]	gi|194701382	21774	5.12	3	71	91.792	59	99.618
	5	hypothetical protein VOLCADRAFT_86598 [Volvox	gi|302829949	80365.4	5.24	8	62	31.725	47	93.324
	6	predicted protein [Micromonas pusilla CCMP1545]	gi|303285780	66000.5	5.51	15	62	31.725		
	7	ACC oxidase [Alstroemeria peruviana]	gi|50845230	8075.3	5.98	2	55	0	43	84.314
	8	phenylalanine ammonia-lyase [Ginkgo biloba]	gi|156118600	79058.2	5.96	14	53	0		
	9	calmodulin-binding motif family protein [Micromonas pusilla CCMP1545]	gi|303291411	49738	10.36	7	52	0	38	50.053
	10	Os03g0153500 [Oryza sativa Japonica Group]	gi|297600348	43468.2	6.34	7	51	0		
34	MASLADLVNLDLSDCTDKIIVEYLWVGGSGIDIRSKARTVNGPITDASQLPKWNYDGSSTGQAPGEDSEVILYPQAIFKDPFRRGDNLLVMCDCYTPQGVPIPTNKRHNAAKIFNTPKVAAEETWYGIEQEYTLLQKDVNWPLG<br>WPIGGYPGPQGPYYCAAGADKAFGRDIVDAHYKACLYAGINISGINGEVMPGQWEFQVGPSVGIAASDQLWVARYILERITEVAGVVLSLDPKPIPGDWNGAGAHTNYSTKSMREAGGYEVIKTAIEKLGKRHAQHIAAYGEG<br>NERRLTGHHETADINTFKWGVADRGASVRVGRDTEKDGKGYFEDRRPASNMDPYVVTSMIAETTLLL									
	1	glutamine synthetase isoform GSr1 [Triticum aestivum]	gi|40317416	38705.3	5.35	14	711	100	608	100
	2	glutamine synthetase isoform GSr2 [Triticum aestivum]	gi|40317418	38633.2	5.34	14	614	100	511	100
	3	glutamine synthetase isoform GS1a [Triticum aestivum]	gi|71361900	39172.6	5.41	11	205	100	140	100
	4	glutamine synthetase isoform GS1b [Triticum aestivum]	gi|71361902	39188.6	5.41	10	196	100	140	100
	5	Glutamine synthetase isoform GS2c [Triticum aestivum], plastid	gi|71362640	46673.4	5.75	4	154	100	142	100
	6	yellow rust resistance protein, partial [Triticum aestivum]	gi|375127084	17187.8	6.36	5	30	0		
	7	Chromomethylase MTH2 [Triticum aestivum], putative	gi|16755865	10159.1	8.80	5	30	0		
	8	glycosyltransferase, HGA-like, putative,expressed	gi|300681530	68832	6.38	9	29	0		
	9	Chromomethylase MTH1 [Triticum aestivum], putative	gi|16755863	12117.2	9.60	5	28	0		
	10	polyphenol oxidase [Triticum aestivum]	gi|118131878	25104	5.26	5	26	0		

35	MADGEDIQPLVCDNGTGMVKAGFAGDDAPRAVFPSIVGRPRHTGVMVGMGQKDAYVGDEAQSKRGILTLKYPIEHGIVSNWDDMEKIWHHTFYNELRVAPEEHPVLLTEAPLNPKANREKMTQIMFETFNVPAMYVAIQAV LSLYASGRTTGIVLDSGDGVSHTVPIYEGYALPHAILRLDLAGRDLTDSLMKILTERGYSFTTSAEREIVRDIKEKLAYVALDYEQELETAKNSSSVEKSYELPDGQVITIGAERFRCPEVLFQPSMIGMESSGIHETTYNSIMKCDV DIRKDLYGNIVLSGGTTMFPGIADRMSKEITALAPSSMKIKVVAPPERKYSVWIGGSILASLSTFQQMWISKDEYDESGPAIVHRKCF									
	1	Actin-97-like [Brachypodium distachyon], Ppredicted	gi|357135037	41700.9	5.23	16	752	100	634	100
	2	actin [Triticum aestivum]	gi|255684860	28061.3	5.34	11	666	100	583	100
	3	actin-like protein [Triticum aestivum]	gi|37694729	21402.8	4.84	5	410	100	381	100
	5	actin [Triticum aestivum]	gi|371927227	14740.3	5.16	5	33	0		
	6	reverse transcriptase [Triticum aestivum]	gi|15723989	29343.8	8.53	7	27	0		
	7	abscisic acid-induced protein [Triticum aestivum]	gi|32400834	10950.2	11.74	4	24	0		
	8	RING finger protein [Triticum aestivum]	gi|353167263	16927.8	8.04	3	23	0	12	22.746
	9	aestivum stearoyl-ACP desaturase [Triticum aestivum]	gi|319739542	44610.4	6.05	6	23	0		
	10	SNF2 family N-terminal domain protein, partial [Triticum aestivum]	gi|129282525	7972.2	5.76	3	23	0		
36	MAAVDTFLFTSESVNEGHPDKLCDQISDAVLDACLAEDPDSKVACETCTKTNMVMVFGEITTKANVDYEKIVRDTCRGIGFVSNDVGLDADHCKVLVNIEQQSPDIAQGVHGHFTKRPEEIGAGDQGHMFGYATDETPEFMPL SHVLATKLGARLTEVRKNATCPWLRPDGKTQVTVEYHNDNGAMVPIRVHTVLISTQHDETVTNDEIAADLKEHVIKPVIPEQYLDENTIFHLNPSGRFVIGGPHGDAGLTGRKIIIDTYGGWGAHGGGAFSGKDPTKVDRSGAY VARQAAKSIVASGIARRCIVQVSYAIGVPEPLSVFVDTYGTGKIPDKEILEIVKENFDFRPGMIIINLDLKRGGKGRYLKTAAYGHFGREGADFTWEVVKPLKWEKPSA									
	1	S-adenosylmethionine synthase/Methionine adenos	gi|223635315	43152.7	5.55	4	109	100	98	100
	2	Axi 1 like protein [Triticum aestivum], putative	gi|300681428	48037.3	9.23	9	32	0		
	3	Axi 1 like protein [Triticum aestivum], putative	gi|300681427	61972.6	9.45	10	32	0		
	4	Rps3 [Triticum aestivum]	gi|291498608	64167.2	10.41	10	30	0		
	5	Rps3 [Triticum aestivum]	gi|81687782	64135.2	10.39	10	29	0		
	6	Starch synthase IIIb precursor [Triticum aestivum]	gi|189096677	143134.1	5.13	9	28	0	17	74.788
	7	Fatty acyl coA reductase [Triticum aestivum]	gi|22003090	57224.5	7.31	6	27	0	10	0
	8	Dsulfide oxidoreductase [Triticum aestivum], putative, expressed	gi|300681588	57533.6	6.30	6	26	0	10	0
	9	Tsn1 [Triticum durum]	gi|296033920	168126.7	6.62	15	26	0		
	10	Pyridoxine biosynthesis protein [Triticum aestivum]	gi|74026503	33277.1	6.40	6	25	0	2	0
37	MAAAKSVDRLAQRLVTPAEPTPAGPLRLSWLDRYPTQMALIESLHVFKPAPDRDGGDAGPAGTIERALAQALVQYYPLAGRLGFTEEGGLLQVDCGGGGSGVWFTEAAAGCALEDVEYLEHPMMIAKDELLPPTPAQEEDA RKLVLLVQVTTFACGGFVVGFRFSHAVADGPGAAQFMAAVGELARGRTAEGLTVEPQWGREAIPDPAGAVIGSLPSPAGAKRLEYLAMDISADYINHFKSQYNSSHTGSWCSAFEVLVAKAWQSRTRAAGFEEDSIVHLCFAM NARPLLHASLPRAGAGFYGNCYYIMRVSAPAGKVAGSTIPEVVKIIKDGKRRMPSEFGRWATGEAGADGGVDPYQITSDYRTLLVSDWTRLGFAEVDYGWGPPAHVVPLTNLDYIATCILVKPWAHKPGARLITQCVTPDRIAA FHEGMLDMN									
	1	S-adenosylmethionine synthase	gi|223635315	43152.7	5.55	16	394	100	284	100
	2	Mechanosensitive ion channel [Triticum aestivum]	gi|86439721	34712.9	8.46	8	30	0		
	3	Glutamyl tRNA reductase [Triticum aestivum]	gi|354681872	57572	8.57	6	26	0	6	0
	4	RAD51D protein [Triticum aestivum]	gi|197092365	26608.1	9.48	6	25	0		
	5	Ribulose-1,5-bisphosphate carboxylase/oxygenase small	gi|11990893	19500.8	9.06	5	25	0		
	6	Pre-mRNA processing factor [Triticum aestivum], putative	gi|68035888	53987.6	9.67	8	24	0		
	7	MYB-related protein [Triticum aestivum]	gi|359950770	66124	9.47	8	24	0		
	8	Ribulose-1,5-bisphosphate carboxylase/oxygenase small subunit [Triticum aestivum]	gi|11990897	19447.8	8.80	5	24	0		
	9	Acyl transferase 5 [Triticum aestivum], putative	gi|151175359	46832.5	5.53	1	24	0	24	96.447
	10	Thylakoid ascorbate peroxidase [Triticum aestivum]	gi|46095321	47948.3	7.57	5	24	0	10	0
38	MAAVDTFLFTSESVNEGHPDKLCDQISDAVLDACLAEDPDSKVACETCTKTNMVMVFGEITTKANVDYEKIVRDTCRGIGFVSNDVGLDADHCKVLVNIEQQSPDIAQGVHGHFTKRPEEIGAGDQGHMFGYATDETPEFMPL SHVLATKLGARLTEVRKNATCPWLRPDGKTQVTVEYHNDNGAMVPIRVHTVLISTQHDETVTNDEIAADLKEHVIKPVIPEQYLDENTIFHLNPSGRFVIGGPHGDAGLTGRKIIIDTYGGWGAHGGGAFSGKDPTKVDRSGAY VARQAAKSIVASGIARRCIVQVSYAIGVPEPLSVFVDTYGTGKIPDKEILEIVKENFDFRPGMIIINLDLKRGGKGRYLKTAAYGHFGREGADFTWEVVKPLKWEKPSA									
	1	S-adenosylmethionine synthase	gi|223635315	43152.7	5.55	11	273	100	214	100
	2	Ent-kaurene synthase [Triticum aestivum]	gi|326367366	95062.1	6.40	11	31	0		
	3	Trehalose-6-P synthase [Triticum aestivum]	gi|206600899	96698.6	5.97	6	29	0	20	88.125
	4	Alcohol dehydrogenase ADH1A [Triticum aestivum]	gi|119388723	40988.5	6.15	7	29	0		
	5	CBFIIIc-3.1 [Triticum aestivum]	gi|117653891	24876.1	4.86	4	28	0	9	0
	6	Vacuolar ATPase subunit F [Triticum aestivum]	gi|94537548	14370.5	5.33	4	28	0		
	7	Unnamed protein product [Triticum aestivum]	gi|300681470	24476.1	6.77	6	25	0		

	8	MYB-related protein [Triticum aestivum]	gi|359950770	66124	9.47	8	24	0		
	9	Powdery mildew resistance protein PM [Triticum aestivum]	gi|62511923	75144.9	7.62	7	24	0	9	0
	10	Alcohol dehydrogenase ADH1D [Triticum aestivum]	gi|119388725	40989.5	6.03	6	22	0		
	MGSIAAGADEDACMYALQLVSSSILPMTLKNAIELGLLETLMAAGGKFLTPAEVAAKLPSAANPEAPDMVDRMLRLLASYNVVSCRTEEGKDGRLSRRYGAAPVCKYLTPNEDGVSMSALALMNQDKVLMESWYYLKDAVLDGGIPFNKAYGMSAFEYHGTDPRFNRVFNEGMKNHSIIITKKLLESYKGFEGLGTLVDVGGGVGATVAAITAHYPTIKGINFDLPHVISEAPPFPGVTHVGGDMFQKVPSADAILMKWILHDWSDEHCATLLKNCYDALPAHGKVVLVECILPVNPEATPKAQGVFHVDMIMLAHNPGGRERYEREFEALAKGAGFAAMKTTYIYANAWAIEFTK									
	1	Flavone O-methyltransferase 1/Caffeic acid O-methyltransferase	gi|75147302	38834.6	5.75	15	732	100	615	100
	2	Caffeic acid 3-O-methyltransferase [Triticum aestivum]	gi|145321007	38573.4	5.71	12	442	100	364	100
	3	Caffeic acid O-methyltransferase [Triticum aestivum]	gi|33323468	22028	6.04	7	218	100	174	100
39	4	O-methytransferase 4 [Triticum aestivum]	gi|145693798	38430.3	5.60	8	212	100	174	100
	5	O-methyltransferase 5 [Triticum aestivum]	gi|145693796	38983.6	5.37	4	108	100	96	100
	6	Small heat shock protein Hsp23.6 [Triticum aestivum]	gi|4138871	23622	5.25	5	25	0		
	7	Ribulose-1,5-bisphosphate carboxylase/oxygenase small	gi|11990893	19500.8	9.06	5	24	0		
	8	Ribulose bisphosphate carboxylase	gi|132107	13046.5	5.84	4	24	0		
	9	Cytochrome b6-f complex iron-sulfur subunit, chloroplastic	gi|68566191	23711	8.47	5	23	0		
	10	MADS-box transcription factor TaAGL26 [Triticum aestivum]	gi|95981884	24101.4	7.81	5	22	0		
	MGSIAAGADEDACMYALQLVSSSILPMTLKNAIELGLLETLMAAGGKFLTPAEVAAKLPSAANPEAPDMVDRMLRLLASYNVVSCRTEEGKDGRLSRRYGAAPVCKYLTPNEDGVSMSALALMNQDKVLMESWYYLKDAVLDGGIPFNKAYGMSAFEYHGTDPRFNRVFNEGMKNHSIIITKKLLESYKGFEGLGTLVDVGGGVGATVAAITAHYPTIKGINFDLPHVISEAPPFPGVTHVGGDMFQKVPSADAILMKWILHDWSDEHCATLLKNCYDALPAHGKVVLVECILPVNPEATPKAQGVFHVDMIMLAHNPGGRERYEREFEALAKGAGFAAMKTTYIYANAWAIEFTK									
	1	Flavone O-methyltransferase 1/Caffeic acid O-methyltransferase	gi|75147302	38834.6	5.75	18	711	100	556	100
	2	Caffeic acid 3-O-methyltransferase [Triticum aestivum]	gi|145321007	38573.4	5.71	14	508	100	409	100
	3	O-methytransferase 4 [Triticum aestivum]	gi|145693798	38430.3	5.60	9	258	100	212	100
40	4	Caffeic acid O-methyltransferase [Triticum aestivum]	gi|33323468	22028	6.04	7	256	100	212	100
	5	O-methyltransferase 5 [Triticum aestivum]	gi|145693796	38983.6	5.37	6	152	100	130	100
	6	Glutathione synthetase [Triticum aestivum]	gi|33352235	52738.9	5.35	2	27	0	27	97.831
	7	Ribulose-1,5-bisphosphate carboxylase/oxygenase small	gi|11990893	19500.8	9.06	5	24	0		
	8	Ribulose bisphosphate carboxylase	gi|132107	13046.5	5.84	4	24	0		
	9	MADS-box transcription factor TaAGL26 [Triticum	gi|95981884	24101.4	7.81	5	22	0		
	10	Ribulosebisphosphate carboxylase [Triticum aestivum]	gi|755804	18119.9	8.95	4	22	0		
	MAGKRELVISFGEMLIDFVPTVAGVSLAEAPAFVKAPGGAPANVAIAVARLGGAAAFVGKLGDDEFGRMLAGILRDNGVDASAVVFDSGARTALAFVTLRADGEREFMFYRNPSADMLLTAAELNVELIKKAAVFHYGSISLIAEPCRTAHLRAMEIAKGAGALLSYDPNLREALWPSRDEARTKILSIWDQADIVKVSEVELEFLTGIDSVEDNVVMKLWRPTFKLLLVTLGDQGCKYYTKDFRGVVPSYKVQQVDTTGAGDAFVGALLRKIVHDPSSLQDQKKLVEAIKFANACGAITATKKGAIPSLPTEVEVLRLIEKA									
	1	Predicted protein [Hordeum vulgare subsp. vulgare]	gi|326489677	34762.2	5.67	12	498	100	426	100
	2	Os01g0894300 [Oryza sativa Japonica Group]	gi|297598102	34698	5.07	9	294	100	248	100
	3	Fructokinase-1	gi|158512869	34707	5.14	8	286	100	248	100
41	4	Fructokinase-1 [Zea mays]	gi|195650763	34582	5.13	7	279	100	248	100
	5	Fructokinase [Oryza sativa Japonica Group], putative	gi|57899806	27182.1	5.17	6	247	100	221	100
	6	Hypothetical protein OsI_04762 [Oryza sativa Indica Group]	gi|218189518	27191.1	5.27	5	240	100	221	100
	7	Fructokinase-1-like [Brachypodium distachyon], predicted	gi|357126300	34650.3	5.79	8	211	100	174	100
	8	Predicted protein [Hordeum vulgare subsp. vulgare]	gi|326518162	34594.8	4.76	7	204	100	174	100
	9	Predicted protein [Hordeum vulgare subsp. vulgare]	gi|326494746	38124.6	5.66	9	143	100	101	100
	10	Fructokinase-2 [Vitis vinifera], predicted	gi|225433918	35177.2	5.13	8	111	100	74	99.984
	MAIKRTKAEKKVAYDKKLCQLLDEYTKVLIAVADNVGSNQLQEIRKGLRGDSIVLMGKNTLIRRCIKVHADNTGNKEFLELMPLLVGNVGLIFTKGDLKEVREEVAKYKVGAPARVGLVAPVDVVVPPGNTGLDPSQTSFFQVLNIPTKINKGTVEIITPVELIKKGDKVGSSESALLAKLGIRPFSYGLVITNVYDSGSVFSPEVLDLTEDDLMEKFASGVSMVASVSLAISYPTIAAAPHMFLNGYKNVLAVAVETEYSYPHADKIKEYLKDPSKFAVAAPVAADSGAAAPSAAKEEEKKEEPEEESDGDLGMSLFD									
	1	Os08g0130500 [Oryza sativa Japonica Group]	gi|115474653	34356.1	5.38	3	92	100	81	100
	2	MYB-related protein [Triticum aestivum]	gi|359952782	48051.4	8.93	8	33	0		
	3	Abscisic acid-induced protein [Triticum aestivum]	gi|32400834	10950.2	11.74	5	32	0		
42	4	Annexin [Triticum aestivum]	gi|38606205	35416.1	9.01	8	32	0		

	5	Polyprotein [Triticum aestivum], putative	gi|217426769	28749.3	5.29	7	30	0		
	6	Ribulose-1,5-bisphosphate carboxylase/oxygenase small	gi|4038673	18533.4	8.83	5	27	0		
	7	P450 [Triticum aestivum]	gi|48762803	59137.5	6.41	8	26	0		
	8	P450 [Triticum aestivum]	gi|48526685	59236.6	6.50	8	26	0		
	9	P450 [Triticum aestivum]	gi|62148970	59289.7	6.61	8	25	0		
	10	cytochrome P450 [Triticum aestivum]	gi|26655528	59519.8	6.75	8	25	0		
	MASRMALRPHAVTPRLAAGRGGRRNGSVRVLAVASCASAKIDSKKTFAPPKQVHVQVTHSMPPQKMDIFQSLYGWARDNLKLHLKPVEKCWQPQDFLPDPPSDGFHDEVKELRERAKENPDDYLVCLFGDMITEEALPTYQ TMLNTLDGVRDETGASPTAWAVWTRAWTAEENRHGDLLNKYLYLTGRVDMRQIEKTIQYLIGSGMDPRTENNPYLGFIYTSFQERATFISHGNTARHAKDFGDLKLAQICGIIASDEKRHETAYTKIVEKLFEIDPDGTVLALAD MMRKKIAMPAHLMFDGQDEKLFDHFSMVAQRLGVYTARDYADILEFLVGRWKVPELTGLSGEGHKAQDYLCTLAGRIRKLDERAQSRAKQAGKMPFSWVYGREVQM									
	1	Stearoyl-ACP desaturase [Triticum aestivum]	gi|319739540	44426.4	8.15	14	228	100	155	100
	2	ADP-glucose pyrophosphorylase small subunit, plastid	gi|224021585	56253.8	6.40	9	30	0		
	3	Puroindoline a [Triticum aestivum]	gi|149212447	16366	8.72	6	30	0		
43	4	Ribulose-bisphosphate carboxylase (EC 4.1.1.39) small	gi|82619	15344.9	8.95	5	30	0		
	5	WALI7 [Triticum aestivum]	gi|257135793	28053.7	6.99	6	28	0		
	6	SUSIBA2-like protein [Triticum aestivum]	gi|34329333	22543.2	7.16	5	24	0		
	7	Puroindoline=basic cystine-rich protein [Triticum	gi|408873	12920.2	8.76	5	24	0		
	8	14-3-3 protein [Triticum aestivum]	gi|40781605	29273.7	4.83	6	24	0		
	9	WRKY45-like transcription factor [Triticum aestivum]	gi|341864987	31615.3	5.92	6	23	0		
	10	Protein kinase [Triticum aestivum]	gi|110341803	61660.4	9.12	9	23	0		
	MADAATIAKLEEGFKKLEAATDCKSLLKKYLTKDIFDSLKAKKTGLGATLLDVIQSGVENLDSGVGIYAPDAEAYSLFAPLFDPIIEDYHKGFKQTDKHPAKDFGDVSKFINVDPEGTFVISTRVRCGRSMEGYPFNPCLTEAQY KEMEEKVSSTLSGLEGELKGSYFPLTGMTKEVQQKLIDDHFLFKEGDRFLQAANACRYWPAGRGIYHNDNKTFLVWCNEEDHLRIISMQMGGDLGQVYRRLVSAVNDIEKRVPFSHHDRLGFLTFCPTNLGTTVRASVHIKLP KLAANREKLEEVAAKFSLQVRGTRGEHTEAEGGIYDISNKRRMGLTEFQAVKEMQDGILELIKIEKEM									
	1	Uncharacterized protein LOC100273160 [Zea mays]	gi|226528643	39942.3	6.05	10	103	99.995	50	94.629
	2	Predicted protein [Physcomitrella patens subsp. patens]	gi|168042645	12933.5	6.97	6	56	0		
	3	Maturase K [Tachigali sp. Clarke 7212]	gi|183529279	59810.3	9.33	13	53	0		
44	4	Predicted protein [Physcomitrella patens subsp. patens]	gi|168003694	13992	6.20	6	52	0		
	5	Exportin-1-like isoform 2 [Glycine max], predicted	gi|356543440	122123.4	5.35	17	50	0		
	6	Exportin-1-like isoform 3 [Glycine max], predicted	gi|356543442	122492.5	5.29	17	50	0		
	7	Predicted protein [Populus trichocarpa]	gi|224144521	18696.2	6.89	8	50	0		
	8	Hypothetical protein OsI_27441 [Oryza sativa Indica	gi|218200310	67755.7	9.85	13	50	0		
	9	Predicted protein [Chlamydomonas reinhardtii]	gi|159465393	56157.3	11.93	12	49	0		
	10	Predicted protein [Populus trichocarpa]	gi|224079477	11660.8	9.05	7	49	0		
	MRSSLLRSASQHLRRRGYASAAGGQPERKVAILGAAGGIGQPLALLMKLNPLVSSLSLYDIAATPGVAADVSHINSPALVKGFVGDDQLGEALEGADLVIIPAGVPRKPGMTRDDLFKINAGIVKGLCTAISKYCPNALVNMISNP VNSTVPIAAEVFKKAGTYDEKKLFGVTTLDVVRAKTFYAGKANVPVTGVNVPVVGGHAGITILPLFSQATPSSNALSHEDLKALTKRTQDGGTEVVEAKAGKGSATLSMAYAGAVFGDACLKGLNGVPDIIECSFVQSTVTDL PFFASKVRLGKNGVEEVIGLGELSAFEKEGLESLKGELMSSIEKGIKFAQEN									
	1	Predicted protein [Hordeum vulgare subsp. vulgare]	gi|326493440	35844.9	8.20	8	593	100	558	100
	2	Malate dehydrogenase 1,mitochondrial-like [Brachypodium distachyon]	gi|357132456	35371.7	8.54	6	364	100	342	100
	3	Malate dehydrogenase [Micromonas sp. RCC299]	gi|255073915	33424.3	4.95	7	133	100	104	100
45	4	Os05g0574400 [Oryza sativa Japonica Group]	gi|115465579	35413.7	8.22	7	133	100	104	100
	5	Predicted protein [Populus trichocarpa]	gi|224116458	22240.8	9.10	5	127	100	104	100
	6	Malate dehydrogenase, mitochondrial;	gi|126896	36178.2	8.88	6	125	100	104	100
	7	Malate dehydrogenase [Pinus pinaster]	gi|332591472	36954.4	8.90	6	125	100	104	100
	8	Malate dehydrogenase, putative [Ricinus communis]	gi|255563084	35850.8	8.47	6	125	100	104	100
	9	Hypothetical protein SORBIDRAFT_03g029570	gi|242053811	35613.8	7.63	6	125	100	104	100
	10	Os01g0649100 [Oryza sativa Japonica Group]	gi|115438875	35438.6	8.74	6	124	100	104	100
	LLHRAFSVFLFNSKPELLLQQRSATKVTFPLVWTNTCCSHPLYRESELIQENFLGVRNAAQRKLLDELGIPAEDVPVDQFTPLGRMLYKAPSDGKWGEHELDYLLFIVRDVKLVPNPDEVADVKYVSREQLRELIQQADAGEGG VKLSPWFRLVVDNFLMGWWEHLEKGTLAEAVDMETIHKLK									
	1	Isopentenyl pyrophosphate isomerase [Triticum aestivum]	gi|193072753	21147	5.41	8	283	100	226	100
	2	Histone deacetylase [Triticum aestivum]	gi|52548252	50933.8	5.31	8	30	0		

46	3	UDP-glucoronosyl/UDP-glucosyl transferase protein	gi|171674071	54844.2	5.12	9	29	0		
	4	Serine/threonine protein kinase Stpk-A [Triticum	gi|333384999	44613.9	7.63	7	27	0		
	5	Alcohol dehydrogenase ADH3-1 [Triticum aestivum]	gi|119388711	40982.6	5.91	7	27	0		
	6	Glycosyltransferase, HGA-like, putative,expressed	gi|300681530	68832	6.38	9	27	0		
	7	Ornithine carbamoyl transferase [Triticum aestivum]	gi|318086913	3072.4	4.57	3	26	0		
	8	Cold acclimation protein WCOR615 [Triticum aestivum]	gi|1657857	17773.5	4.92	5	24	0		
	9	Tsn1 [Triticum durum]	gi|296033920	168126.7	6.62	14	24	0		
	10	Ribulose bisphosphate carboxylase small chain clone 512	gi|132107	13046.5	5.84	4	24	0		
47	MADEYGRSGAYGRSGTDDYETGYKKSGTDDYDGSGAGGYNKSAGDDEYSGGAGYKKSGAGADDYDGGYKKSTHEDYGRNKSATDDYDRSGAGGYKKSSGDDEYSGGGYKKSGGDEEYKKSTGDDDEYSGGGYKKSTPGGDDDESDKYRKEEKEHKRKEHLGEMGTLAAGAFAMYEKHQAKKDPENAHRHKIEEEVAAVAAVGSGGYAFHEHHEKKESREDAEEDEESGRGEGKKKHHFFG									
	1	Uncharacterized protein LOC100822602 [Brachypodium distachyon], predicted	gi|357163453	25950.6	5.11	4	231	100	215	100
	2	Abscisic stress ripening [Musa acuminata AAA Group]	gi|269852824	16707.8	5.70	2	125	100	116	100
	3	Hypothetical protein SELMODRAFT_138488	gi|302825464	29513.5	8.66	10	60	5.754		
	4	Hypothetical protein SELMODRAFT_428029 [Selaginella moellendorffii]	gi|302815458	40823.6	6.49	9	53	0		
	5	OSJNBb0034I13.10 [Oryza sativa Japonica Group]	gi|21741247	157623.3	8.59	20	52	0		
	6	NAD(P)H-dependent, probably, predicted	gi|225424504	35573.4	5.66	10	51	0		
	7	Uncharacterized protein LOC100855072 [Vitis vinifera], predicted	gi|359473529	100172.1	6.03	16	49	0		
	8	Hypothetical protein ARALYDRAFT_895672 [Arabidopsis lyrata subsp. lyrata]	gi|297842563	12568.4	8.46	8	49	0		
	9	Transposon protein, putative, CACTA, En/Spm sub-class [Oryza sativa Japonica Group]	gi|77551246	75968.9	8.64	15	48	0		
	10	Unknown [Zea mays]	gi|238007756	47104.8	7.78	11	47	0		
48	MAPKDRILVLGPTGAIGRHIVWASVKAGNPTFILVRDTPASVNKPRLVTAANPETREELIQSFQNSGVTLIQGDMNDHESLVNAIKQVDVVICSFGRLLIEDQVKIVAAIKEAGNVKRFFPSEFGLDVDRHDAAEPVREVFEEKAKIRRVIEAEGIPYTYLCCHAFTGYFLRNLAQIDITVPPRDKVFIQGDGNVKGAYITEADVGTFTIEAANDPRALNKAVHIRLPNNYLSLNDIISLWEKKIGKTLEKIYVSEEEVLKQIKETSFLNNYLLALYHSQQIKGDAVYEIDPAKDLEASEAYPHVEYSTVSEYLDQFV									
	1	Hypothetical protein SORBIDRAFT_03g008760 [Sorghum bicolor]	gi|242052385	33034.5	5.38	6	280	100	256	100
	2	Hypothetical protein SORBIDRAFT_03g029820 [Sorghum bicolor]	gi|242058197	30399.9	5.53	5	195	100	176	100
	3	Unknown [Glycine max]	gi|255648230	35643.7	5.51	6	119	100	97	100
	4	NADPH:isoflavone reductase [Glycine max]	gi|351724529	35568.5	5.30	6	119	100	97	100
	5	Isoflavone reductase homolog [Lotus japonicus]	gi|116077992	35360.7	5.69	6	119	100	97	100
	6	Isoflavone reductase-like protein [Picea sitchensis]	gi|306018397	30818.9	7.05	2	117	100	111	100
	7	Unknown [Picea sitchensis]	gi|148909885	16875.5	5.12	1	115	100	111	100
	8	Isoflavone reductase homolog IRL [Zea mays]	gi|162461348	32831.4	5.69	5	114	100	97	100
	9	Isoflavone reductase-like [Glycine max], predicted	gi|356538212	35549.7	5.51	5	113	100	97	100
	10	Isoflavone reductase/2'-hydroxyisoflavone reductase/isoflavone oxidoreductase	gi|1708425	35386.5	5.94	5	113	100	97	100
49	MDRTSQWVSSPDIPADLLIRIADDVFPLLKGVMVPKCGYIRKAVAAARGGATATVDLDLSALPGGADAFDKAARYCYGANFEISVRNAAALLCAAAFLDMHPTDGGLARRVEEFLAKVGLRTLPGAVAVLRYCEGLLPAAEEIGVVQRSADAIALRICNEVLFPTRSPPEWWTAELAALSPASFHKVITALRSELARMRAYVSGMQQQSKGSSSSRGKKGSWLRTLSRLNPFKAGIWGKDTSGIVDGKTDAMNSVKSKRRRFSIS									
	1	Root phototropism 2 [Oryza sativa Japonica Group], putative, expressed	gi|108862102	28468.9	9.65	10	70	89.179	13	0
	2	Unknown [Picea sitchensis]	gi|148907206	39010.3	6.65	11	58	0		
	3	Root phototropism 2 [Oryza sativa Japonica Group], putative, expressed	gi|77552853	60503.3	8.66	12	57	0	13	0
	4	Os12g0117400 [Oryza sativa Japonica Group]	gi|115487026	62362.2	8.55	12	57	0	13	0
	5	Os11g0118300 [Oryza sativa Japonica Group]	gi|115483869	60493.2	8.40	12	57	0	13	0
	6	Cysteine-rich receptor-like protein kinase 29-like [Vitis vinifera], predicted	gi|225452063	72932.5	5.65	14	56	0		
	7	Ring finger protein [Ricinus communis], putative	gi|255583204	39517.7	6.83	11	54	0		
	8	Hypothetical protein MTR_1g005870 [Medicago truncatula]	gi|357436077	20917.8	8.91	9	52	0		
	9	Hypothetical protein VITISV_026923 [Vitis vinifera]	gi|147791345	69798	6.17	14	52	0		
	10	Hypothetical protein OsI_34888 [Oryza sativa Indica Group]	gi|218185128	60482.2	8.25	11	51	0	13	0
	MEDINNGFKAAVAAAAGVPAADKYKTFEATFSAASNKAFAEVLKGAATGQYAAQSSSMAKLSSSLELSYKLAYDKAQGATPETKYDAYVATLTESLRVISGTLEVHSVKPAEEEVKGVPTGELKAIDQVDAAFRTAATAADAAPVNDKFTVFESSFNKAIKETTGGTYESYKFIPALEAAVKQAYAATVAAAPEVKFTVFQTALSKAINAMTQAEKDAKPAAAATATATPGASAGATAGGYKA									
	1	Group 5/9 grass pollen allergen R8-5 [Triticum turgidum subsp. durum x Secale cereale]	gi|365769201	24910.6	5.61	7	199	100	164	100
	2	30 kDa pollen allergen [Hordeum vulgare]	gi|459163	18359.6	8.68	4	182	100	164	100

50	3	Pollen allergen precursor [Hordeum vulgare]	gi|1808987	33127.1	9.43	4	176	100	164	100
	4	Predicted protein [Hordeum vulgare subsp. vulgare]	gi|326527679	30064.4	6.62	4	174	100	161	100
	5	Predicted protein [Hordeum vulgare subsp. vulgare]	gi|326534176	35730.8	6.40	7	140	100	116	100
	6	Uncharacterized protein LOC100830071 [Brachypodium distachyon], predicted	gi|357164825	36973.4	8.44	3	123	100	116	100
	7	Uncharacterized protein LOC100382145 [Zea mays]	gi|293335391	37298.5	6.49	4	123	100	112	100
	8	Hypothetical protein SORBIDRAFT_06g023220 [Sorghum bicolor]	gi|242073772	37152.5	6.56	4	122	100	112	100
	9	Hypothetical protein OsJ_15539 [Oryza sativa Japonica	gi|222629244	37523.9	6.89	2	121	100	116	100
	10	OO_Ba0013J05-OO_Ba0033A15.6 [Oryza officinalis]	gi|260447006	56654.2	9.60	2	116	100	116	100
	MVSTLMAPASRAVAGKTPFLGARPAVASPLRDVAAAASNGRISMSNELWYGPDRVKYLGPFSAQTPSYLSGEFPGDYGWDTAGLSADPEAFARNRALEVIHGRWAMLGALGCVFPEVLQKWVGVEFKEPVWFKAGAQIFSEG GLDYLGNPNLVHAQSILAVLGSQVLLMGLVEGFRINGLDGVGEGNDLYPGGQYFDPLGLADDPVTFAELKVKEIKNGRLAMFSMFGFFVQAIVTGKGPLENLFDHLDDPVANNAWVFATKFAPGS									
51	1	Chlorophyll a-b binding protein of LHCII type III, chloroplastic-like [Brachypodium distachyon]	gi|357122389	28559.4	5.00	10	475	100	412	100
	2	Chlorophyll a-b binding protein of LHCII type III, chloroplastic, Precursor	gi|115793	28740.5	4.99	9	469	100	412	100
	3	Hypothetical protein SORBIDRAFT_02g036380 [Sorghum bicolor]	gi|242045970	28716.6	5.11	6	76	97.404	48	94.948
	4	LHCII Type III chlorophyll a/b binding protein [Brassica napus]	gi|405617	24102.2	4.76	5	75	96.879	48	94.948
	5	LHCII type III chlorophyll a/b binding protein [Wolffia australiana]	gi|374434016	28504.4	5.01	5	72	94.189	48	94.948
	6	Chlorophyll A/B binding protein, putative [Ricinus communis]	gi|255569585	28855.6	5.11	5	70	89.425	48	94.948
	7	Uncharacterized protein LOC100790960 [Glycine max]	gi|359806176	28676.5	5.11	5	70	89.179	48	94.948
	8	Uncharacterized protein LOC100779387 [Glycine max]	gi|359807444	28793.5	5.11	5	70	88.927	48	94.948
	9	Chlorophyll a-b binding protein of LHCII type III [Zea mays]	gi|226509520	28984.7	5.11	5	69	87.576	48	94.948
	10	Light-harvesting chlorphyll binding protein 3 [Solenostemon scutellarioides]	gi|157987285	20879.7	4.76	4	68	85.063	48	94.948
	GTRGHAVSRDLIHWRHLPLAMVPDQWYDINGVWTGSATVLPDGSLVILYTGSTNASVQVQCLAVPADPNDSLLRNWTKYEANPILVPPPGIGDKDFRDPTTAWFDESDKTWRTSSAPRDNHGHTGIVMTYKTKDFINYELIPG LLHSVPGTGMWECIDFYPVGGADGSEELYVMKESSDDDRHDWYALGRYDAAANKYTPIDAEMDVGIGLRYDWGKFYASKTFYDPSKNRRVLWGWIGETDSERADVAKGWASLQSIPRTVELDEKTRTNLIQWPVVEIETLRI NSTDLGGTTIDTGSVLPLPLRRATQLDIEATFHLDTSAIAAVNEADVGYNCSTSGGAANRGALGPFGLLVLADGALKEQTAVYFYVSRGLDGGLQTHFCQDESRSSLAQDVVKRVVGFTVPVLDGEDLSLRVLVDHSIVESFAM GGRSTANIEGVSDGGHLRRRWRVPFQQRHRRCRHRGEARGARDGRLLQPDLHGRRCLSRLVAAINQALGPVMHIHPYYIL									
52	1	Vacuolar invertase [Triticum aestivum]	gi|3219509	56398.3	5.82	7	111	100	91	100
	2	Powdery mildew resistance protein PM3C [Triticum aestivum]	gi|82492377	159009.1	6.18	17	42	65.035	5	0
	3	Powdery mildew resistance protein PM3 variant [Triticum aestivum]	gi|225580397	157134.1	6.17	17	40	49.46		
	4	Powdery mildew resistance protein PM3 variant [Triticum aestivum]	gi|225580393	157155.1	6.15	17	39	40.62		
	5	LEA D-11 dehydrin [Triticum aestivum]	gi|21624242	12820.1	7.21	5	36	0		
	6	Pm3 [Triticum aestivum]	gi|296280016	158971	6.07	17	36	0		
	7	Powdery mildew resistance protein PM3 variant [Triticum aestivum]	gi|225580395	158673.9	6.19	17	35	0		
	8	Powdery mildew resistance protein PM3 variant [Triticum aestivum]	gi|225580391	158700.9	6.19	17	35	0		
	9	Powdery mildew resistance protein PM3 variant [Triticum aestivum]	gi|225580379	158875	6.18	16	34	0		
	10	Pm3 [Triticum aestivum]	gi|296280026	159148.2	6.30	17	33	0		
	MLVYQDKLSGDELLSDSFPYRELENGVLWEVDGHWVVQGAVDVDIGANPSAEGGGDDEGVDDQAVKVVDIVDTFRLQEQPAFDKKQFISHMKRYIKNLSAKLEGDDLDAFKKNVESATKYLLSKLKDLQFFVGESMHDDG GVVFAYYKEGAADPTFLYFAHGLKEVKC									
53	1	Translationally-controlled tumor protein [Triticum aestivum]	gi|146285306	18766.3	4.55	9	486	100	413	100
	2	Translationally-controlled tumor proteinhomolog	gi|75246527	18794.3	4.55	9	486	100	413	100
	3	Ribulose-1,5-bisphosphate carboxylase/oxygenase small subunit [Triticum aestivum]	gi|11990893	19500.8	9.06	5	26	0		
	4	Ribulose-1,5-bisphosphate carboxylase/oxygenase small subunit [Triticum aestivum]	gi|11990897	19447.8	8.80	5	26	0		
	5	NBS-LRR type RGA [Triticum aestivum]	gi|74121555	20107.3	8.82	5	25	0		
	6	NBS-LRR type RGA [Triticum aestivum]	gi|74121546	19123.7	6.95	5	25	0		
	7	Ribulose-bisphosphate carboxylase (EC 4.1.1.39) small chain precursor (clone 234) - wheat (fragment)	gi|82619	15344.9	8.95	4	23	0		
	8	Putative Zn-finger transcription factor [Triticum aestivum]	gi|60100212	19391.2	8.09	4	22	0		
	9	Glycosyltransferase, HGA-like [Triticum aestivum], putative,expressed	gi|300681530	68832	6.38	8	22	0		
	10	Bowman-Birk trypsin inhibitor-like protein [Triticum aestivum]	gi|162319710	9856.6	8.67	4	22	0		
	MEGEKNSSGDLMSSSKLVAEAARTAYEKKSVEGIDKEKVAAASADILDSAAKYGKLEDKPVGQYLEKAEEYLKQYSSGGAEKTDAPAAADAPKPDAPKEPAPAPAPAPAVEEEKSSDGFGLDDVMKGAASLSGKKGDGEEKE SGGGGFMKMAQGFMK									
	1	Predicted protein [Hordeum vulgare subsp. vulgare]	gi|326520557	16184.9	4.75	5	200	100	173	100

54	2	Predicted protein [Physcomitrella patens subsp. patens]	gi|168060960	33045.9	4.41	4	61	17.915	48	93.586
	3	Hydroxysteroid 11-beta-dehydrogenase 1-like protein-like [Brachypodium distachyon], predicted	gi|357163124	38739.3	9.02	11	57	0		
	4	Hypothetical protein OsI_28484 [Oryza sativa Indica Group]	gi|125560799	88651.5	6.02	5	56	0	48	93.542
	5	Hydroxycinnamoyl-Coenzyme A shikimate/quinate hydroxycinnamoyltransferase-like [Glycine max]	gi|356527016	52294.9	8.67	9	54	0		
	6	Hypothetical protein OsJ_15032 [Oryza sativa Japonica Group]	gi|222628982	8212.2	9.89	2	53	0	43	79.845
	7	OSJNBa0036B21.9 [Oryza sativa Japonica Group]	gi|21740895	12329.1	9.57	2	52	0	43	79.845
	8	Os01g0925800 [Oryza sativa Japonica Group]	gi|115441959	21309.6	4.95	8	52	0		
	9	Os04g0457400 [Oryza sativa Japonica Group]	gi|297602845	13572	9.73	3	51	0	43	79.845
	10	Ubiquitin-conjugating enzyme E2 27-like [Brachypodium distachyon], predicted	gi|357126604	21406.7	4.84	8	51	0		
55	MATTAMALSPSSFAGKAVKDLPSSALFGEARVTMRKTAAKAKPVSSGSPWYGSDRVLYLGPLSGDPPSYLTGEFPGDYGWDTAGLSADPETFAKNRELEVIHCRWAMLGALGCVFPELLARNGVKFGEAVWFKAGSQIFSEG<br>GLDYLGNPSLVHAQSILAIWACQVVLMGAVEGYRVAGGPLGEIVDPLYPGGSFDPLGLADDPEAFAELKVKEIKNGRLAMFSMFGFFVQAIVTGKGPLENLADHLSDPVNNNAWAFATNFVPGK									
	1	Chloroplast light-harvesting chlorophyll a/b binding protein [Triticum aestivum]	gi|302566696	28196.2	5.14	6	330	100	298	100
	2	Chlorophyll a/b-binding protein WCAB precursor [Triticum aestivum]	gi|1657859	28206.3	5.30	5	253	100	230	100
	3	Chlorophyll a-b binding protein, chloroplastic	gi|115782	28246.3	5.67	3	178	100	168	100
	4	Chlorophyll a-b binding protein [Triticum aestivum]	gi|225690798	21229.7	5.55	2	69	99.933	61	100
	5	Chlorophyll a-b binding protein [Triticum aestivum]	gi|225690800	23022.6	5.50	2	68	99.927	61	100
	6	Chlorophyll a-b binding protein [Triticum aestivum]	gi|225690804	28482.3	5.13	2	68	99.912	61	100
	7	Cold acclimation protein WCOR615 [Triticum aestivum]	gi|1657857	17773.5	4.92	6	33	0		
	8	MYB-related protein [Triticum aestivum]	gi|359952798	42359.3	9.48	6	31	0	16	73.061
	9	WRKY45-like transcription factor [Triticum aestivum]	gi|341864987	31615.3	5.92	7	28	0		
	10	Heat shock protein HSP26 [Triticum aestivum]	gi|4028573	26573.3	7.88	6	26	0		
56	MTGCVNSISPPPVTLYRHRASPSRSSFSLSGDALHSLYRHRRVSRSPSIIAPKFQIVAAEKSEPLKIMISGAPASGKGTQCELITHKYGLVHISAGDLLRAEIASGSENGRRAKEHMEKGQLVPDEIVVMMVKDRLSQTDSEQKGW<br>LLDGYPRSASQATALKGFGFQPDLFIVLEVPEEILIERVVGRRLDPVTGKIYHLKYSPPETEEIAVRLTQRFDDTEEKAKLRLKTHNQNVSDVLSMYDDITIKIEGNRSKEEVFAQIDSSLSELLQERNTAPSSLLS									
	1	Predicted protein [Hordeum vulgare subsp. vulgare]	gi|326510901	29327.9	9.00	8	189	100	145	100
	2	Predicted protein [Hordeum vulgare subsp. vulgare]	gi|326514018	29328.9	8.69	8	189	100	145	100
	3	Os08g0109300 [Oryza sativa Japonica Group]	gi|115474427	31796.9	7.71	7	132	100	101	100
	4	Hypothetical protein OsJ_25775 [Oryza sativa Japonica	gi|125601941	26078.9	9.00	6	126	100	101	100
	5	Adenylate kinase, chloroplastic-like [Brachypo, predicteddium distachyon]	gi|357139457	31257.5	8.42	6	124	100	101	100
	6	Adenosine monophosphate kinase [Arabidopsis thaliana]	gi|15238822	31432.3	7.11	3	110	99.999	101	100
	7	Predicted protein [Hordeum vulgare subsp. vulgare]	gi|326527727	12323.3	5.60	6	76	97.404	35	0
	8	Hypothetical protein ARALYDRAFT_917605 [Arabidopsis lyrata subsp. lyrata]	gi|297791885	33104.2	9.10	3	72	94.189	64	99.838
	9	Os06g0343100 [Oryza sativa Japonica Group]	gi|115467950	121329.4	6.43	11	70	90.575	54	98.507
	10	Hypothetical protein OsJ_21250 [Oryza sativa Japonica	gi|125597126	118172.8	6.28	10	67	80.309	54	98.507
57	MAMASSSGLRSCSAVGVPSLLAPSSLSGRSGLPFCAYATTSGRVTMSAEWFPGQPRPAHLDGSSPGDFGFDPLGLATVPENFERFKESEIYHCRWAMLCVPGVLVPEALGLGNWVKAQEWAALPDGQATYLGNPVPWGNLPTI<br>LAIEFLAIAFAEQQRTMEKDPEKKKYPGGAFDPLGFSKDPAKFEELKLKEIKNGRLAMLAFVGFCVQQSAYPGTGPLENPGTHLADPWHNTSATSSFPEYLPPLTGPTG									
	1	Chlorophyll a-b binding protein [Triticum aestivum]	gi|225690802	30914.2	6.12	7	102	100	68	100
	2	Chlorophyll a-b binding protein [Triticum aestivum]	gi|225690794	26958.4	5.42	6	97	100	68	100
	3	Unknown protein [Triticum aestivum]	gi|374086126	34351.5	6.41	7	27	0		
	4	RING finger protein [Triticum aestivum]	gi|353167263	16927.8	8.04	5	27	0		
	5	LEA D-11 dehydrin [Triticum aestivum]	gi|21624242	12820.1	7.21	4	25	0		
	6	Cold-regulated protein 15b [Arabidopsis thaliana]	gi|15227952	14951.7	6.62	4	25	0		
	7	Unnamed protein product [Triticum aestivum]	gi|300681470	24476.1	6.77	6	23	0		
	8	Putative protein [Triticum aestivum]	gi|283099379	57999.7	5.26	7	22	0		
	9	STK protein kinase [Triticum aestivum]	gi|71912119	19049.6	6.47	4	21	0		
	10	NAC transcription factor 2B [Triticum aestivum]	gi|293631969	36468.9	6.47	6	20	0		
	MAAATSGAVLNGLGSSFLSGGNRSQTLLATAIGGKVGAAAVTVSPRRLIVVAAAAPKKSWLPGVRGGGNLVDPEWLDGSLPGDYGFDPLGLGKDPAFLKWYREAELIHGRWAMAAVLGIFVGQAWSGVPWFEAGADPNAIA<br>PFSFGTLLGTQLILMGWVESKRWVDFFNPDSQSVEWATPWSRTSENFANATGEQGYPGGKFFDPLSLAGTIKDGVYIPDTEKLERLKLAEIKHARIAMLAMLIFYFEAGQGKTPLGALGL									
	1	Predicted protein [Hordeum vulgare subsp. vulgare]	gi|326487444	27374.1	6.75	11	475	100	402	100
	2	Hypothetical protein SORBIDRAFT_06g032690 [Sorghum bicolor]	gi|242074752	27337.2	7.90	8	367	100	325	100

58	3	Chlorophyll a-b binding protein CP24 10A, chloroplastic [Glycine max], predicted	gi|356525886	27668.3	6.15	6	301	100	274	100
	4	Uncharacterized protein LOC100527463 [Glycine max]	gi|351728089	27569.3	7.93	4	289	100	274	100
	5	Lhcb6 protein [Arabidopsis lyrata subsp. lyrata]	gi|297850008	27440.1	6.75	9	222	100	172	100
	6	Chlorophyll A/B binding protein, putative [Ricinus communis]	gi|255585659	27383	6.85	8	214	100	172	100
	7	Lhcb6 protein [Brassica rapa subsp. pekinensis]	gi|50313237	27324.1	8.07	7	206	100	172	100
	8	Chlorophyll a-b binding protein CP24 10A, chloroplastic-like [Glycine max], predicted	gi|356512075	27471	6.43	7	206	100	172	100
	9	Chloroplast a/b binding protein cab-PhE11, partial [Lolium perenne]	gi|375152108	17334.8	5.12	5	199	100	172	100
	10	H0523F07.10 [Oryza sativa Indica Group]	gi|116309995	26998.9	6.75	6	199	100	172	100
59	MMFKIWRCSDSSCSLPGDYGFDPLGLSDPEGTGGFIEPRWLAYGEIINGRFAMLGAAGAIAPEILGKAGLIPAETALPWFQTGVIPPAGTYTYWADNYTLFVLEMALMGFAEHRRLQDWYNPGSMGKQYFLGLEKGLAGSGNP AYPGGPFFNPLGFGKDEKSLKELKLKEVKNGRLAMLAILGYFIQGLVTGVGPYQNLLDHLADPVNNNVLTSLKFH									
	1	Predicted protein [Hordeum vulgare subsp. vulgare]	gi|326514018	29328.9	8.69	8	565	100	518	100
	2	Predicted protein [Hordeum vulgare subsp. vulgare]	gi|326510901	29327.9	9.00	8	445	100	398	100
	3	Chlorophyll a-b binding protein 8 [Zea mays]	gi|195613254	28965.9	8.94	6	183	100	156	100
	4	Chlorophyll a-b binding protein 8 [Zea mays]	gi|226496924	16792.6	6.82	4	177	100	156	100
	5	Chlorophyll a-b binding protein 8, chloroplastic-like [Brachypodium distachyon], predicted	gi|357139429	12632.5	8.04	3	173	100	156	100
	6	Chloroplast PSI type III chlorophyll a/b-binding protein [Helianthus annuus]	gi|159138869	13850.8	4.51	3	172	100	156	100
	7	Chain 3, The Structure Of A Plant Photosystem I Supercomplex At 3.4 Angstrom Resolution	gi|149242542	17903.3	6.95	3	169	100	156	100
	8	Chloroplast photosystem II light harvesting complex protein type III, partial [Oxytropis splendens]	gi|359754921	21416.7	5.03	3	168	100	156	100
	9	Chlorophyll a-b binding protein [Medicago truncatula]	gi|357495589	61481.6	8.73	5	167	100	153	100
	10	Light-harvesting complex I chlorophyll a/b binding protein 3 [Arabidopsis thaliana]	gi|79320443	23731	5.62	3	167	100	156	100
60	MSSPSRRSSSPESNIDGGSGSGSAGDERKRKRMLSNRESARRSRARKQQRMEELIAEASRLQAENKRVEAQIGAYTTELTKVDGENAVLRARHGELAGRLQALGGVLEIFQVAGAPVDIPEIPDPLLRPWQSPFAPQLATAGGMP DAFQF									
	1	bZIP transcription factor [Triticum aestivum]	gi|62898531	16274.3	9.67	2	34	0	26	97.306
	2	ABI3-interacting protein 2-2 [Triticum aestivum]	gi|223470527	36138.1	5.10	8	33	0		
	3	Basic region/leucine zipper protein [Triticum aestivum]	gi|165974314	16187.3	9.38	1	30	0	26	97.306
	4	Serine/threonine protein kinase [Triticum aestivum]	gi|14571547	45928.1	8.39	8	28	0		
	5	Amylogenin [Triticum aestivum]	gi|4158230	38841.7	6.19	7	27	0		
	6	Glycosyltransferase 47 [Triticum aestivum]	gi|301072484	46888.9	6.86	4	26	0	17	78.6
	7	AP2-like protein [Triticum aestivum], putative	gi|40644766	19540.8	9.24	5	26	0		
	8	UDP-glucose glucosyltransferase [Triticum aestivum]	gi|86439711	50613.5	5.57	8	26	0		
	9	Glycosyltransferase, HGA-like [Triticum aestivum], putative,expressed	gi|300681530	68832	6.38	9	25	0		
	10	Rp1-like protein [Triticum aestivum]	gi|304325222	138477.8	8.32	8	24	0	15	67.461
61	MECVIGVVGSDFAVVAADTSAVQSILVHKTDEDKIMLLDSHKLMGASGEPGDRVQFTEFIQKNLHLYQFRNNMPLSTAATANFTRGELATALRKNPYSVNIILAGFDKDAGASMYYIDYIATLHKIDKGAFGYGSYFCLSLMDK LYRPDMTVEEAVDLVDKCIKEIRLRLVVAPQNFAIKIVDKDGARDYARREIGGDSPAVTTATATIATTA									
	1	Oxygen-evolving enhancer protein 2, chloroplastic	gi|131394	27252.8	8.84	15	688	100	562	100
	2	20S proteasome beta 4 subunit [Triticum aestivum]	gi|52548238	23313.8	5.57	10	142	100	70	100
	3	Resistance protein CAN_RGA1 [Triticum aestivum]	gi|33302329	101932.3	5.76	7	32	0	23	94.47
	4	Resistance protein LR10 [Triticum aestivum]	gi|33302327	103919.1	5.61	6	32	0	23	94.47
	5	Putative Zn-finger transcription factor [Triticum	gi|60100212	19391.2	8.09	5	30	0		
	6	U2AF small subunit [Triticum aestivum]	gi|68036691	36715.8	8.93	7	28	0		
	7	FIMBRIATA-like protein [Triticum aestivum]	gi|86439727	42631.9	8.55	7	28	0		
	8	Unnamed protein product [Triticum aestivum]	gi|300681540	18039.2	6.47	5	28	0		
	9	Glutathione-S-transferase [Triticum aestivum]	gi|34979592	14383.5	4.81	4	26	0		
	10	Profilin-1	gi|1346803	14972.4	4.91	4	26	0		
	MAPIKLYGMMLSANVTRVTTLLNELGLEFDFVDVDLRTGAHKHPDFLKLNPFGQIPALQDGDEVVFESRAINRYIATKYGASLLPTPSAKLEAWLEVESHHFYPPARTLVYELVIKPMLGAPTDAAEVDKNAADLAKLLDVYE AHLAAGNKYLAGDAFPLADANHMSYLFMLTKSPKADLVASRPHVKAWWEEISARPAWAKTVASIPLPPAV									
	1	Glutathione transferase F5 [Triticum aestivum]	gi|23504745	23422.2	5.78	11	556	100	470	100
	2	S-like RNase [Triticum aestivum]	gi|41400293	25041.9	5.84	5	26	0		
	3	LEA3 protein [Triticum aestivum]	gi|25989707	21837.8	8.83	5	24	0		

62	4	NAC transcription factor 6A [Triticum aestivum]	gi|293631975	1785.9	6.51	2	24	0		
	5	NBS-LRR type R protein [Triticum aestivum]	gi|29892701	18816	6.72	4	23	0		
	6	Mannose-binding lectin [Zea mays]	gi|71370269	13043.2	6.54	4	23	0		
	7	Ribulose bisphosphate carboxylase small chain clone 512	gi|132107	13046.5	5.84	4	23	0		
	8	Xylanase inhibitor TL-XI precursor [Triticum aestivum]	gi|110836641	15632.5	8.38	4	22	0		
	9	60S ribosomal protein L5-2	gi|55976534	34641.7	9.02	7	22	0		
	10	Starch synthase IV [Triticum aestivum]	gi|89214474	102969.4	5.91	10	21	0		
63	MALEAAYHYLQRAVGHGTSTEALLLTVLLLLIIRVAWVRAFTTTTASTKCKQQLPPTPPGKLPIIGHLHLIGSHPHVSFRDLAAKHGRDGLMLVHVGAVPTVVVSTPQAAEAVLRTHDHVFASRPRNPVADIIRYNSTDIAFAPYGDYWRRARKVVNTHPLSVKMVYSKRHDREEEVRLVVAKICELAMAAPGKALDMTELLGGYASDFVCRAVLGESHRKHGRNELFRELTEISASLLGGFNLEDYFPRLATLDVFLRVVCSKAMGVSKRWDNLFNELIAEYEHGKEDNAEDFVHLLLSLKKEYGLSTDNVKAILVNMFEAAIETSFLVLEYSMAELINNRHVMAKVRKEVRESTPKGEKLDLIMEEDLSRMPYLKATIKEAMRIHPPAPFLLPHFSTNDCEVNGYTIPAGTRVIVNAWALARDPSHWERVEEFYPERFLQEGRDAEVDMYGKDTRFVPFGAGRRICAGATFTIATVEVMLANLIYHFDWELPSEMEAIGAKVNMTDQFGMTLRRTERLHLVPKIYK									
	1	P450 [Triticum aestivum]	gi|51572463	59563.8	7.10	7	19	0		
	2	Abscisic acid-induced protein [Triticum aestivum]	gi|32400834	10950.2	11.74	4	18	0		
	3	Small heat shock protein Hsp23.6 [Triticum aestivum]	gi|4138871	23622	5.25	4	17	0		
	4	P450 [Triticum aestivum]	gi|48526685	59236.6	6.50	6	15	0		
	5	MLo1 protein [Triticum aestivum]	gi|14334169	60363.5	9.54	6	15	0		
	6	26s proteasome non-ATPase regulatory subunit [Triticum aestivum]	gi|340842127	34247.4	6.00	4	14	0		
	7	Mlo protein [Triticum aestivum]	gi|46405855	47151.9	9.47	5	14	0		
	8	Annexin [Triticum aestivum]	gi|38606205	35416.1	9.01	5	14	0		
	9	40S ribosomal protein S9 [Triticum aestivum]	gi|32400776	7525	10.14	2	13	0		
	10	Hypothetical protein [Triticum aestivum]	gi|86439744	7050.5	8.61	2	13	0		
64	MASKVELVVEVKSPAEKLWTAMRESTELFPKIFPEQYKSIETMEGDGKSAGTVRLIKYTEGVPMVTFAKEKVEVADDEKKVVSYSVVDGELVSFYKNFRVTVQVTDKGADGAGAVVNWTMDFDKASDEVPEPDVIKETAAKTFHDLDDYLLKN									
	1	Salt tolerant protein [Triticum aestivum]	gi|63021412	17054.6	4.71	9	349	100	279	100
	2	P450 [Triticum aestivum]	gi|51572463	59563.8	7.10	8	27	0		
	3	Puroindoline-like protein [Triticum durum], putative	gi|169788571	16703.3	9.07	6	26	0		
	4	Unknown protein [Triticum aestivum]	gi|374086126	34351.5	6.41	7	25	0		
	5	Os09g0485900 [Oryza sativa Japonica Group]	gi|115479853	21335.4	9.62	5	25	0		
	6	High-molecular-weight glutenin subunit [Triticum spelta]	gi|32328651	15005.7	8.70	4	24	0		
	7	PINB-2v2-2 [Triticum aestivum]	gi|376372849	16850.4	9.29	6	24	0		
	8	ATPase-like zinc transporter [Triticum aestivum], putative	gi|95114390	110389.1	6.58	11	24	0		
	9	Glycosyltransferase [Triticum aestivum]	gi|56409844	65081	5.95	8	23	0		
	10	Serine/threonine kinase [Triticum aestivum], putative	gi|86439758	29422.2	7.74	5	23	0		
65	MACTQENAMATDESTADHRRSRPSSHDMDLSGDDHVPKARKPYTITKQREKWTEEEHKRFLEALQLHGRAWRRIQEHIGTKTAVQIRSHAQKFFSKVTRESSGSSSGSGAAAATATAAIQIPPPRPKRKPTHPYPRKADDGAAAGGKHAPGLTHLERPPVRMGEQEEGSPTSVLTASRVEASGGRFSNNSSGSRSPVPSAAGSLYGSSVDRGDGCLSPNTKTSEITANGNVKEGSCTGSATSVLKLFGKKVVVNDSFQKPNTSTGNPQNGADVGTEASDDTTTQGSRNLPSGGATEGSSWNPWPSNMQQFVYFVPQPDGFATQSAVPWFGTLPGAMFYQQAVAPNQHQRHRSETADHKFMQREGSWTGSNTGPGSAAHNSDAADSRGRGNISESDKTLVPRLTKCESSVSISLQRGFMPYKRCAAESESLRSEAPREETDGELTRLCL									
	1	MYB-related protein [Triticum aestivum]	gi|359952782	48051.4	8.93	11	54	97.893		
	2	Glycosyltransferase [Triticum aestivum]	gi|56409866	48626.6	6.28	9	40	52.833	3	0
	3	TaGT61_1 [Triticum aestivum]	gi|334353407	55806.6	8.45	9	36	0	3	0
	4	Pm3 [Triticum aestivum]	gi|296280016	158971	6.07	17	36	0		
	5	Pm3 [Triticum aestivum]	gi|296280026	159148.2	6.30	17	34	0		
	6	Powdery mildew resistance protein PM3 variant	gi|225580375	157346.2	6.26	16	33	0		
	7	Pm3 [Triticum aestivum]	gi|296280014	159019	6.14	16	31	0		
	8	Powdery mildew resistance protein PM3 variant [Triticum aestivum]	gi|225580385	159106.2	6.35	16	31	0		
	9	Mla-like protein [Triticum aestivum]	gi|24571201	60507.5	8.50	10	30	0		
	10	Ribosomal protein L16 [Triticum aestivum]	gi|254998340	10715.8	10.64	4	30	0		
	MAPIGVGSTLPDGQLGWFDENDQLQQVSIHSLATGKKVILFGVPGAFTPTCSNQHVPGFITQAEELKAKGVDEILLVSVNDPFVMKAWAKTYPENKHVKFLADGAAAYTKALGLELDLTEKGLGLRSRRFALLADDLKVTVANIEEGGQFTISGAEEIMKAL									
	1	Thioredoxin-dependent peroxidase [Leymus chinensis]	gi|256708473	17388.1	5.17	11	362	100	266	100

66	2	Predicted protein [Hordeum vulgare subsp. vulgare]	gi|326501648	17312.1	5.17	6	202	100	164	100
	3	Peroxiredoxin-2C-like [Brachypodium distachyon], predicted	gi|357135911	17366.1	5.35	6	202	100	164	100
	4	Peroxiredoxin 5 cell rescue protein, partial [Lolium perenne]	gi|375152308	11445.1	5.13	5	169	100	134	100
	5	MADS box transcription factor [Salvia coccinea]	gi|343788119	24834.8	9.59	2	72	93.172	65	99.891
	6	Deficiens [Salvia coccinea]	gi|42795291	26819.9	9.50	2	72	92.851	65	99.891
	7	Os11g0238700 [Oryza sativa Japonica Group]	gi|297611534	106593.5	8.76	19	61	23.394		
	8	Unknown [Picea sitchensis]	gi|224286654	71308.3	5.71	15	60	0		
	9	(-)-alpha/beta-pinene synthase [Picea sitchensis]	gi|325930012	71324.3	5.71	15	59	0		
	10	Predicted protein [Populus trichocarpa]	gi|224125614	44652.8	5.36	7	52	0	27	0
67		MSDTDEHHFESKADSGASKTYPQQAGAIRKGGHIVIKARPCKVVEVSTSKTGKHGHAKCHFVAIDIFNGKKLEDIVPSSHNCDVPHVDRQDYQLIDITDDGYVSLLTESGNTKDDLKLPTDDVLLGQIKTGFADGKDLILSVMS AMGEEQICAVKEIGGGK								
	1	Eukaryotic translation initiation factor 5A1 [Triticum aestivum]	gi|74048999	17351.7	5.70	11	283	100	188	100
	2	Eukaryotic translation initiation factor 5A3 [Triticum aestivum]	gi|74049040	17370.7	5.76	11	281	100	188	100
	3	Kinase R-like protein [Triticum aestivum]	gi|17981566	18856.8	8.73	6	34	0		
	4	Wheat win6-b defense protein [Triticum aestivum]	gi|78096539	23013.3	9.37	6	30	0		
	5	Annexin [Triticum aestivum]	gi|38606205	35416.1	9.01	7	27	0		
	6	Ribulose-1,5-bisphosphate carboxylase/oxygenase large subunit [Triticum aestivum]	gi|339742637	21900	7.57	5	26	0		
	7	Cystatin [Triticum aestivum]	gi|194338899	26645.9	5.95	5	23	0		
	8	Basic region leucine zipper protein [Triticum aestivum]	gi|172052528	37902.1	8.94	6	23	0		
	9	Putative protein [Triticum aestivum]	gi|283099379	57999.7	5.26	7	22	0		
	10	Avenin-like b11; Flags: Precursor	gi|338817631	32818	8.51	5	22	0		
68		MSDTDEHHFESKADSGASKTYPQQAGAIRKGGHIVIKARPCKVVEVSTSKTGKHGHAKCHFVAIDIFNGKKLEDIVPSSHNCDVPHVDRQDYQLIDITDDGYVSLLTESGNTKDDLKLPTDDVLLGQIKTGFADGKDLILSVMS AMGEEQICAVKEIGGGK								
	1	Eukaryotic translation initiation factor 5A1 [Triticum aestivum]	gi|74048999	17351.7	5.70	10	230	100	148	100
	2	Eukaryotic translation initiation factor 5A3 [Triticum aestivum]	gi|74049040	17370.7	5.76	10	228	100	148	100
	3	Zeta-carotene desaturase [Triticum aestivum]	gi|336185123	62491	7.09	9	33	0	7	0
	4	Cytochrome b6-f complex iron-sulfur subunit, chloroplastic	gi|68566191	23711	8.47	6	30	0		
	5	Pathogenesis-related protein 1 [Triticum aestivum]	gi|83031480	11185.2	6.88	4	29	0		
	6	Annexin [Triticum aestivum]	gi|38606205	35416.1	9.01	7	27	0		
	7	Putative protein [Triticum aestivum]	gi|283099379	57999.7	5.26	8	27	0		
	8	Zeta-carotene desaturase [Triticum aestivum]	gi|205371883	62501	6.81	8	26	0	6	0
	9	Stress responsive protein [Triticum aestivum]	gi|66277464	22159.6	7.64	5	26	0		
	10	Ferredoxin-NADP(H) oxidoreductase [Triticum aestivum]	gi|20302471	38781.6	8.29	7	25	0		
69		MASTALSTASNPTQLCRTRASSLCKPVKGLGFGRERIPRNITCMAGSISADRVPDMSKRELMNLLLLGAISLPTFGMLVPYGSFLVPAGSGSNAGGVAAKDKLGNDILVEDWLKTHGPNDRTLAQGLKGDPTYLVVESDKTLAT YGINAVCTHLGCVVPWNAAENKFLCPCHGSQYNNQGKVVRGPAPLSLALVHADVDDGKVVFVPWVETDFRTGDNPWWK								
	1	Cytochrome b6-f complex iron-sulfur subunit, chloroplastic	gi|68566191	23711	8.47	10	699	100	629	100
	2	Zeta-carotene desaturase [Triticum aestivum]	gi|336185123	62491	7.09	10	33	0		
	3	Glycosyltransferase [Triticum aestivum]	gi|56409846	52982.5	6.14	3	33	0	26	97.304
	4	WALI7 [Triticum aestivum]	gi|257135793	28053.7	6.99	6	30	0		
	5	cp31BHv [Triticum aestivum]	gi|226533870	18993.4	4.85	5	29	0		
	6	Zeta-carotene desaturase [Triticum aestivum]	gi|205371883	62501	6.81	9	27	0		
	7	Glycosyl transferase [Triticum aestivum], putative	gi|342186837	65104.1	6.34	2	26	0	26	97.304
	8	Starch synthase IIa-1 [Triticum aestivum]	gi|7529653	86891.4	6.05	9	24	0		
	9	NBS-LRR type R protein [Triticum aestivum]	gi|29892701	18816	6.72	4	24	0		
	10	Ribulose bisphosphate carboxylase	gi|132107	13046.5	5.84	4	24	0		
		KAEEGPVDYEKEIKHHKHLEQIGKLGTVAAGAYALHEKHEAKKDPEHAHKHKIEEEIAAAAAIGAGGFAFHEHHEKKDAKKEEKKAEGGHHHLF								
	1	ASR1 protein [Solanum peruvianum]	gi|321155395	10452.3	6.55	2	85	99.666	73	99.983
	2	ASR1 protein [Solanum peruvianum]	gi|321155389	12698.3	6.50	2	84	99.559	73	99.983
	3	CNL-B17 [Phaseolus vulgaris]	gi|270342130	126058.9	6.32	19	55	0		

	4	Predicted protein [Populus trichocarpa]	gi|224079477	11660.8	9.05	7	49	0		
70	5	Transcription initiation factor IIB-like [Brachypodium distachyon], predicted	gi|357155150	32983.5	7.08	9	47	0		
	6	Conserved hypothetical protein [Ricinus communis]	gi|255539160	31914.5	7.56	10	45	0		
	7	Predicted protein [Populus trichocarpa]	gi|224069738	32304.7	8.68	9	45	0		
	8	Hypothetical protein [Oryza sativa Japonica Group]	gi|12313669	21674.2	11.55	6	45	0	26	0
	9	GBSSI [Iochroma edule]	gi|83755379	27986.1	7.59	8	45	0		
	10	Contains similarity to gb|AJ009695 wall-associated kinase 4 from Arabidopsis thaliana and contains a protein kinase PF|000	gi|6587818	77599.2	7.93	15	45	0		
		MATAASLSALSSAAAAGKRFVLSSPSLSFASRRLAAPAHLRAAFGPGARRVAAAAATASPVVATIAVGDKLPDATLSYFDPADGELKTVTVGELTAGKKAVLFAVPGAFTPTCSQKHLPGFVAAAGDLRAKGVDTVACVSVNDA FVMKAWKESLGLGDDAGVMMLSDGNLELTRALGVEMDLSDKPMGLGVRSRRYALLADDGVVKVLNLEEGGAFTTSSAEEMLKVL								
	1	Hypothetical protein OsI_06200 [Oryza sativa Indica	gi|125538439	23135.1	6.15	8	193	100	146	100
	2	Peroxiredoxin-2E-2, chloroplastic-like [Brachypodium distachyon], predicted	gi|357139104	23212.1	6.74	6	176	100	146	100
	3	Hypothetical protein OsJ_05717 [Oryza sativa Japonica	gi|125581128	23146.9	8.49	5	169	100	146	100
71	4	Hypothetical protein OsJ_22032 [Oryza sativa Japonica	gi|125597912	23672.6	9.32	5	132	100	110	100
	5	Mitochondrial peroxiredoxin-5 precursor [Oryza sativa	gi|149391349	2635.4	4.49	1	120	100	110	100
	6	Predicted protein [Hordeum vulgare subsp. vulgare]	gi|326488016	23312.2	5.92	8	82	99.268	36	24.949
	7	Shikimate kinase, chloroplastic-like [Brachypodium distachyon], predicted	gi|357138119	32845.1	9.30	11	61	23.394		
	8	Hypothetical protein SELMODRAFT_449084 [Selaginella moellendorffii]	gi|302823273	44556.8	6.54	12	58	0		
	9	Predicted protein [Populus trichocarpa]	gi|224099459	13896	4.83	6	56	0		
	10	Predicted protein [Hordeum vulgare subsp. vulgare]	gi|326533332	32563.9	9.10	10	52	0		
		MASTDSWTHEIESPVAAARLFRAGVMDWHTLAPKLAPHIVASAHPVEGEGGIGSVRQFNFTSAMPFSLMKERLEFIDADKCECKSTLIEGGGIGTAIETTTSHIKVEPAANGGSVVKVESTYKLLPGVEVNDEITKAKESVTAIF KAAEAYLVANPDAYN								
	1	Pathogenesis related protein 10 [Triticum aestivum]	gi|196051131	17052.6	5.19	8	250	100	188	100
	2	Pathogenesis-related protein [Triticum aestivum]	gi|162414848	17022.6	5.19	7	238	100	188	100
	3	Peroxidase [Triticum aestivum]	gi|21717531	32405.2	8.89	7	33	0		
72	4	Unnamed protein product [Triticum aestivum]	gi|300681470	24476.1	6.77	7	31	0		
	5	P450 [Triticum aestivum]	gi|51572463	59563.8	7.10	9	30	0		
	6	Cytochrome P450 [Triticum aestivum]	gi|26655528	59519.8	6.75	8	26	0		
	7	Putative major allergen Phl p 5 [Triticum aestivum]	gi|40644796	23934.2	5.06	5	26	0		
	9	Conserved hypothetical protein, expressed [Triticum aestivum]	gi|300681478	39218.4	5.68	6	24	0		
	10	Monomeric alpha-amylase inhibitor [Triticum monococcum]	gi|134034506	13088.3	7.40	5	24	0		
		MALETCLRGWALYAPQAGIRERLSSGSYAPSRPRTAAPAVVSPSPYKSALVAARRPSRFVCKCKNVVDEVIVADEKNWDNMVIACESPVLVEFWAPWCGPCRMIAPVIDELAKDYVGKIKCCKVNTDDCPNIASTYGIRSIPTVL MFKDGEKKESVIGAVPKTTLCTIIDKYIGS								
	1	Thioredoxin M-type, chloroplastic; Precursor	gi|11135474	19119.7	8.67	7	208	100	172	100
	2	Ribulose bisphosphate carboxylase small chain clone 512	gi|132107	13046.5	5.84	5	32	0		
	3	MYB-related protein [Triticum aestivum]	gi|359952782	48051.4	8.93	8	32	0		
	4	Serine/threonine protein kinase Stpk-D [Triticum	gi|333385001	44526.8	7.63	7	28	0		
73	5	Unknown protein [Triticum aestivum]	gi|374086126	34351.5	6.41	7	28	0		
	6	NBS-LRR type RGA [Triticum aestivum]	gi|74125019	9104.7	6.75	4	27	0		
	7	S-like RNase [Triticum aestivum]	gi|41400293	25041.9	5.84	5	26	0		
	8	Chain A, Crystal Structure Of A Mutant Beta-Glucosidase In Wheat Complexed With Dimboa-Glc	gi|326327792	63988.7	5.39	8	25	0		
	9	Ribulose-1,5-bisphosphate carboxylase/oxygenase small subunit [Triticum aestivum]	gi|11990893	19500.8	9.06	5	24	0		
	10	Ribulose-1,5-bisphosphate carboxylase/oxygenase small subunit [Triticum aestivum]	gi|11990897	19447.8	8.80	5	24	0		
		AYLPPLSTEALLKQVDYLIRSKWVPCLEFSKVGFIFREHNASPGYYDGRYWTMWKLPMFGCTDATQVINEVEEVKKEYPDAYVRIIGFDNMRQVQCVSFIAFKPPGCEESGKA								
	1	Ribulose bisphosphate carboxylase small chain clone 512 (Fragment) [Triticum aestivum]	RBS3_WHEAT	13046.5	5.84	6	40	95.994		
	2	Bowman-Birk type proteinase inhibitor II-4 (Fragment) [Triticum aestivum]	IBB2_WHEAT	5948.5	8.45	5	20	0		
	3	Phenylalanine ammonia-lyase [Triticum aestivum]	PALY_WHEAT	75889.5	6.10	9	15	0		
	4	Alpha-2-purothionin [Triticum aestivum]	THN2_WHEAT	14547.8	5.13	4	14	0		
74	5	Phosphoribulokinase, chloroplastic [Triticum aestivum]	KPPR_WHEAT	45112.9	5.72	5	13	0		
	6	Metallothionein-like protein 1 [Triticum aestivum]	MT1_WHEAT	7371	4.44	2	12	0		

	7	Endogenous alpha-amylase/subtilisin inhibitor [Triticum aestivum]	IAAS_WHEAT	19620.8	6.77	3	12	0		
	8	Cytochrome b559 subunit alpha [Triticum aestivum]	PSBE_WHEAT	9438.7	4.64	2	12	0		
	9	Ribulose bisphosphate carboxylase small chain PW9, chloroplastic [Triticum aestivum]	RBS2_WHEAT	19441.7	8.52	3	12	0		
	10	DNA-directed RNA polymerase subunit beta" [Triticum aestivum]	RPOC2_WHEAT	169939	6.50	10	11	0		
75	MAPAVMASSATSVAPFQGLKSTAGLPVSRRSNGASLGSVSNGGRIRCMQVWPIEGIKKFETLSYLPPLSTEALLKQVDYLIRSKWVPCLEFSKVGFIFREHNASPGYYDGRYWTMWKLPMFGCTDATQVINEVEEVKKEYPDAYVRIIGFDNMRQVQCVSFIAFKPPGCEESGKA									
	1	Ribulose bisphosphate carboxylase small chain clone 512	gi|132107	13046.5	5.84	10	196	100	107	100
	2	Ribulose-1,5-bisphosphate carboxylase/oxygenase small subunit [Triticum aestivum]	gi|11990897	19447.8	8.80	11	188	100	107	100
	3	Ribulose-1,5-bisphosphate carboxylase/oxygenase small subunit [Triticum aestivum]	gi|11990893	19500.8	9.06	11	187	100	107	100
	4	Ribulose-1,5-bisphosphate carboxylase/oxygenase small subunit [Aegilops longissima]	gi|4038673	18533.4	8.83	11	127	100	42	99.898
	5	Ribulose-1,5-bisphosphate carboxylase/oxygenase small subunit [Triticum aestivum]	gi|11990901	19448.8	8.81	9	102	100	42	99.898
	6	Ribulose bisphosphate carboxylase small chain PW9, chloroplastic	gi|132099	19441.7	8.52	8	91	100	42	99.898
	7	Ribulosebisphosphate carboxylase [Triticum aestivum]	gi|755804	18119.9	8.95	7	91	100	42	99.898
	8	Ribulose-bisphosphate carboxylase (EC 4.1.1.39) small chain precursor (clone 234) - wheat (fragment)	gi|82619	15344.9	8.95	7	90	100	42	99.898
	9	Ribulose-1,5-bisphosphate carboxylase/oxygenase small subunit [Triticum aestivum]	gi|11990895	19420.8	8.99	8	90	100	42	99.898
	10	Ribulose-1,5-bisphosphate carboxylase/oxygenase small ubunit [Triticum aestivum]	gi|11990899	19547.8	8.99	8	90	100	42	99.898
76	MSWQTYVDDHLCCEIDGQHLTSAAILGHDGSVWAQSPNFPQFKPEEIAGIIKDFEEPGHLAPTGLFLGGTKYMVIQGEPGVVIRGKKGTGGITIKKTGMALILGIYDEPMTPGQCNLVVERLGDYLVEQGF									
	1	Profilin [Triticum aestivum]	gi|300807845	14182.1	4.89	5	129	100	91	100
	2	Profilin [Triticum aestivum]	gi|190684061	14153.1	4.93	5	71	99.963	33	99.474
	3	Profilin [Triticum aestivum]	gi|207366248	14154.1	5.04	4	62	99.666	33	99.474
	4	Profilin-1	gi|1346803	14972.4	4.91	4	61	99.589	33	99.474
	5	Profilin-3	gi|1346806	15201.5	5.78	4	61	99.57	33	99.474
	6	Profilin-2	gi|1346804	15383.6	5.73	4	60	99.56	33	99.474
	7	Ribulose-bisphosphate carboxylase (EC 4.1.1.39) small chain precursor (clone 234) - wheat (fragment)	gi|82619	15344.9	8.95	5	31	0		
	9	Translation initiation factor eIF5 [Triticum aestivum]	gi|126513560	49000.7	5.56	8	26	0		
	10	Ribulose-1,5-bisphosphate carboxylase/oxygenase small subunit [Triticum aestivum]	gi|11990893	19500.8	9.06	5	25	0		
77	MALLLNVSLSSKLPPFPILKPHLSFPKPHLNSSLFHNTNSATPQGNGINFDHLHLSVTRGSFVALLSASLFFVDPALAFKGGGPYGAEVTRGQDLTGKDFSGKSLIKQDFKTSILRQANFKGANLLGASFFDADLTGADLSDADLRGADFSLANVTKANLSNANLEGALATGNTSFRGSIITGADFTDVPLREDQREYLCKVADGVNPTT									
	GNATRETLLCN									
	1	Predicted protein [Hordeum vulgare subsp. vulgare]	gi|326506328	19578.9	10.17	9	628	100	566	100
	2	Predicted protein [Hordeum vulgare subsp. vulgare]	gi|326523645	20905.6	6.06	8	568	100	520	100
	3	Hypothetical protein VITISV_013914 [Vitis vinifera]	gi|147774410	23432.4	6.41	1	255	100	255	100
	4	Thylakoid lumenal 15 kDa protein 1, chloroplastic [Vitis vinifera], predicted	gi|225449424	23417	6.50	1	255	100	255	100
	5	Thylakoid lumenal 15 kDa protein 1, chloroplastic-like [Brachypodium distachyon], predicted	gi|357133836	20732.5	6.71	6	228	100	197	100
	6	Os01g0144100 [Oryza sativa Japonica Group]	gi|115434488	20561.5	6.71	4	146	100	130	100
	7	Hypothetical protein OsJ_00337 [Oryza sativa Japonica	gi|222617721	51873.1	9.49	4	71	91.6	62	99.71
	8	Hypothetical protein OsI_00358 [Oryza sativa Indica Group]	gi|218187501	19899.2	6.71	2	69	87.576	62	99.71
	9	CNL-B17 [Phaseolus vulgaris]	gi|270342130	126058.9	6.32	19	56	0		
	10	Maturase K [Araucaria hunsteinii]	gi|264684339	24374.9	9.65	9	50	0		
78	MAGRSNIPANNSALIAIIADEDTVTGFLMAGVGNVDLRKKTNYLLVDNKTTVKQIEDAFKEFTAREDIAIVLISQYIANMIRFLVDSYNKPIPAILEIPSKDHPYDPASDSVLSRVKYLFSADSVASDRR									
	1	Vacuolar ATPase subunit F [Triticum aestivum]	gi|94537548	14370.5	5.33	7	90	100	34	99.285
	2	Powdery mildew resistance protein PM3C [Triticum aestivum]	gi|82492377	159009.1	6.18	14	34	0	9	0
	3	MYB-related protein [Triticum aestivum]	gi|359952782	48051.4	8.93	7	25	0		
	4	Powdery mildew resistance protein PM3 variant [Triticum aestivum]	gi|225580375	157346.2	6.26	14	25	0		
	5	Pm3 [Triticum aestivum]	gi|296280016	158971	6.07	14	25	0		
	6	LEA D-11 dehydrin [Triticum aestivum]	gi|21624242	12820.1	7.21	4	24	0		
	7	Annexin [Triticum aestivum]	gi|38606205	35416.1	9.01	7	24	0		
	8	S-like RNase [Triticum aestivum]	gi|41400293	25041.9	5.84	4	24	0	5	0
	9	Powdery mildew resistance protein PM3 variant [Triticum aestivum]	gi|225580385	159106.2	6.35	14	24	0		

	10	Glycosyltransferase [Triticum aestivum]	gi|56409844	65081	5.95	8	24	0		
79	AYLPPLSTEALLKQVDYLIRSKWVPCLEFSKVGFIFREHNASPGYYDGRYWTMWKLPMFGCTDATQVINEVEEVKKEYPDAYVRIIGFDNMRQVQCVSFIAFKPPGCEESGKA									
	1	Ribulose-1,5-bisphosphate carboxylase/oxygenase small subunit [Triticum aestivum]	gi|11990901	19448.8	8.81	12	463	100	364	100
	2	Ribulose bisphosphate carboxylase small chain PWS4.3, chloroplastic	gi|132087	19404.8	8.99	10	437	100	364	100
	3	Ribulose bisphosphate carboxylase small chain PW9, chloroplastic	gi|132099	19441.7	8.52	12	414	100	317	100
	4	Ribulose-1,5-bisphosphate carboxylase/oxygenase small subunit [Triticum aestivum]	gi|11990899	19547.8	8.99	11	401	100	317	100
	5	Ribulose-1,5-bisphosphate carboxylase/oxygenase small subunit [Triticum aestivum]	gi|11990895	19420.8	8.99	11	366	100	281	100
	6	Ribulose-1,5-bisphosphate carboxylase/oxygenase small subunit [Aegilops longissima]	gi|4038673	18533.4	8.83	11	339	100	248	100
	7	Ribulose-1,5-bisphosphate carboxylase/oxygenase small subunit [Triticum aestivum]	gi|4038719	18518.4	8.83	11	336	100	248	100
	8	Ribulose-1,5-bisphosphate carboxylase/oxygenase small subunit [Aegilops searsii]	gi|4038679	18588.4	9.01	11	336	100	248	100
	9	Ribulose-1,5-bisphosphate carboxylase/oxygenase small subunit [Triticum aestivum]	gi|11990893	19500.8	9.06	12	216	100	119	100
	10	Ribulose bisphosphate carboxylase small chain clone 512	gi|132107	13046.5	5.84	9	199	100	119	100
80	MVFVKNQKTRAYSKRFQVKFRRRRQGKTDYRARLRLTNQDKNKYNTPKYRFVVRFTNKDVTAQIVYATIAGDIVMAAAYSHELPRYGLEVGLTNYAAAYCTGLLLARRVLKCRDLDQEYEGNVEATGEDFSVEPADERRPF RALLDVGLIRTTTGNRVFGALKGALDGGLDIPHSDKRFAGFKKDEKQLDAEIHRKYIYGGHVADYMKSLADEEPEKYQSHFSEYIKKGIEIDDMEALYKKVHAAIRADPTVAKSTKEPPKTHKRYNPKKLTYEQRKASLVERL NALNSSAGADVDEDDDE									
	1	Glycosyltransferase [Triticum aestivum]	gi|56409844	65081	5.95	9	29	0	3	0
	2	DIP2 protein [Triticum aestivum], putative	gi|40644804	28099.3	11.37	6	26	0		
	3	Thylakoid-bound ascorbate peroxidase [Triticum aestivum]	gi|25992555	41301.8	5.48	6	25	0	9	0
	4	Annexin [Triticum aestivum]	gi|38606205	35416.1	9.01	7	24	0		
	5	60S ribosomal protein L5 [Triticum aestivum]	gi|360042516	34441.8	9.29	3	24	0	15	41.284
	6	Non-tir-NBS-LRR resistance gene analog, partial [Triticum aestivum], putative	gi|374683017	19459.2	7.77	5	22	0		
	7	Ent-kaurene synthase like 1 [Triticum aestivum]	gi|363987136	94528.2	5.60	6	22	0	13	2.107
	8	Glycosyltransferase, HGA-like [Triticum aestivum], putative,expressed	gi|300681530	68832	6.38	9	22	0		
	9	Zeta-carotene desaturase [Triticum aestivum]	gi|336185123	62491	7.09	9	21	0		
	10	Unknown protein [Triticum aestivum]	gi|374086126	34351.5	6.41	6	21	0		
