# Supplementary figures and images for: A Quantitative Proteomics View on the Function of Qfhb1, a Major QTL for Fusarium Head Blight Resistance in Wheat
Source: Pathogens. 2018 Jun 22;7(3):58. doi: 10.3390/pathogens7030058 (PMC6161305; doi:10.3390/pathogens7030058)

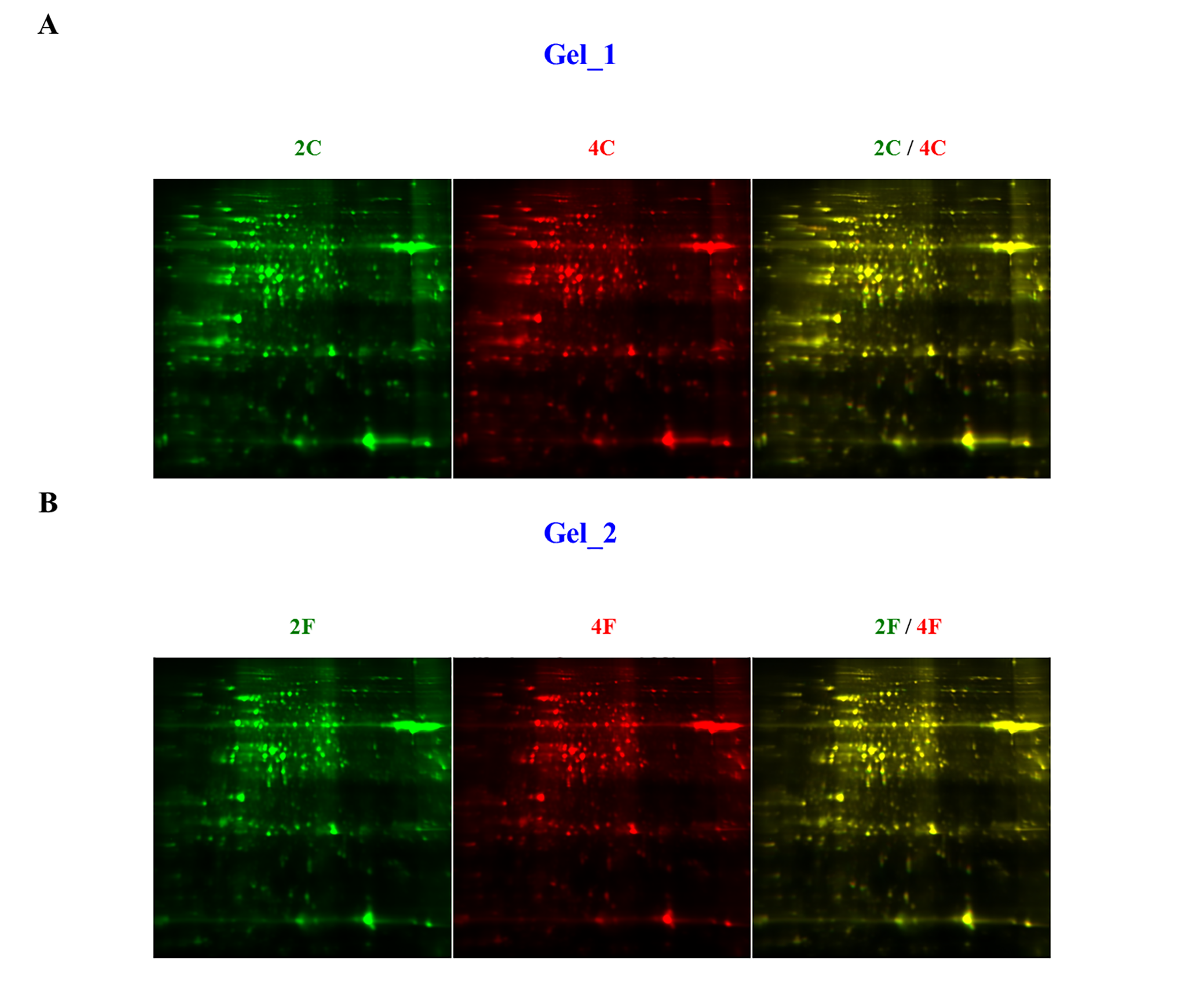

Supplement: Supplementary file 1 [file pathogens-07-00058-s001.zip › Fig S1.tif]

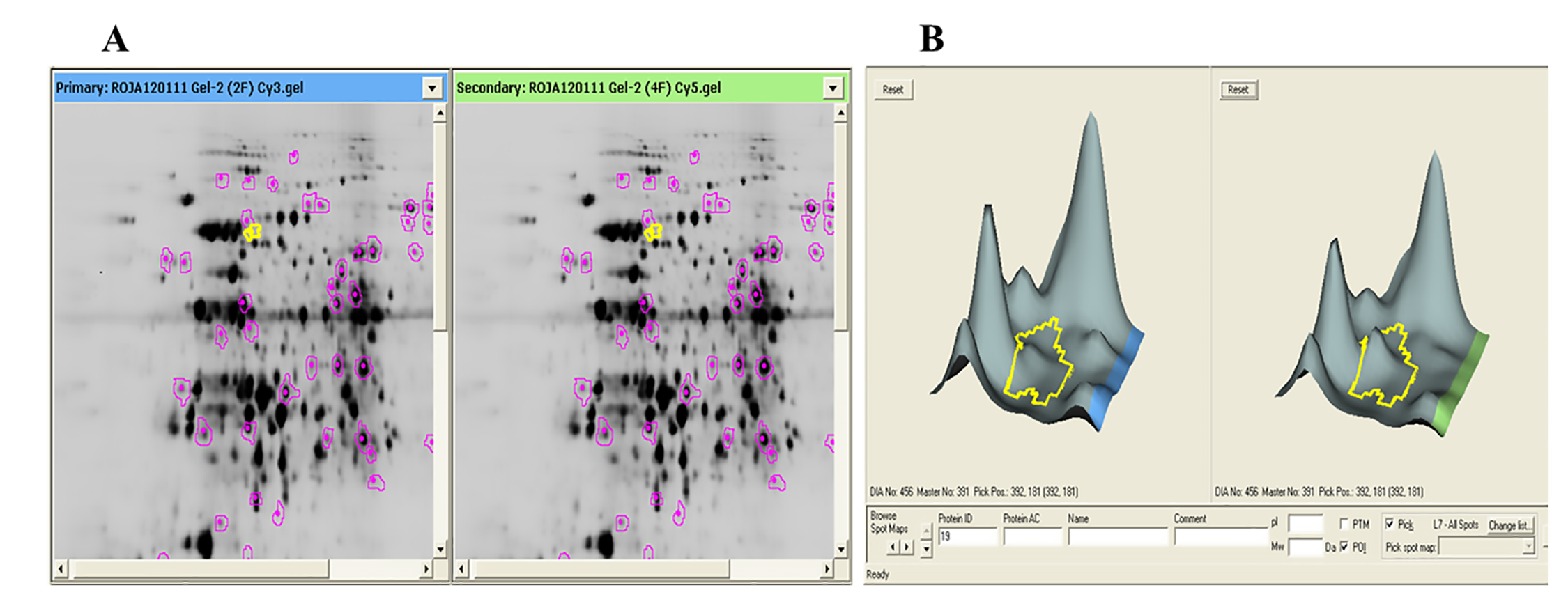

Supplement: Supplementary file 1 [file pathogens-07-00058-s001.zip › Fig S2.tif]

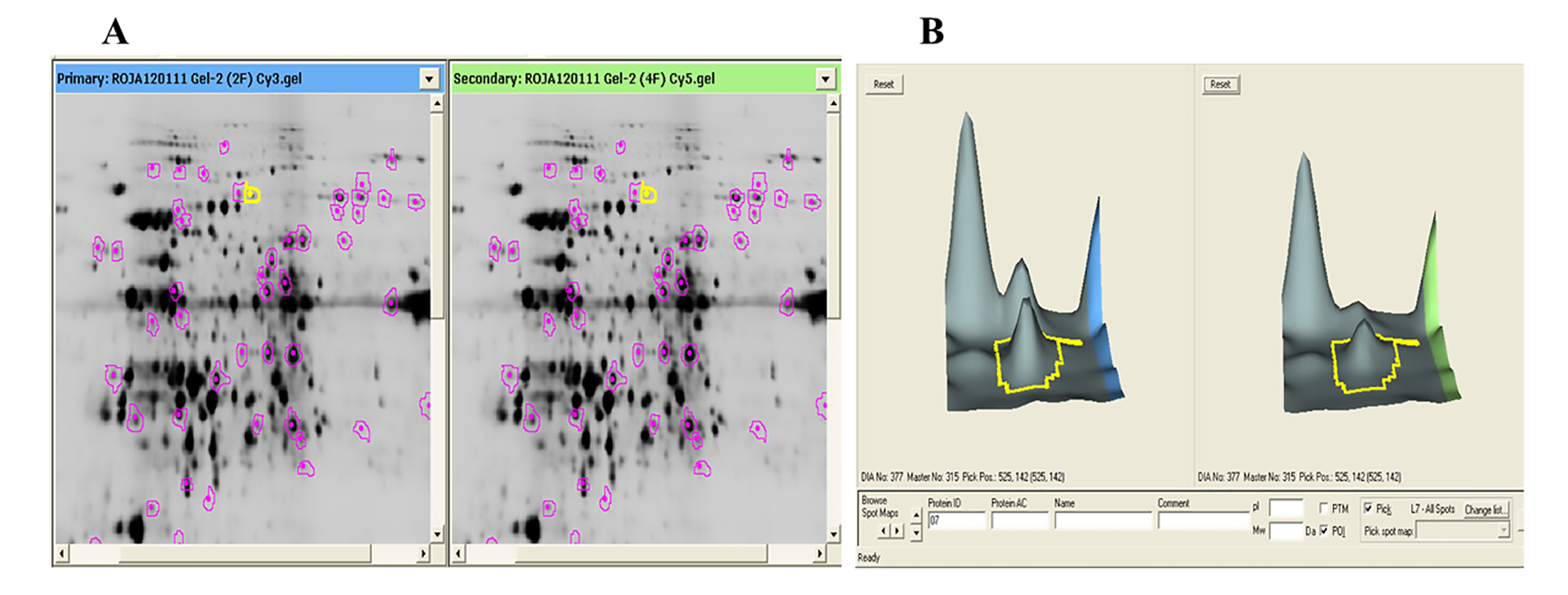

Supplement: Supplementary file 1 [file pathogens-07-00058-s001.zip › Fig S3.tif]

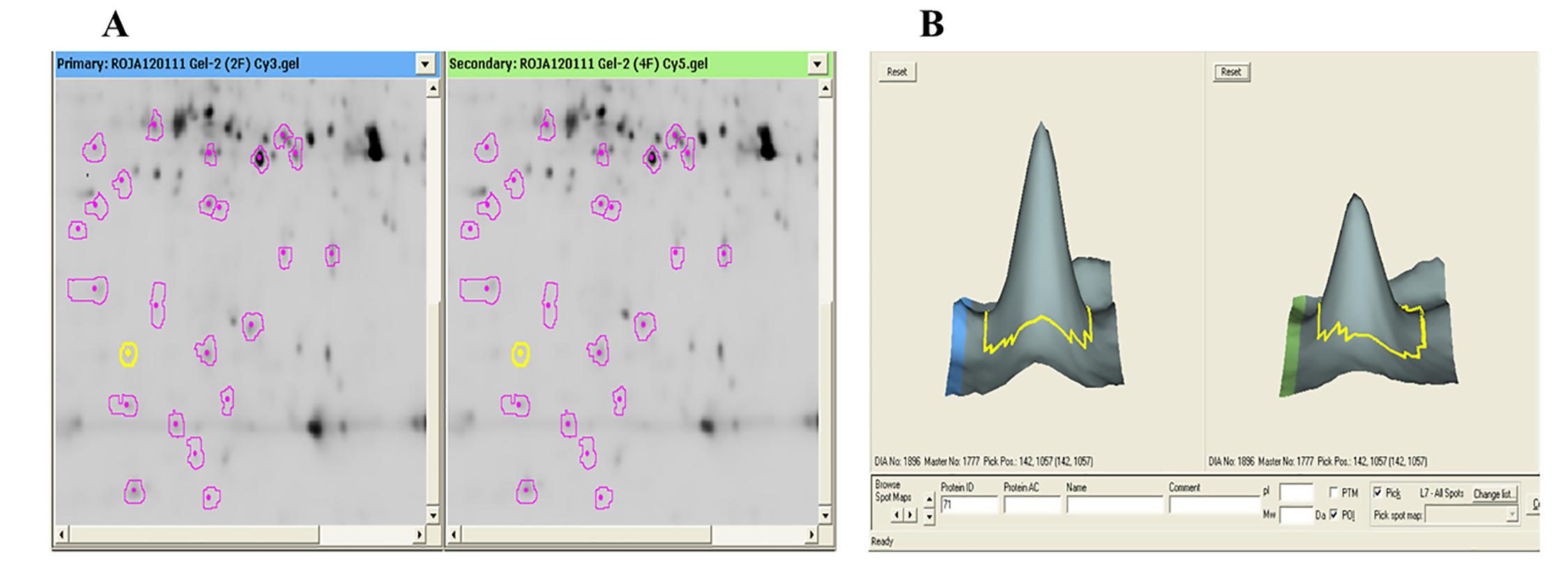

Supplement: Supplementary file 1 [file pathogens-07-00058-s001.zip › Fig S4.tif]

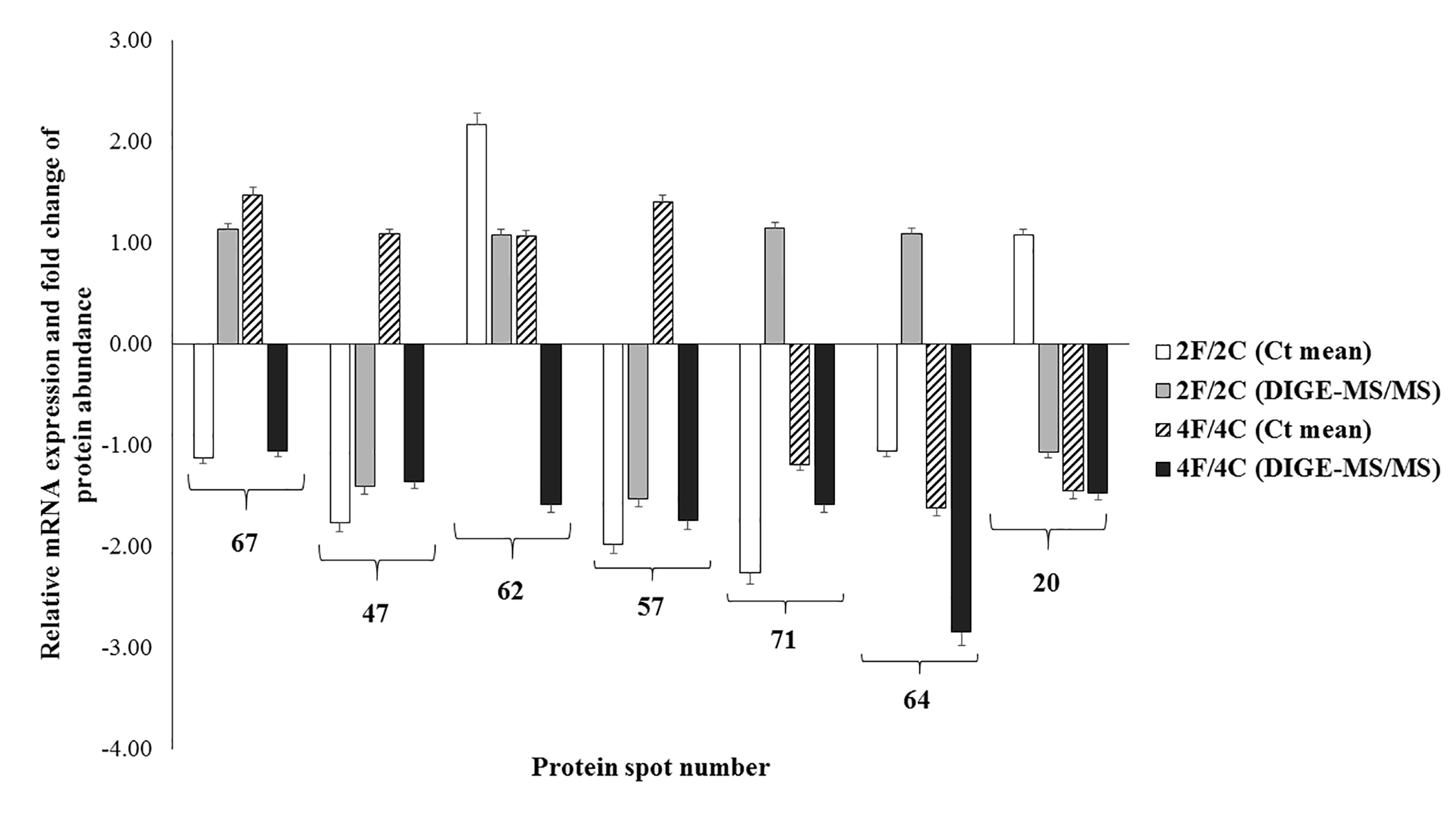

Supplement: Supplementary file 1 [file pathogens-07-00058-s001.zip › Fig S5.tif]

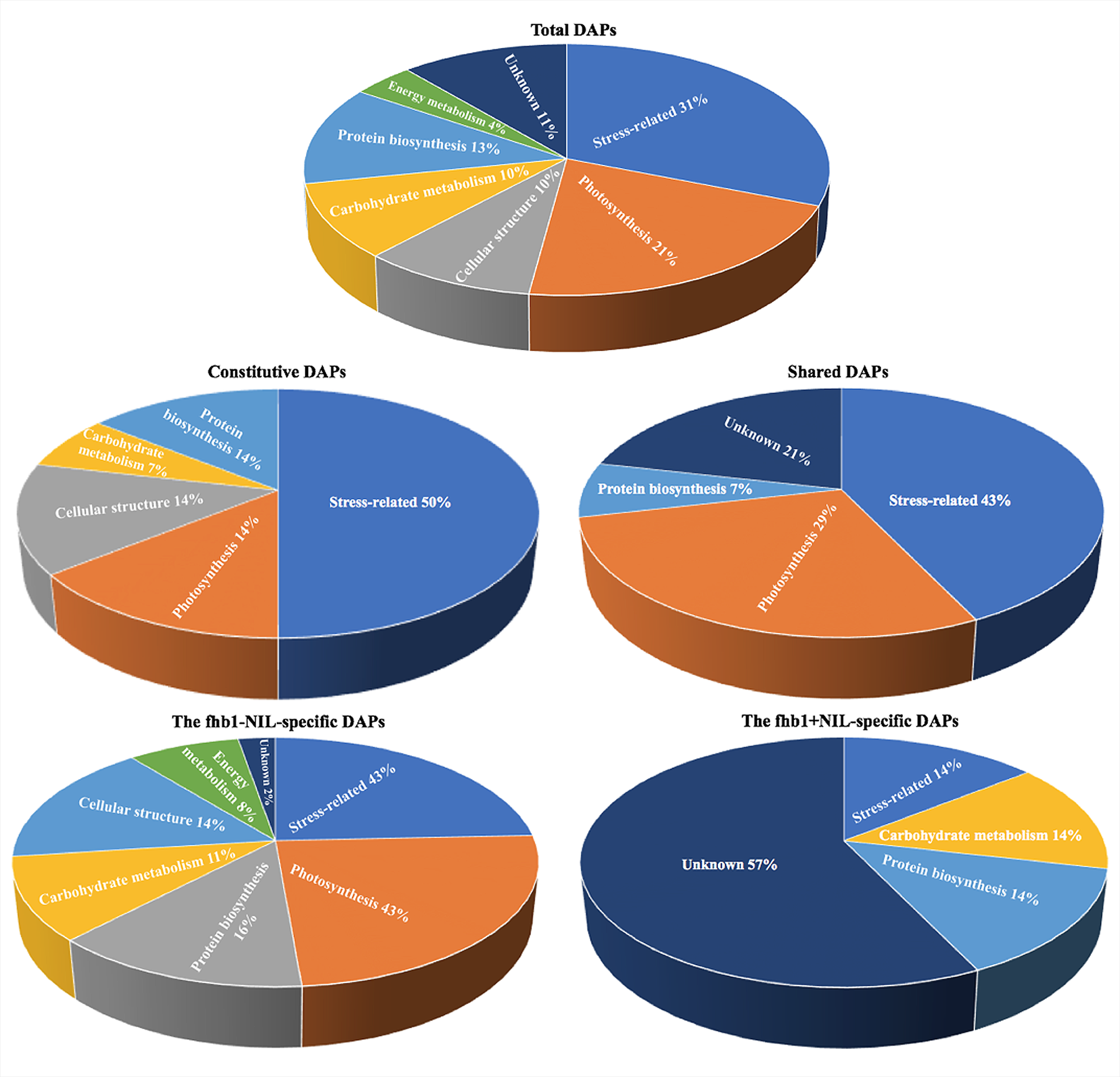

Supplement: Supplementary file 1 [file pathogens-07-00058-s001.zip › Fig S6.tif]

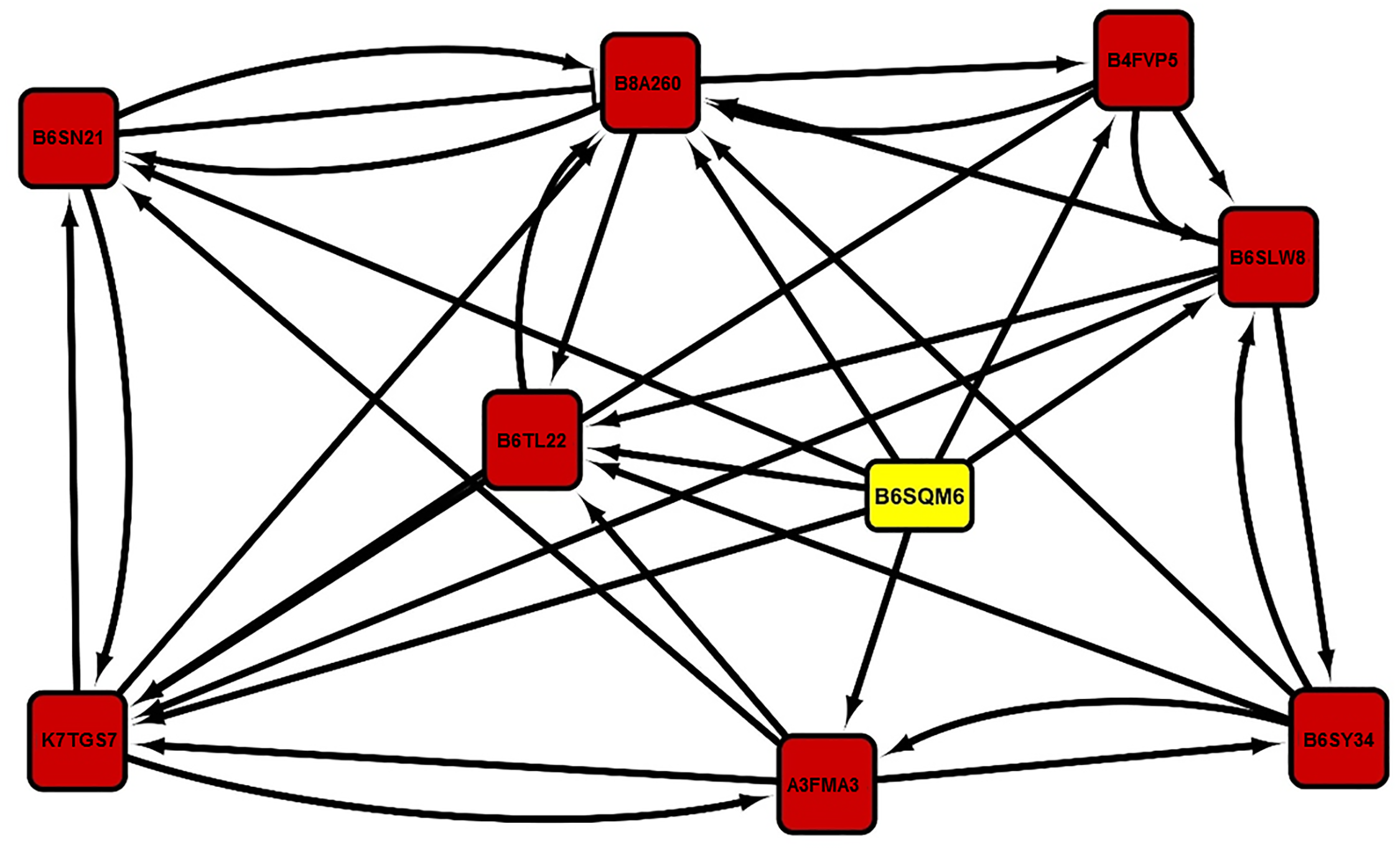

Supplement: Supplementary file 1 [file pathogens-07-00058-s001.zip › Fig S7.tif]

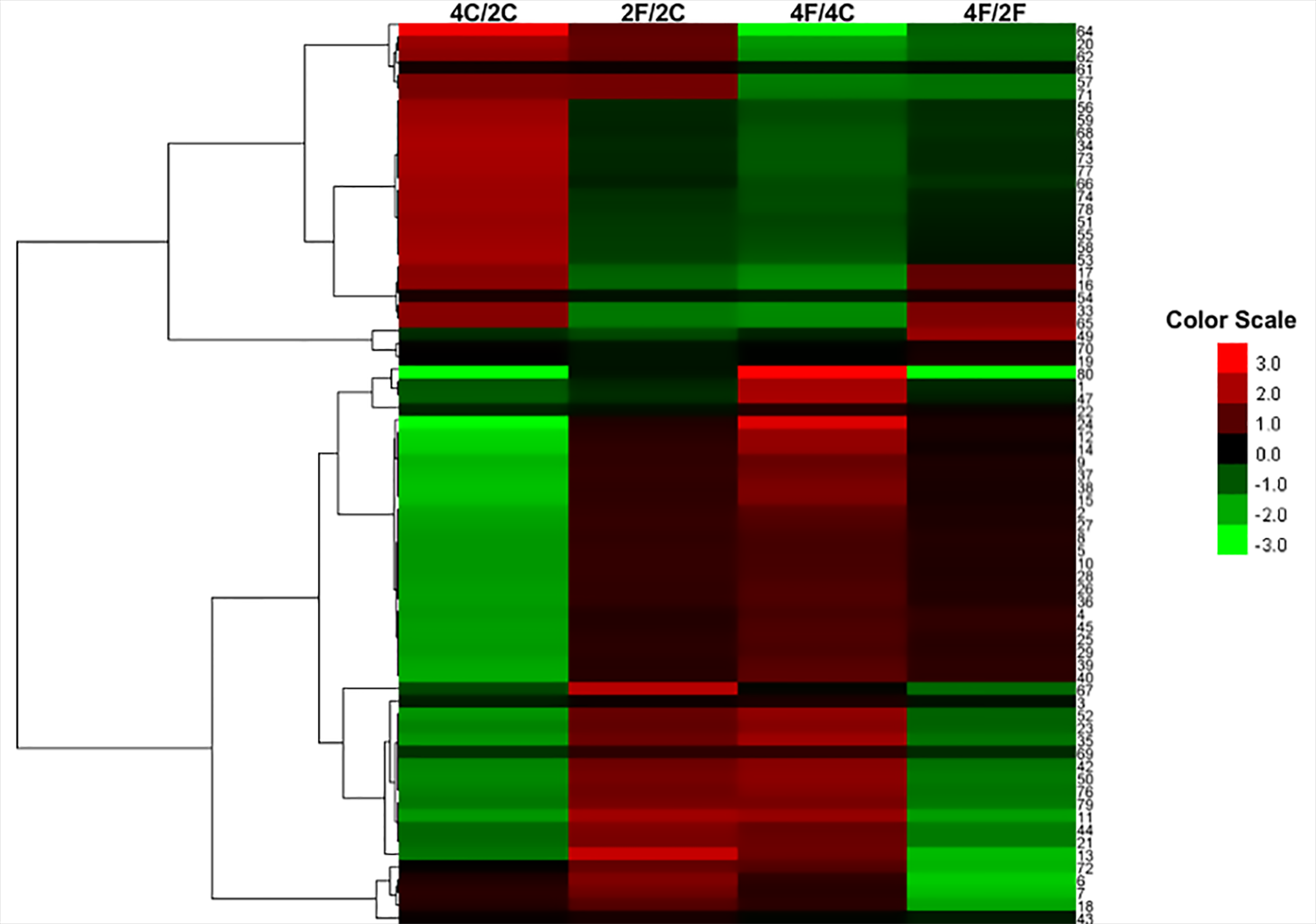

Supplement: Supplementary file 1 [file pathogens-07-00058-s001.zip › Fig S8.tiff]
